# Supplementary figures and images for: Improving the coverage of credible sets in Bayesian genetic fine-mapping
Source: PLoS Comput Biol. 2020 Apr 13;16(4):e1007829. doi: 10.1371/journal.pcbi.1007829 (PMC7179948; doi:10.1371/journal.pcbi.1007829)

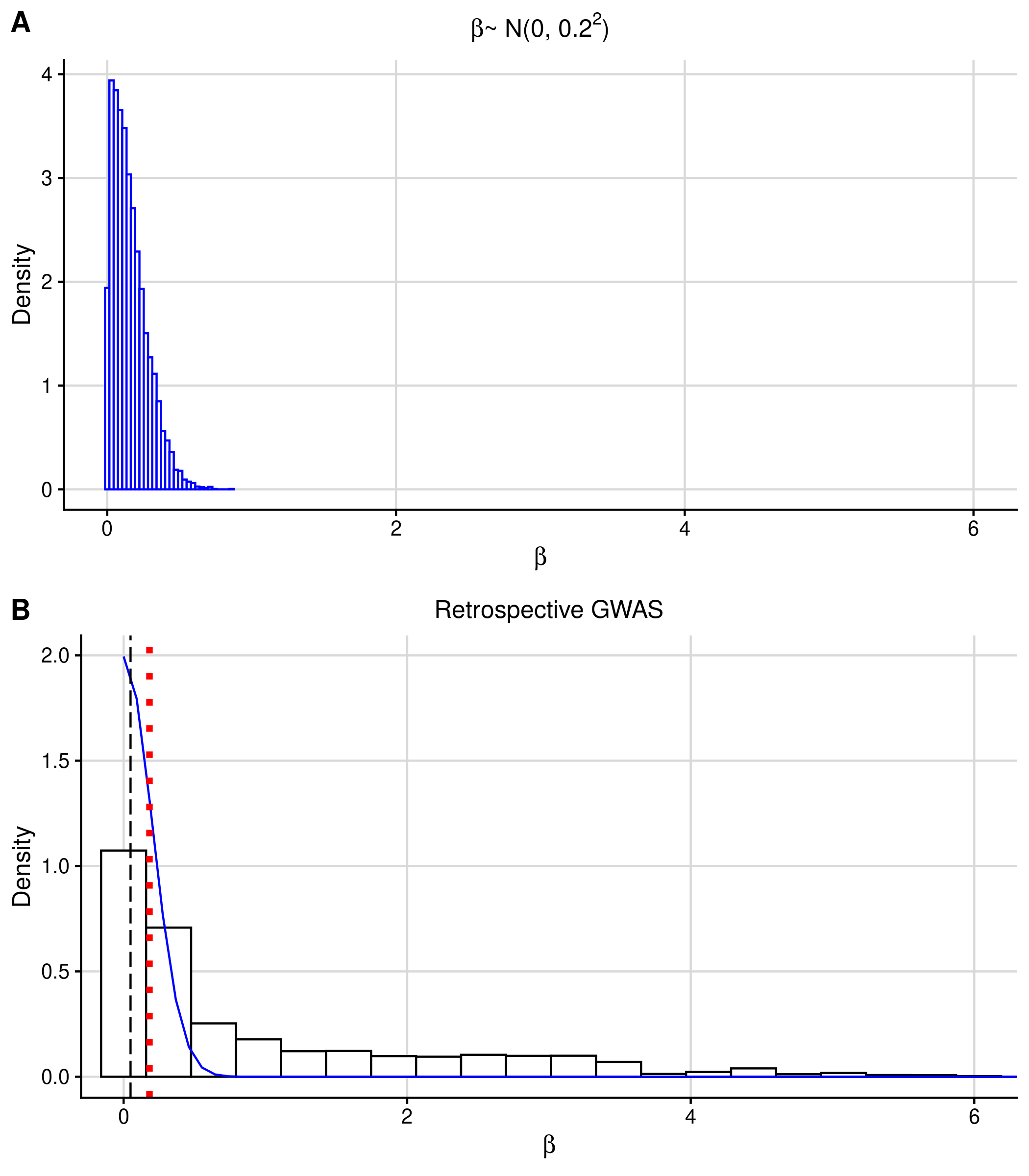

Supplement: S1 Fig — (A) Histogram of absolute effect sizes sampled from the prior, β ∼ N(0, 0.22) (B) Histogram of absolute effect sizes of the lead-SNP in genome-wide significantly associated regions from case-control studies deposited on the GWAS catalog. Blue curve overlaid is for N(0, 0.22) distribution, black dashed line is where β = log(1.05) and red dotted line is where β = log(1.2). The x axis has been truncated to remove extreme values. The distributions are quite different, resulting from censoring of smaller β which are less likely to be associated with a genome-wide significant P value (see Fig 1). (TIF) [file pcbi.1007829.s001.tif]

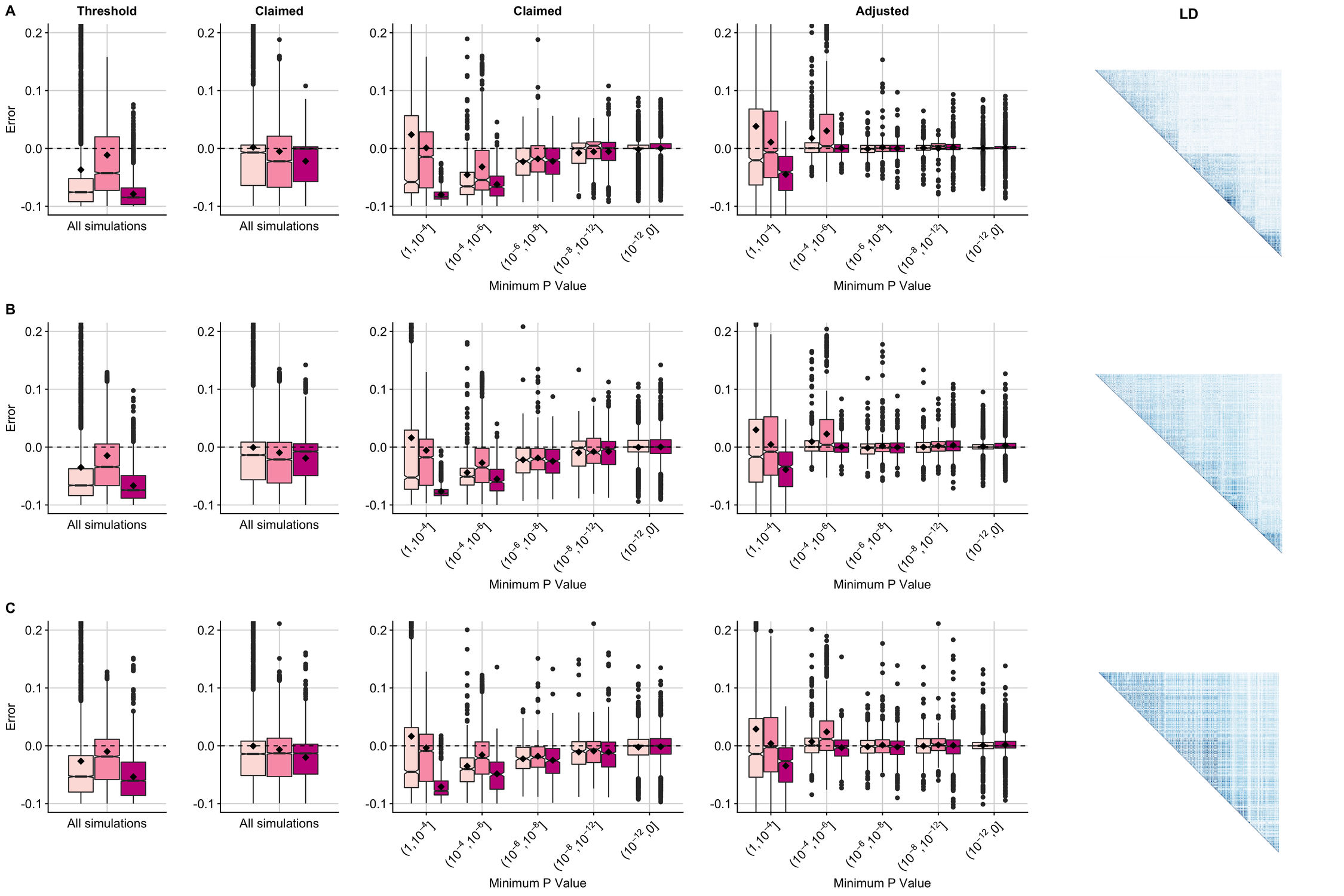

Supplement: S2 Fig — Error is calculated as estimated conditional coverage–empirical conditional coverage where empirical conditional coverage is the proportion of 5000 replicate credible sets that contain the causal variant. Box plots showing error in conditional coverage estimates for 5000 (A) low (B) medium and (C) high LD simulations. Conditional coverage estimates are the threshold (0.9) (left), the claimed coverage (the sum of the posterior probabilities of the variants in the credible set) averaged over all simulations (left-middle) or for simulations binned by minimum P value in the region (right-middle) and the adjusted coverage estimate (right) binned by minimum P value in the region. Black diamond shows mean error. Two simulations for β = log(1.05) simulations that fell into (10−12, 0] bin were manually removed as a box plot could not be generated. Graphical display of SNP correlation matrix for each region shown. (TIF) [file pcbi.1007829.s002.tif]

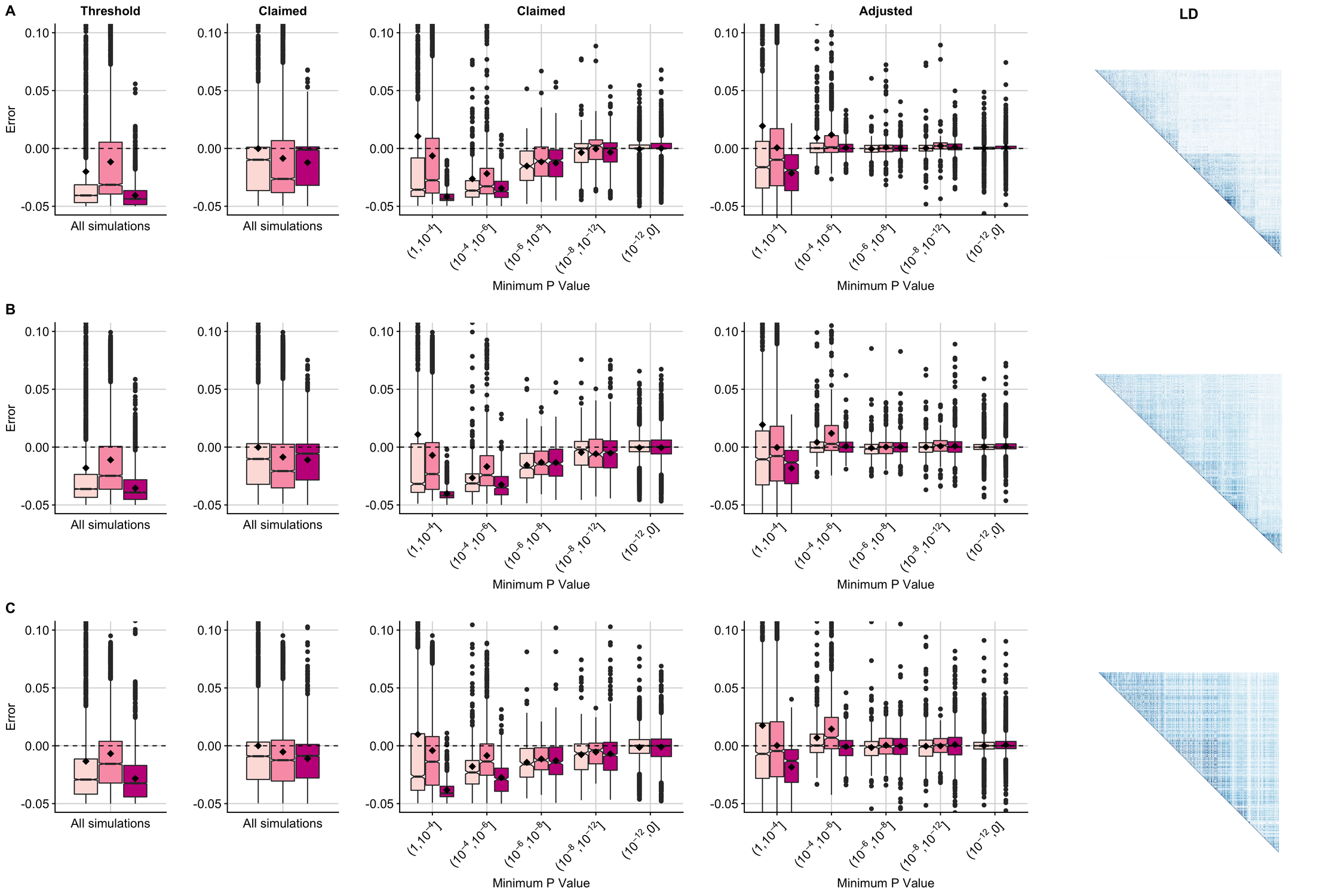

Supplement: S3 Fig — Error is calculated as estimated conditional coverage–empirical conditional coverage where empirical conditional coverage is the proportion of 5000 replicate credible sets that contain the causal variant. Box plots showing error in conditional coverage estimates for 5000 (A) low (B) medium and (C) high LD simulations. Conditional coverage estimates are the threshold (0.95) (left), the claimed coverage (the sum of the posterior probabilities of the variants in the credible set) averaged over all simulations (left-middle) or for simulations binned by minimum P value in the region (right-middle) and the adjusted coverage estimate (right) binned by minimum P value in the region. Black diamond shows mean error. Two simulations for β = log(1.05) simulations that fell into (10−12, 0] bin were manually removed as a box plot could not be generated. Graphical display of SNP correlation matrix for each region shown. (TIF) [file pcbi.1007829.s003.tif]

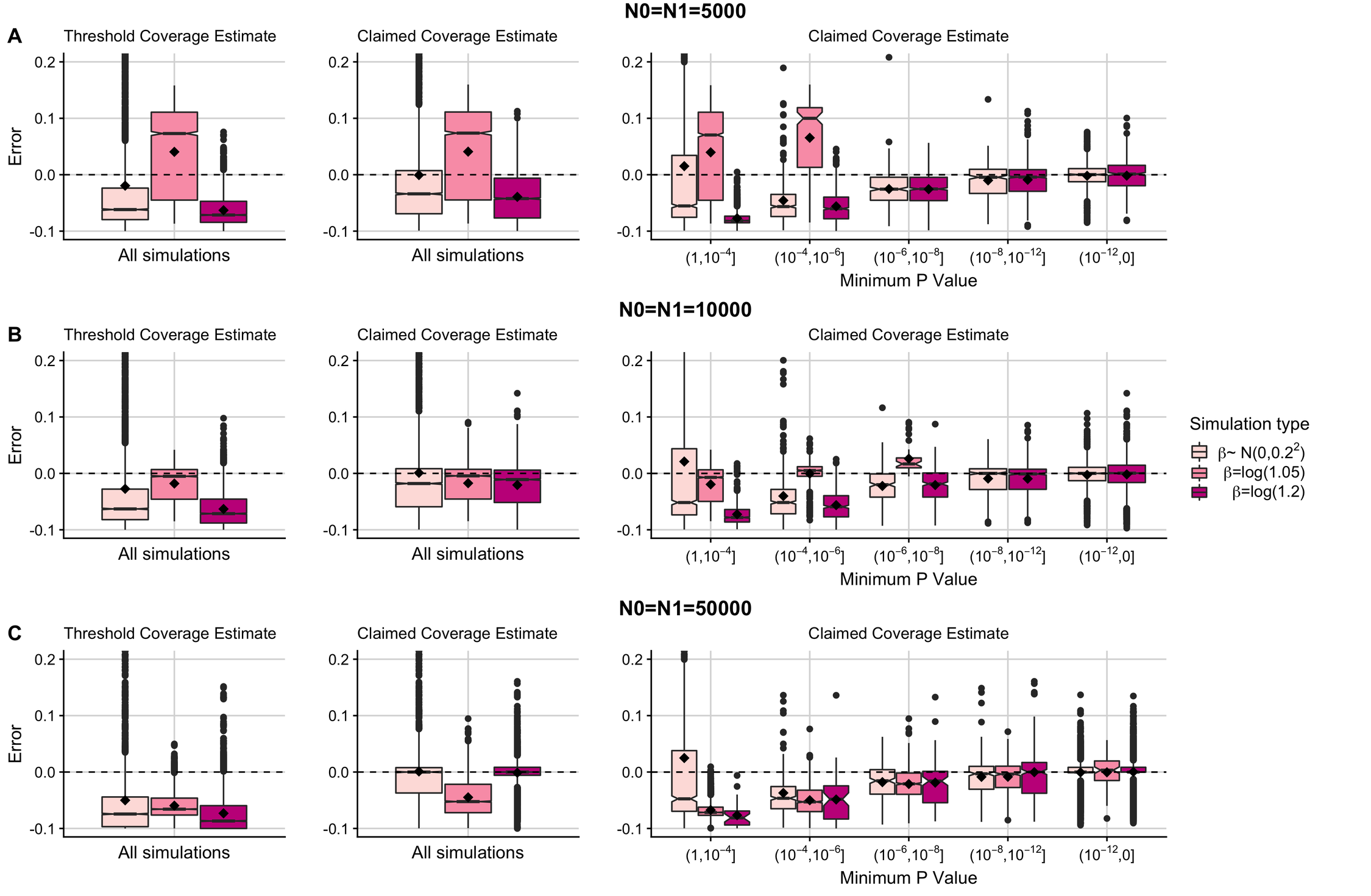

Supplement: S4 Fig — Error is calculated as estimated conditional coverage–empirical conditional coverage where empirical conditional coverage is the proportion of 5000 replicate credible sets that contain the causal variant. Box plots showing error in conditional coverage estimates for 5000 simulations with N0 (number of controls) = N1 (number of cases) = (A) 5000 (B) 10000 and (C) 50000. Overall error in (left) threshold and (middle) claimed coverage estimates averaged across all 5000 simulations. Right hand plots show error in claimed coverage estimates for different P value bins. If there were <10 simulations contained in a P value bin, then these were manually removed (for example in Pmin < 10−6 bins for β = log(1.05), N0 = N1 = 5000). (TIF) [file pcbi.1007829.s004.tif]

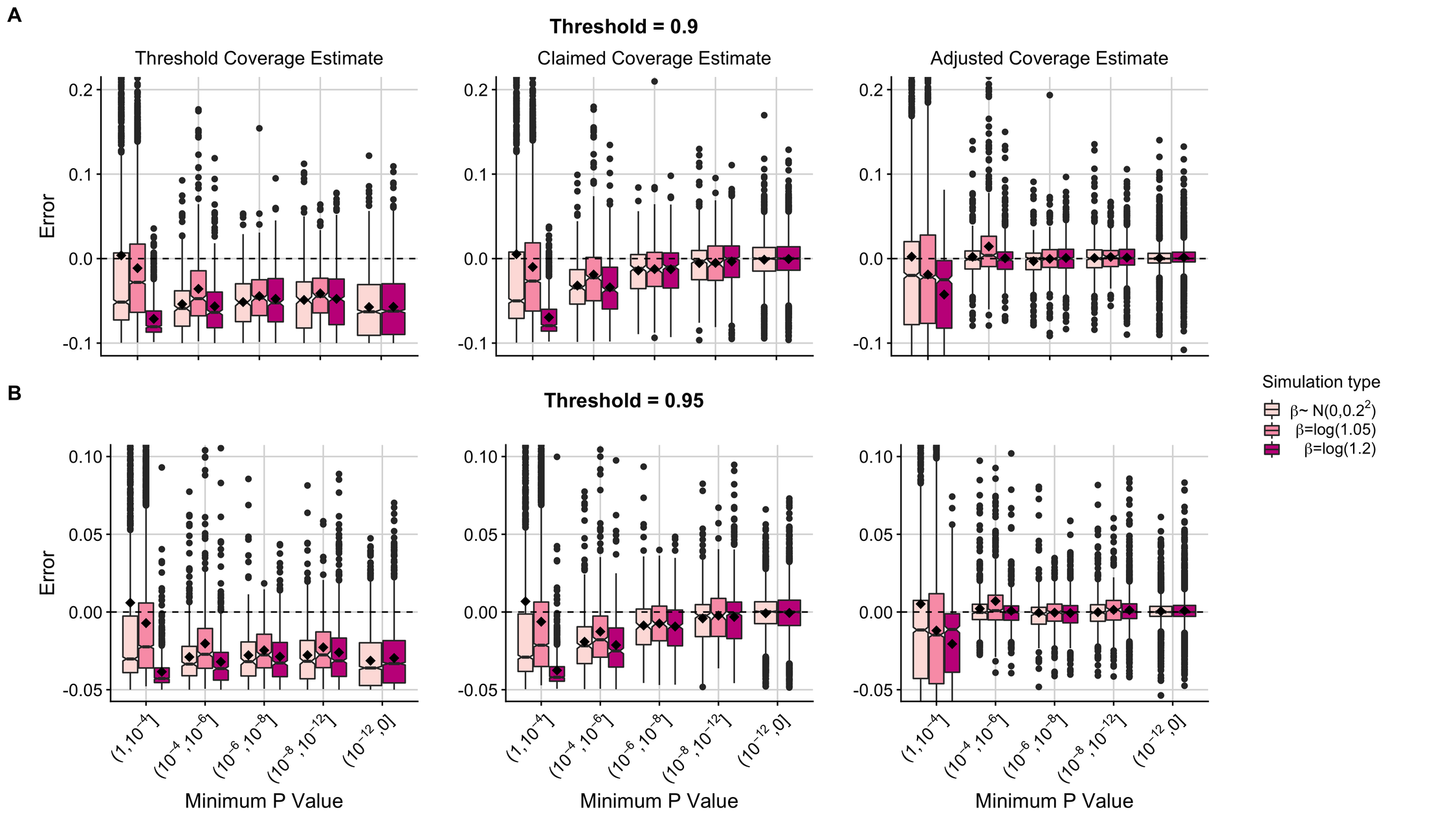

Supplement: S5 Fig — Box plots showing the error, estimated conditional coverage–empirical conditional coverage, of (A) 90% and (B) 95% credible sets where empirical conditional coverage is the proportion of 5000 replicate credible sets that contain the causal variant. Error of conditional coverage estimates where (left) conditional coverage estimate equals threshold, (middle) conditional coverage estimate equals claimed coverage (sum of the posterior probabilities of the variants in the set) and (right) conditional coverage estimate is adjusted coverage. Results from 5000 simulations for each simulation type have been averaged over many genomic regions that vary in LD patterns. (TIF) [file pcbi.1007829.s005.tif]

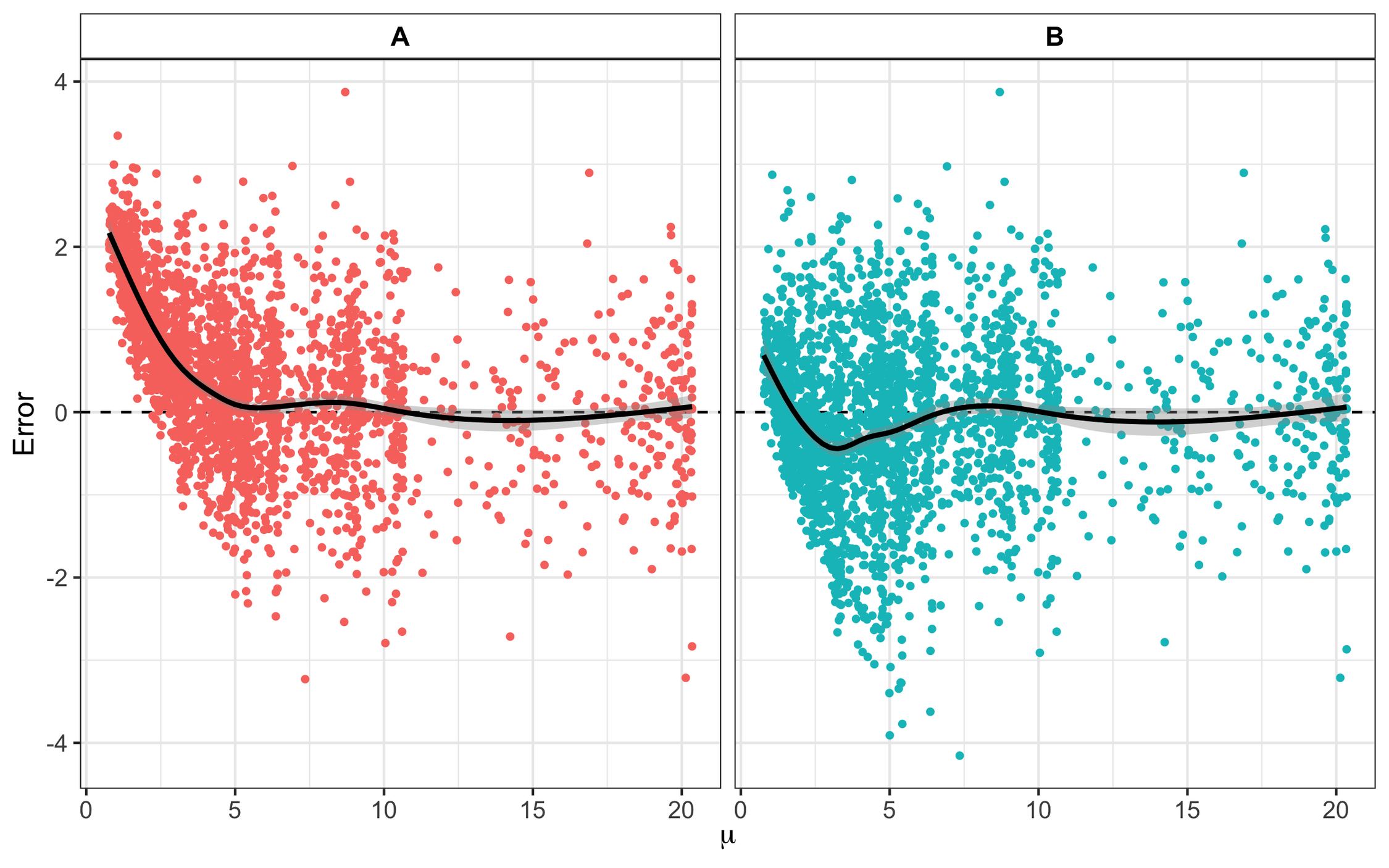

Supplement: S6 Fig — Error of μ estimates calculated as μ^X-μ. The x axis is the joint Z score at the CV. Line is fitted using a GAM as the smoothing function (geom_smooth() in ggplot2). (A) μ^=maxi∈{1,...,k}(|Zi|) (B) μ^=∑i=1k|Zi|×PPi. (TIF) [file pcbi.1007829.s006.tif]

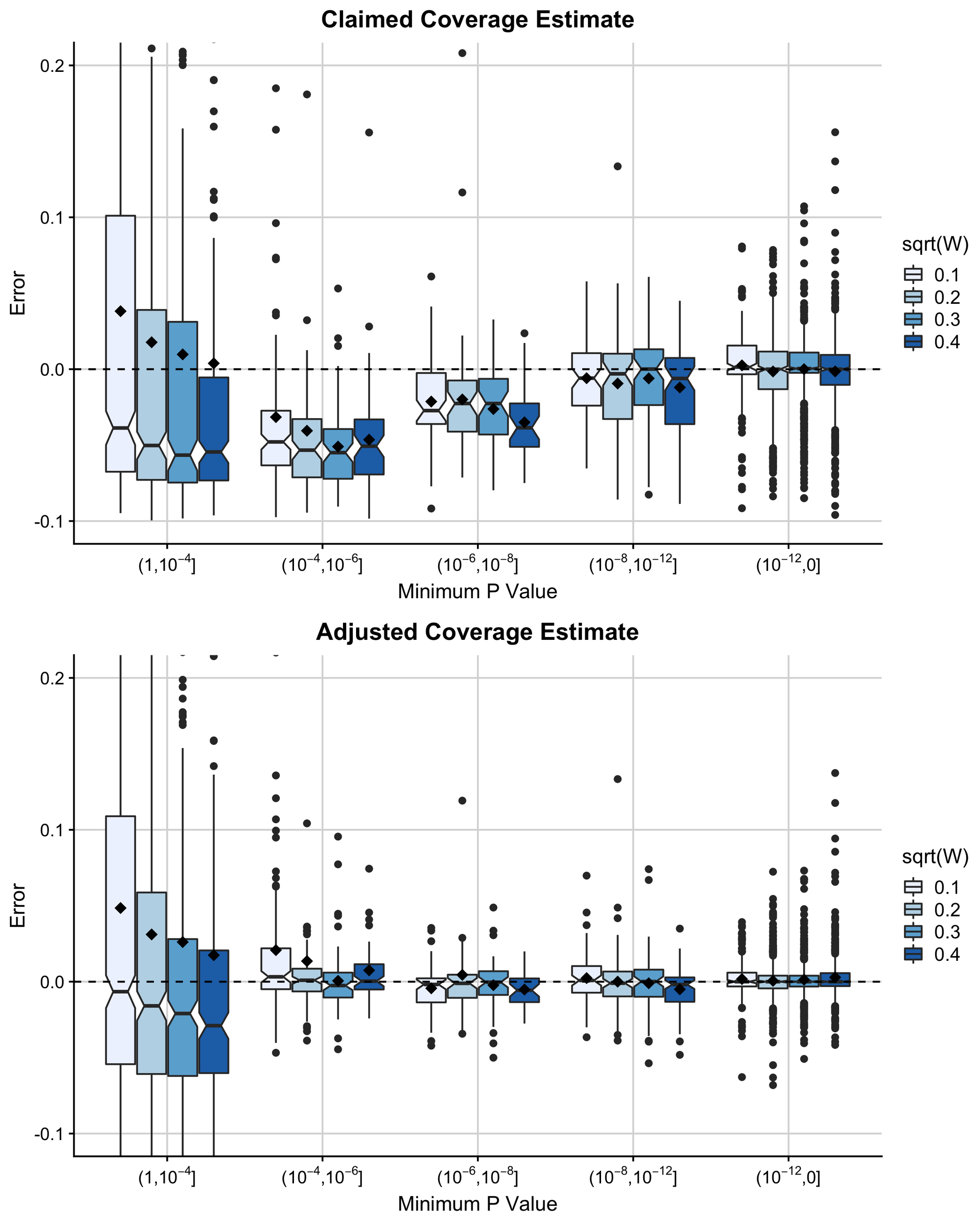

Supplement: S7 Fig — Error is calculated as estimated conditional coverage–empirical conditional coverage where empirical conditional coverage is the proportion of 5000 replicate credible sets that contain the causal variant. Box plots showing error in conditional coverage estimates for 5000 medium LD simulations. Conditional coverage estimates are the claimed coverage (top) and the adjusted coverage estimate (bottom) for simulations binned by minimum P value in the region. Black diamond shows mean error. (TIF) [file pcbi.1007829.s007.tif]

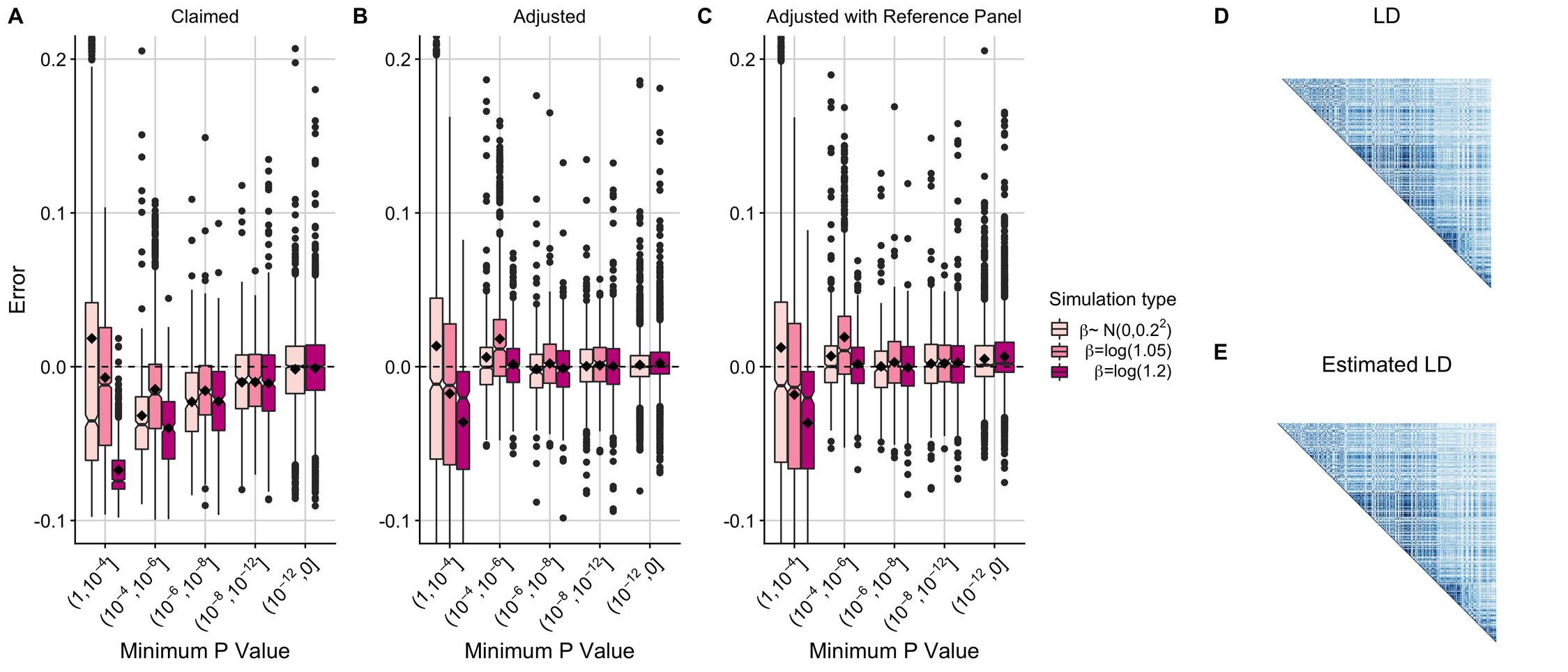

Supplement: S8 Fig — Error is calculated as estimated conditional coverage–empirical conditional coverage. Conditional coverage estimates from 5000 simulations using original 1000 Genomes data and UK10K data as a reference panel. (A) Claimed coverage estimate (the sum of the posterior probabilities of causality for the variants in the credible set) (B) Adjusted coverage estimate (C) Adjusted coverage estimate using UK10K data to approximate MAFs and SNP correlations (D) Graphical display of SNP correlations in 1000 Genomes data (E) Graphical display of the estimated SNP correlations in UK10K data. Two simulations for β = log(1.05) simulations that fell into (10−12, 0] bin were manually removed as a box plot could not be generated. (TIF) [file pcbi.1007829.s008.tif]

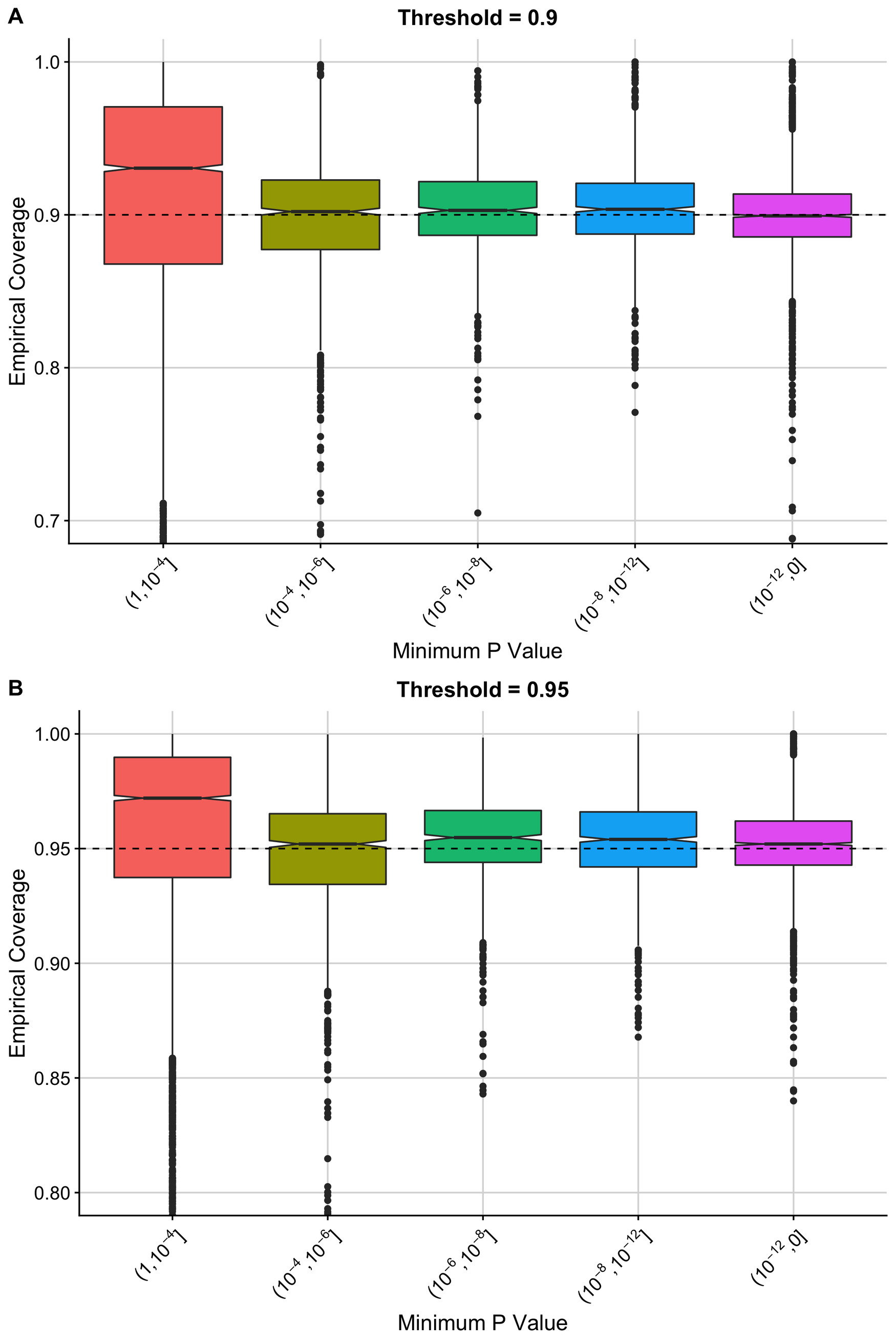

Supplement: S9 Fig — 100,000 simulated (A) 90% and (B) 95% credible sets were adjusted using the corrcoverage::corrected_cs function (with default parameters and ‘desired.cov = 0.9’ or ‘desired.cov = 0.95’), and the “required threshold” value obtained from each simulation was used to form 5000 replicate credible sets to estimate the empirical conditional coverage of these adjusted (A) 90% and (B) 95% credible sets. (TIF) [file pcbi.1007829.s009.tif]

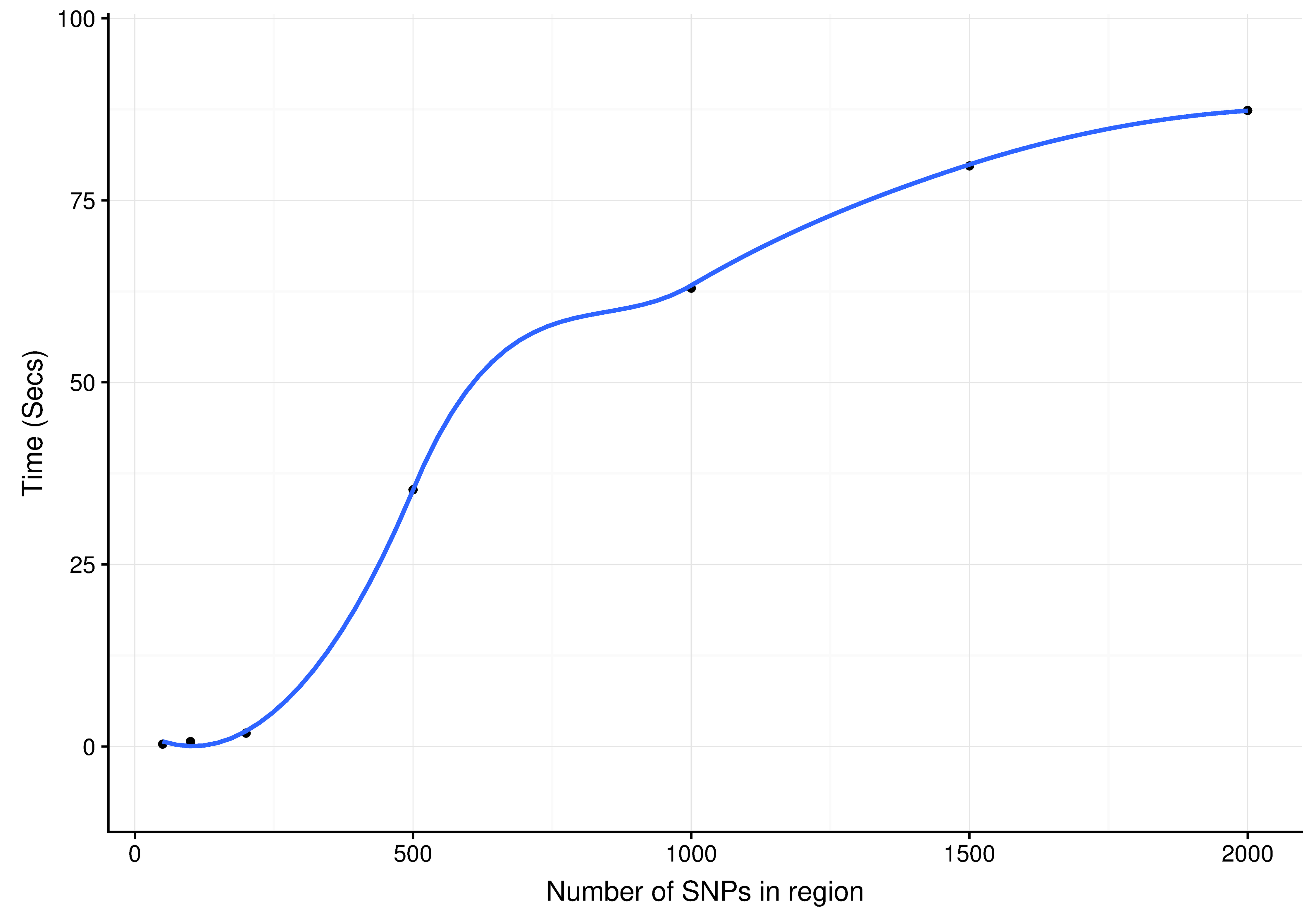

Supplement: S10 Fig — Curve showing the timings of the corrcoverage::corrcov function for different sized genomic regions. For each size of genomic region analysed, 50 replicates of the corrcoverage::corrcov function were ran and the mean time taken is plotted. Curve drawn using geom_smooth() function in ggplot2. Simulations ran using one core of an Intel Xeon Gold 6142 processor running at 2.6GHz. (TIF) [file pcbi.1007829.s010.tif]

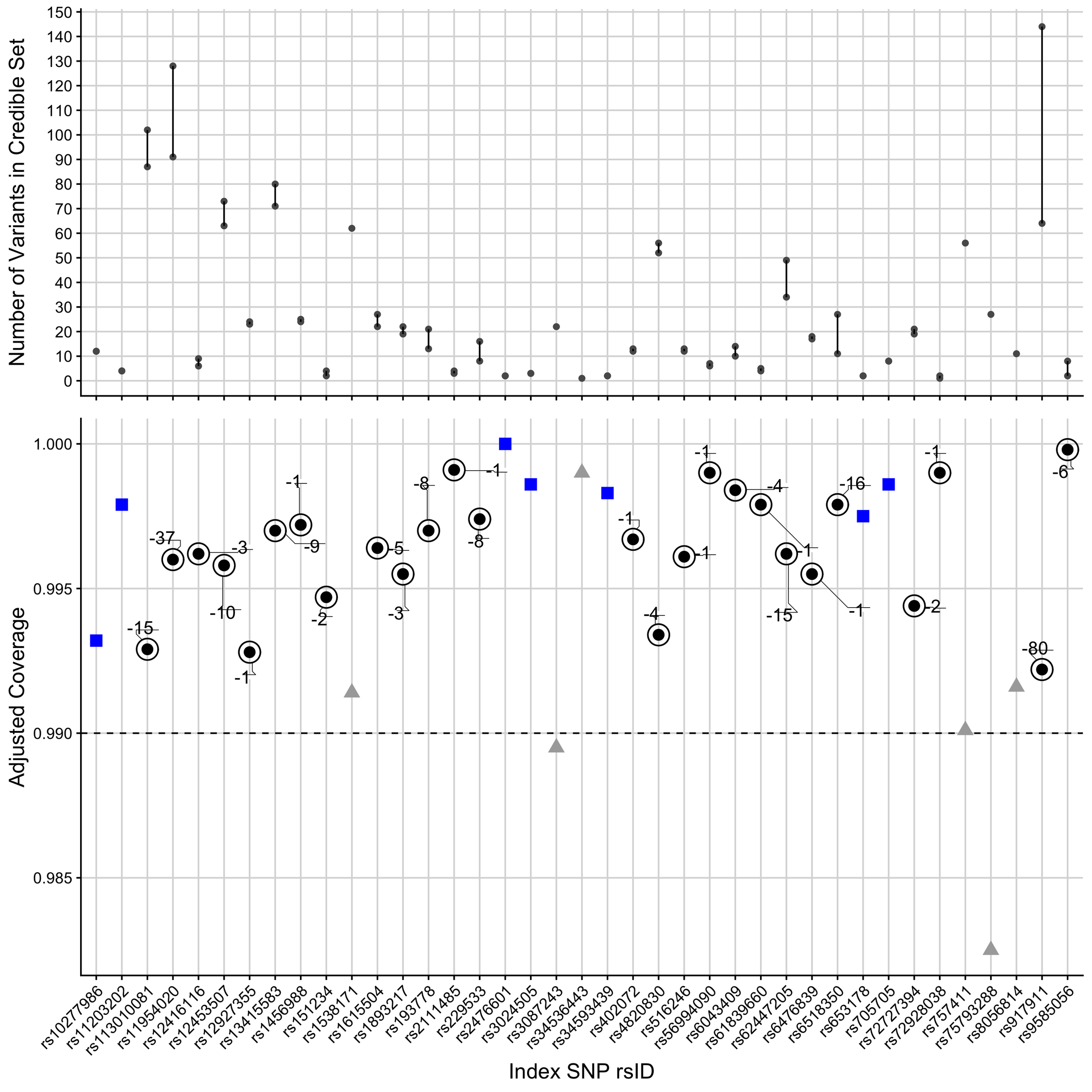

Supplement: S11 Fig — Top panel: The decrease in size of the credible set after adjustment. Bottom panel: The adjusted coverage estimates of 99% Bayesian credible sets for T1D-associated genomic regions. Black points represents regions where the credible set changed after the adjustment and the “-” values for the circled points represent the decrease in the number of variants from the standard to the adjusted 99% credible set. Blue points represent regions where the credible set did not change after the adjustment and grey points represent regions where the credible set did not need to be adjusted since the threshold was contained in the 99% confidence interval of the coverage estimate, or because the credible set already contained only a single variant. (TIF) [file pcbi.1007829.s011.tif]

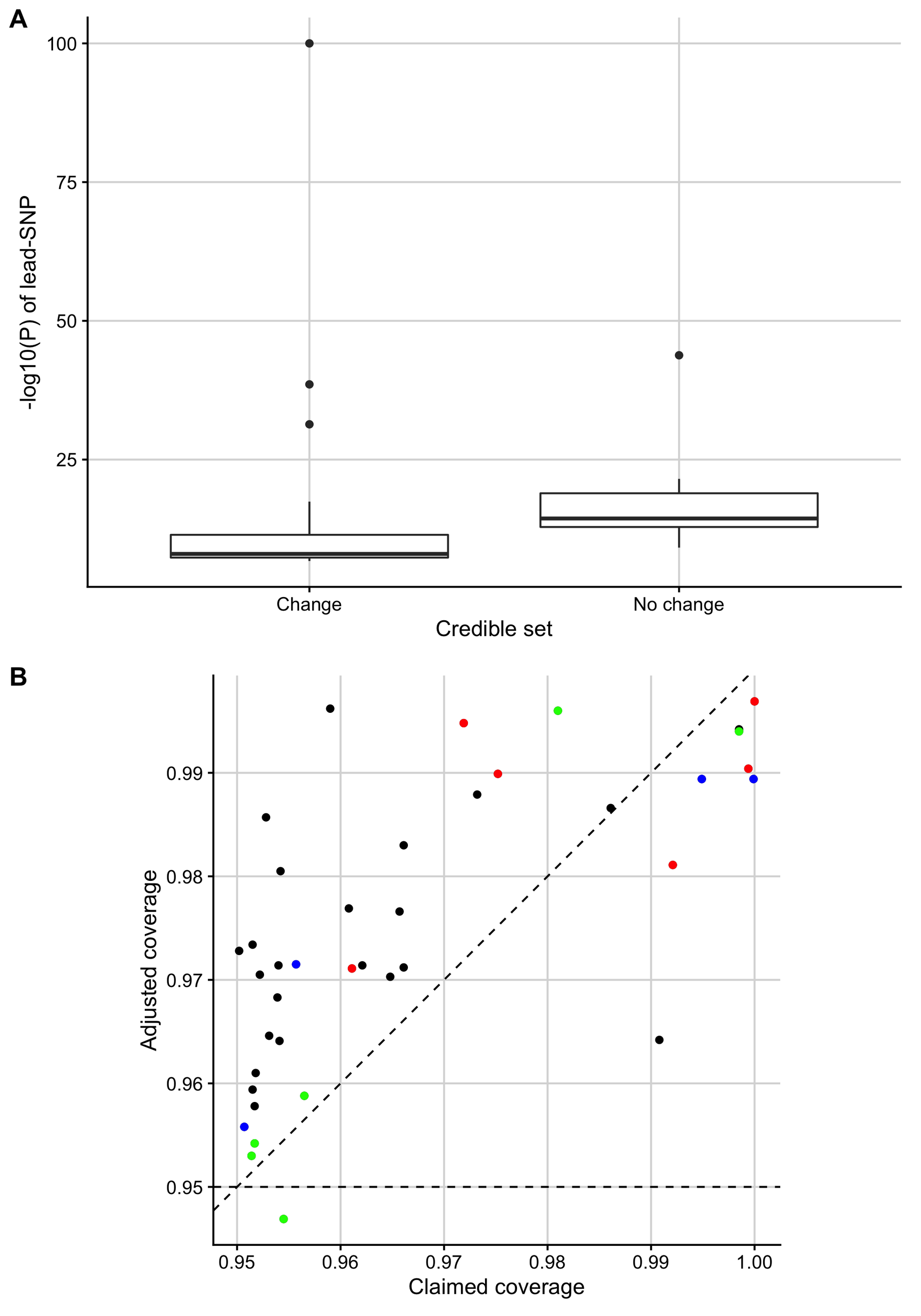

Supplement: S12 Fig — (A) Box plots showing the distribution of lead-SNP −log10(P) values for genomic regions where the 95% credible set changed after adjustment or where it did not change after adjustment. (B) Adjusted coverage estimates against claimed coverage estimates of standard 95% credible sets for 39 genomic regions associated with T1D. Red points are those with lead-SNP P < 10−12 and where the credible set changed after adjustment, blue points are those with lead-SNP P < 10−12 and where the credible set did not change after adjustment and green points are those with lead-SNP P < 10−12 whose 95% credible set did not need to be adjusted. (TIF) [file pcbi.1007829.s012.tif]

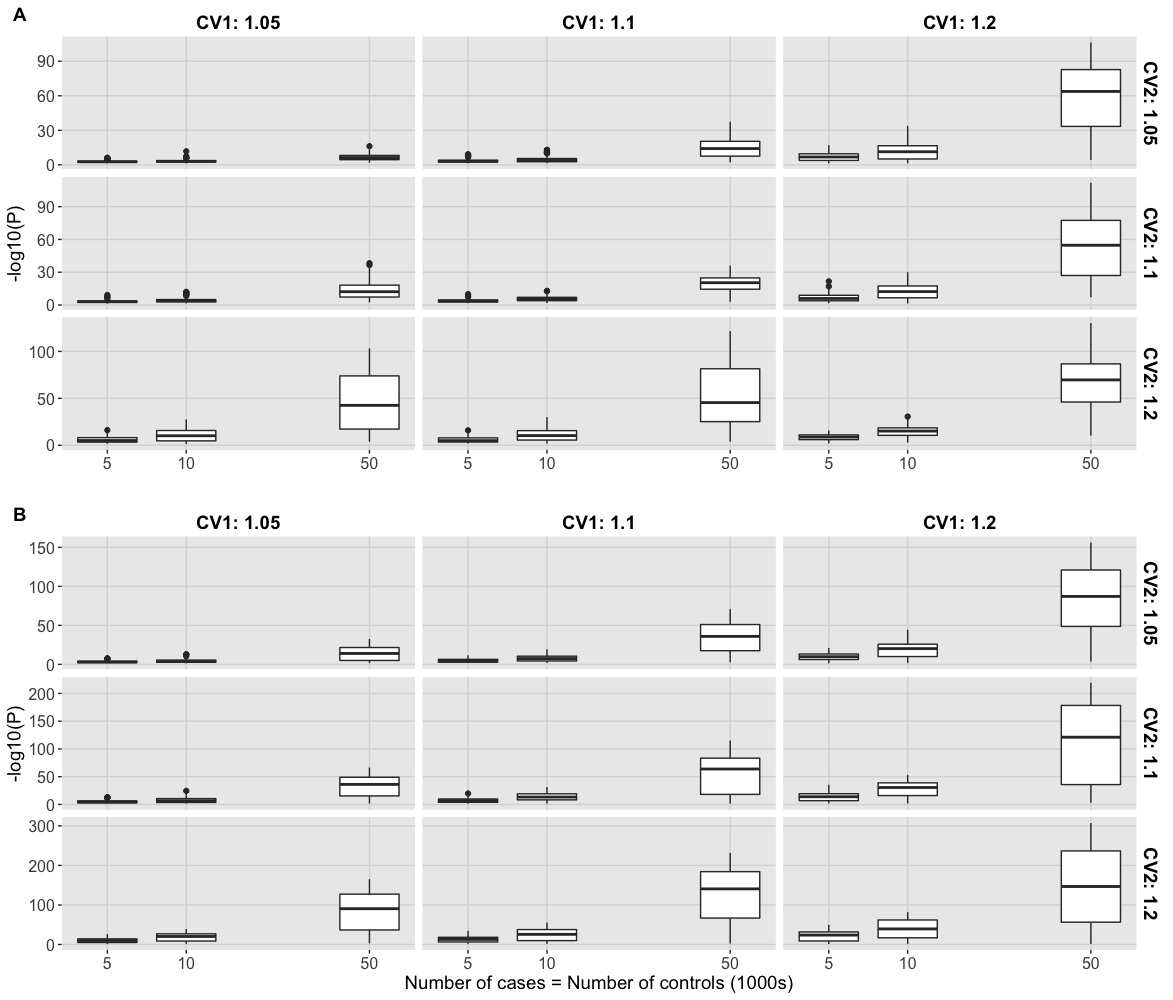

Supplement: S13 Fig — 2 CVs are (A) in low LD (r2 < 0.01) (B) in high LD (r2 > 0.7). Faceted by odds ratio values at the causal variants. (TIF) [file pcbi.1007829.s013.tif]

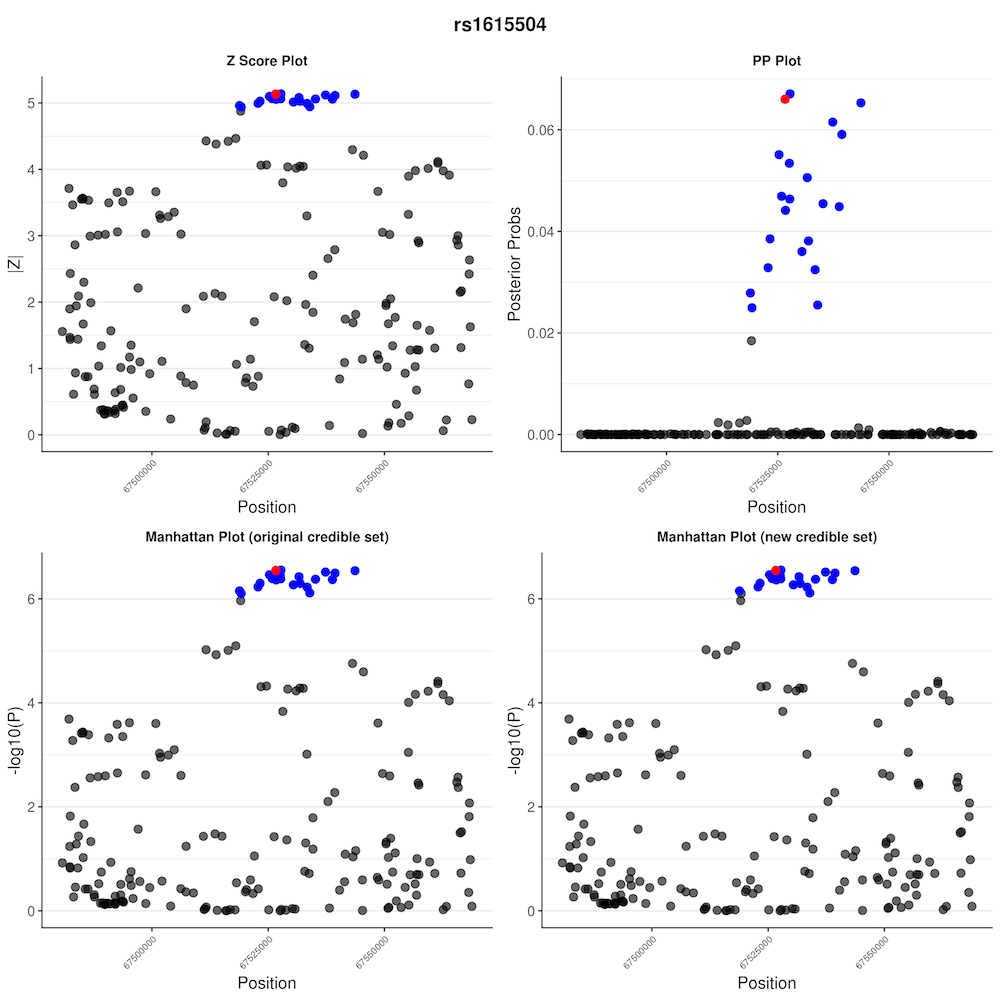

Supplement: S1 File — Zip file containing Z-score plots, PP plots and Manhattan plots for the 39 T1D association regions analysed. (ZIP) [file pcbi.1007829.s014.zip › S1_file/rs1615504.png]

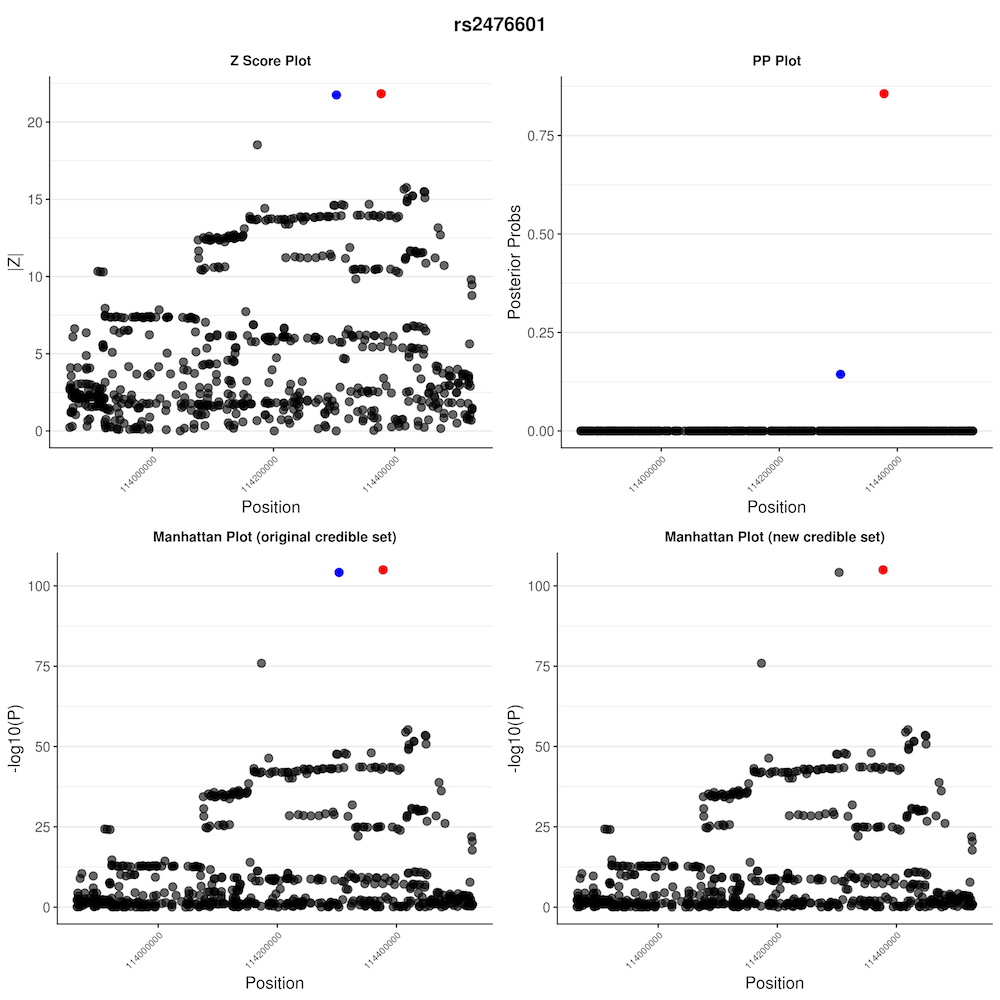

Supplement: S1 File — Zip file containing Z-score plots, PP plots and Manhattan plots for the 39 T1D association regions analysed. (ZIP) [file pcbi.1007829.s014.zip › S1_file/rs2476601.png]

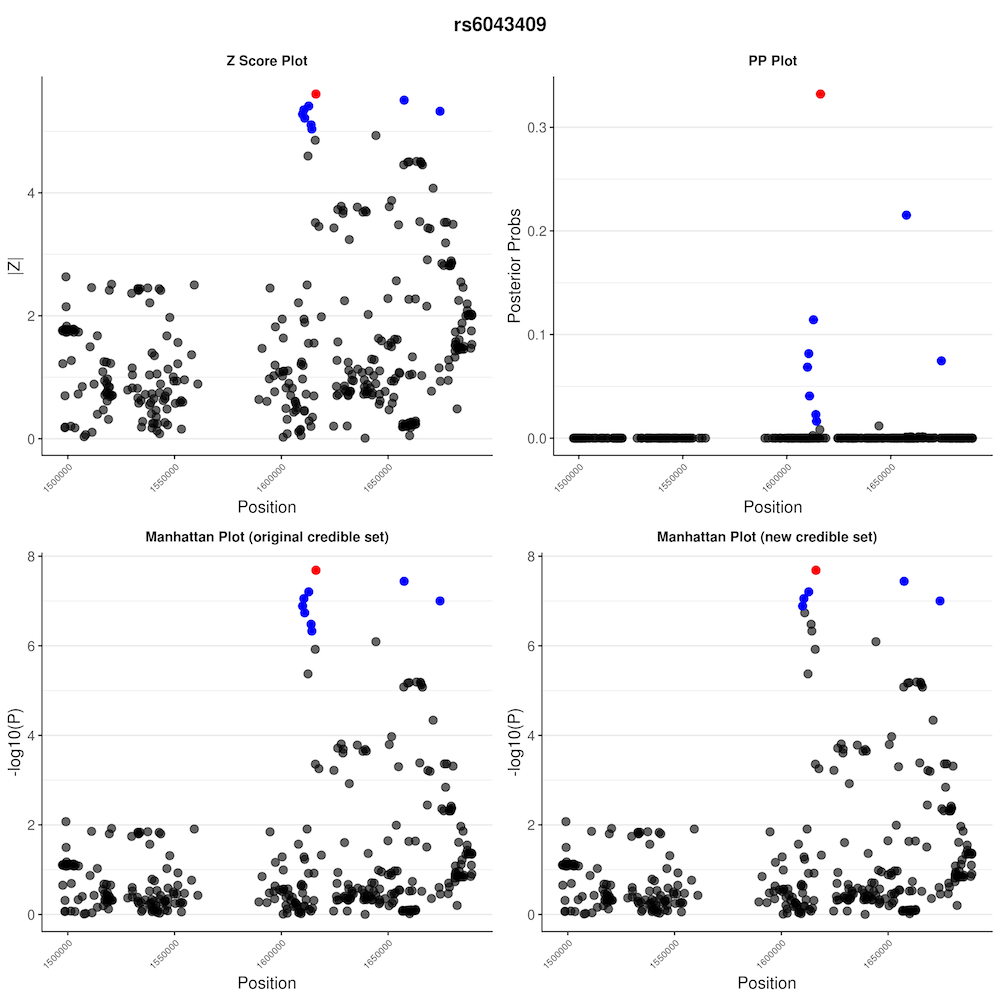

Supplement: S1 File — Zip file containing Z-score plots, PP plots and Manhattan plots for the 39 T1D association regions analysed. (ZIP) [file pcbi.1007829.s014.zip › S1_file/rs6043409.png]

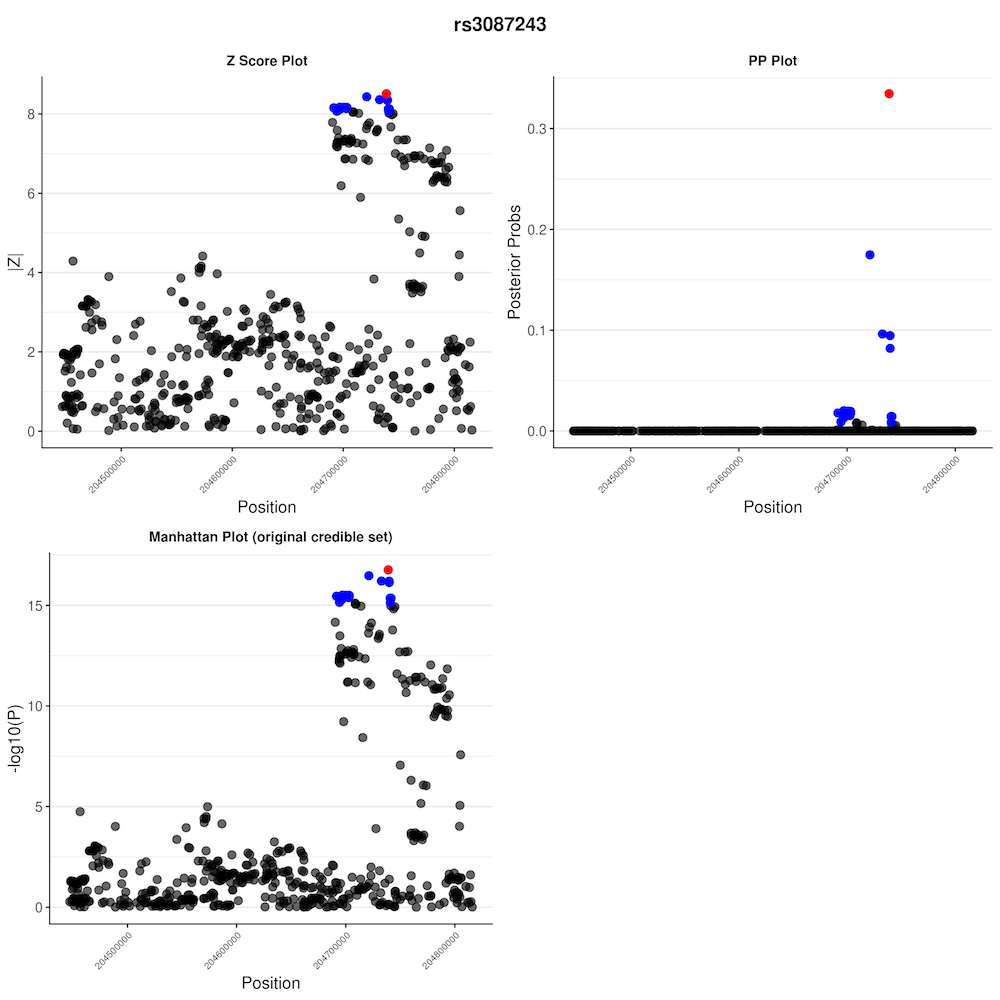

Supplement: S1 File — Zip file containing Z-score plots, PP plots and Manhattan plots for the 39 T1D association regions analysed. (ZIP) [file pcbi.1007829.s014.zip › S1_file/rs3087243.png]

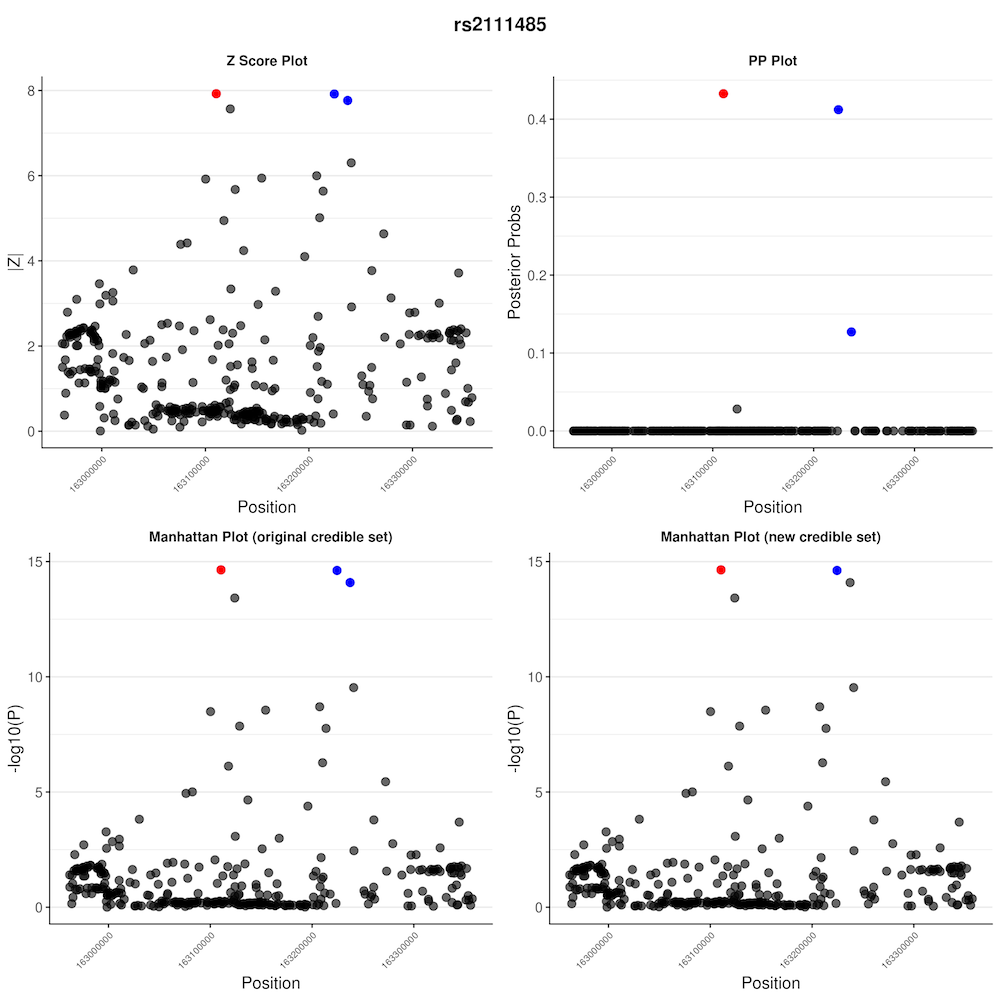

Supplement: S1 File — Zip file containing Z-score plots, PP plots and Manhattan plots for the 39 T1D association regions analysed. (ZIP) [file pcbi.1007829.s014.zip › S1_file/rs2111485.png]

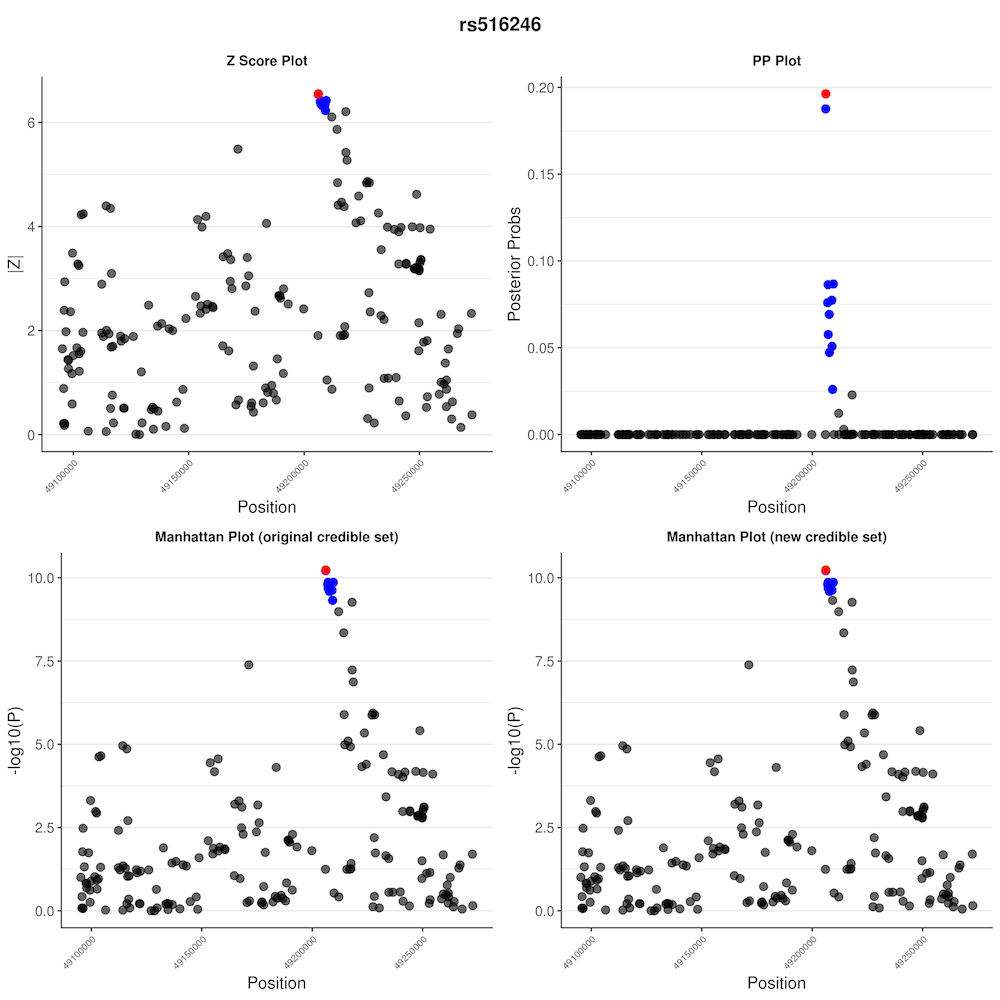

Supplement: S1 File — Zip file containing Z-score plots, PP plots and Manhattan plots for the 39 T1D association regions analysed. (ZIP) [file pcbi.1007829.s014.zip › S1_file/rs516246.png]

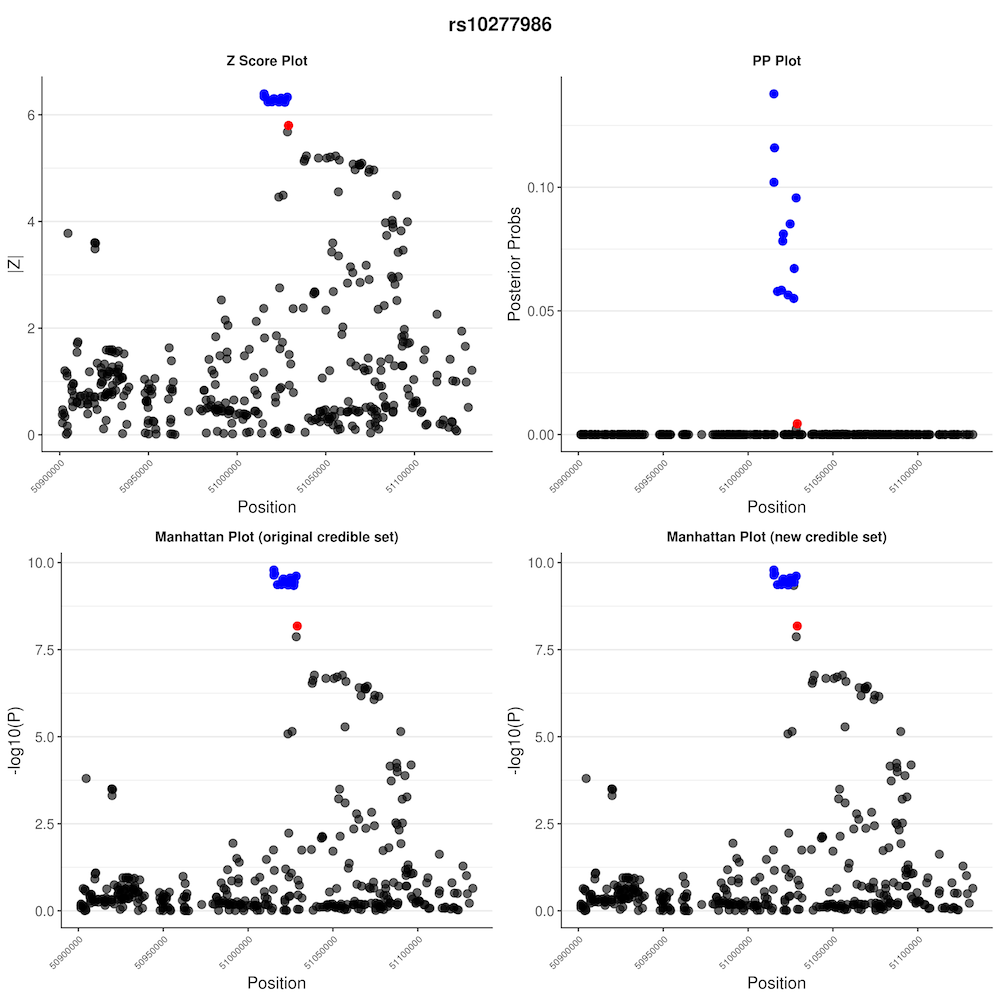

Supplement: S1 File — Zip file containing Z-score plots, PP plots and Manhattan plots for the 39 T1D association regions analysed. (ZIP) [file pcbi.1007829.s014.zip › S1_file/rs10277986.png]

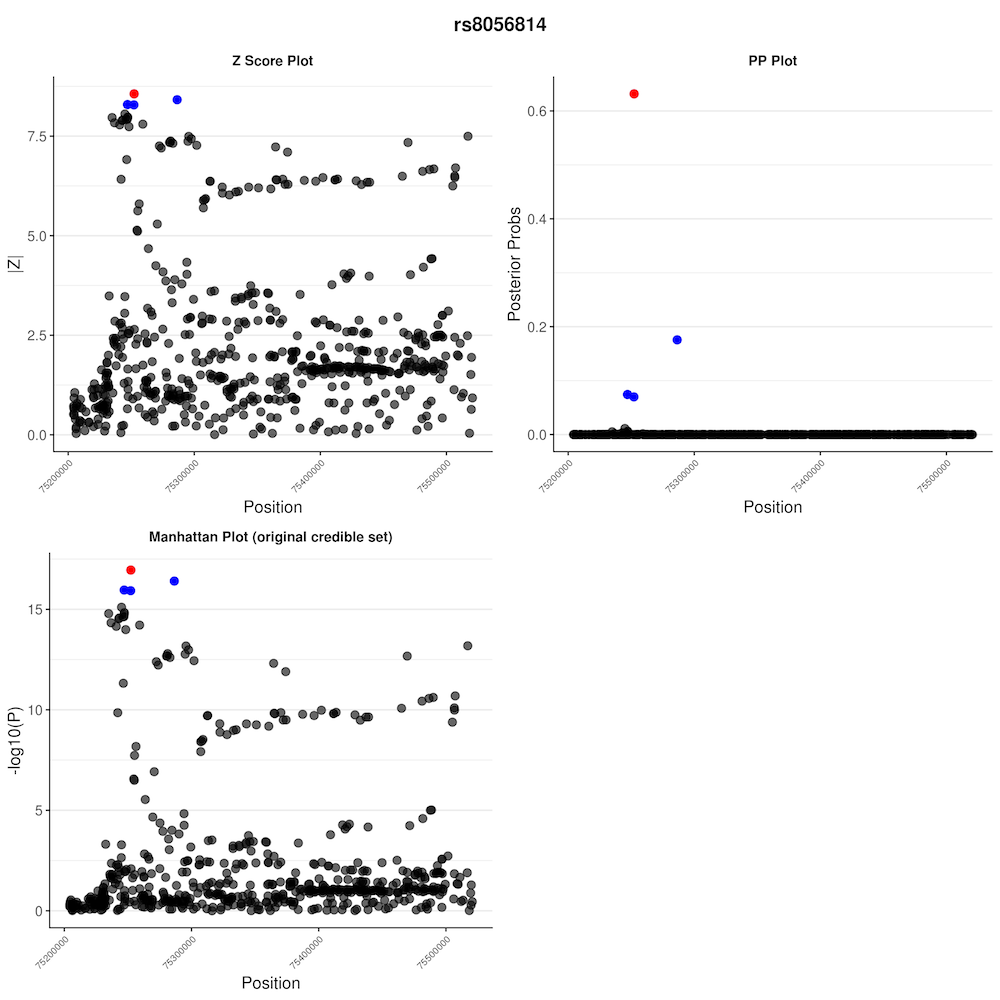

Supplement: S1 File — Zip file containing Z-score plots, PP plots and Manhattan plots for the 39 T1D association regions analysed. (ZIP) [file pcbi.1007829.s014.zip › S1_file/rs8056814.png]

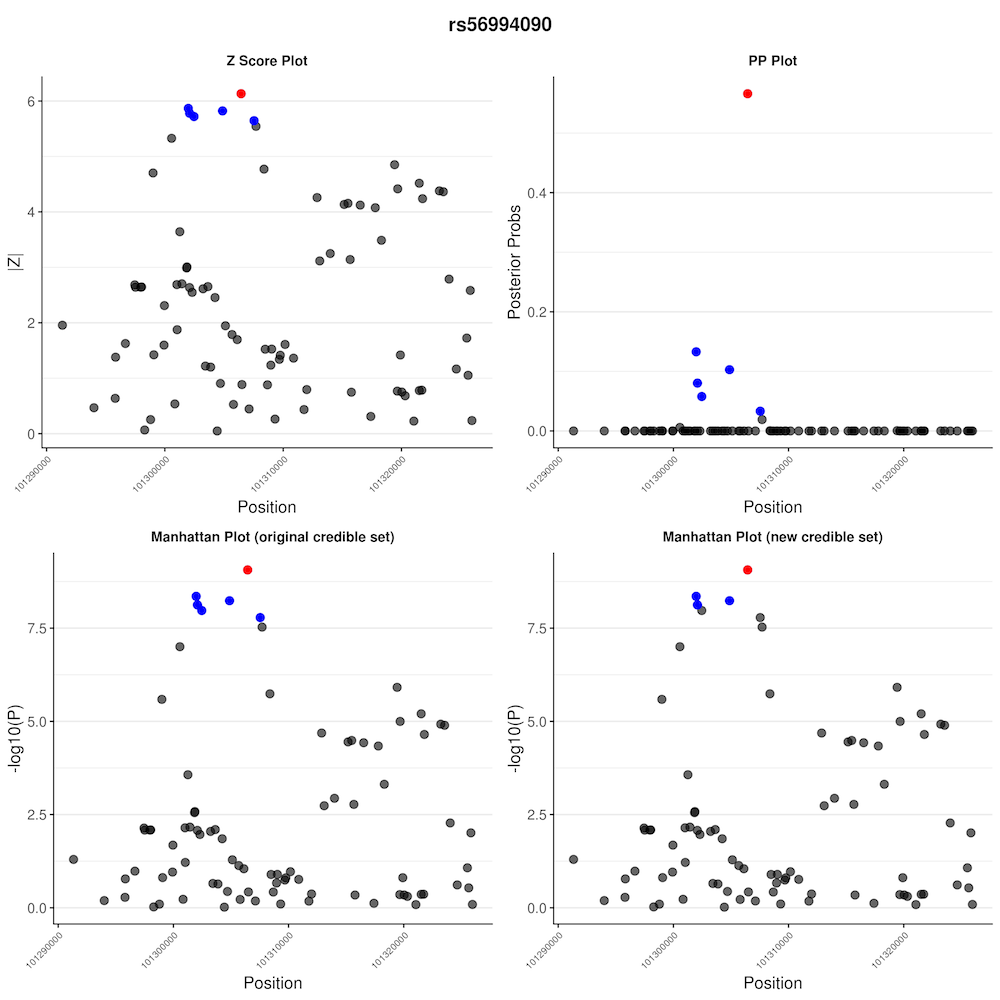

Supplement: S1 File — Zip file containing Z-score plots, PP plots and Manhattan plots for the 39 T1D association regions analysed. (ZIP) [file pcbi.1007829.s014.zip › S1_file/rs56994090.png]

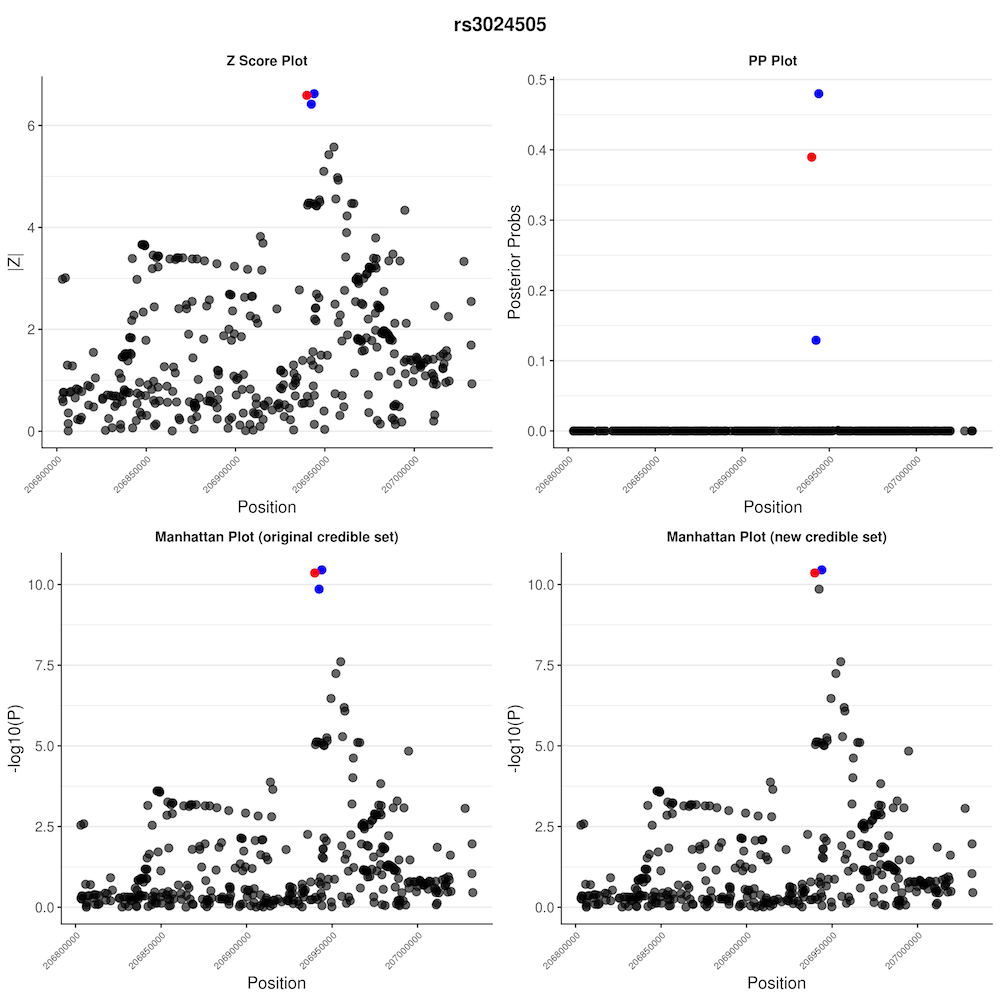

Supplement: S1 File — Zip file containing Z-score plots, PP plots and Manhattan plots for the 39 T1D association regions analysed. (ZIP) [file pcbi.1007829.s014.zip › S1_file/rs3024505.png]

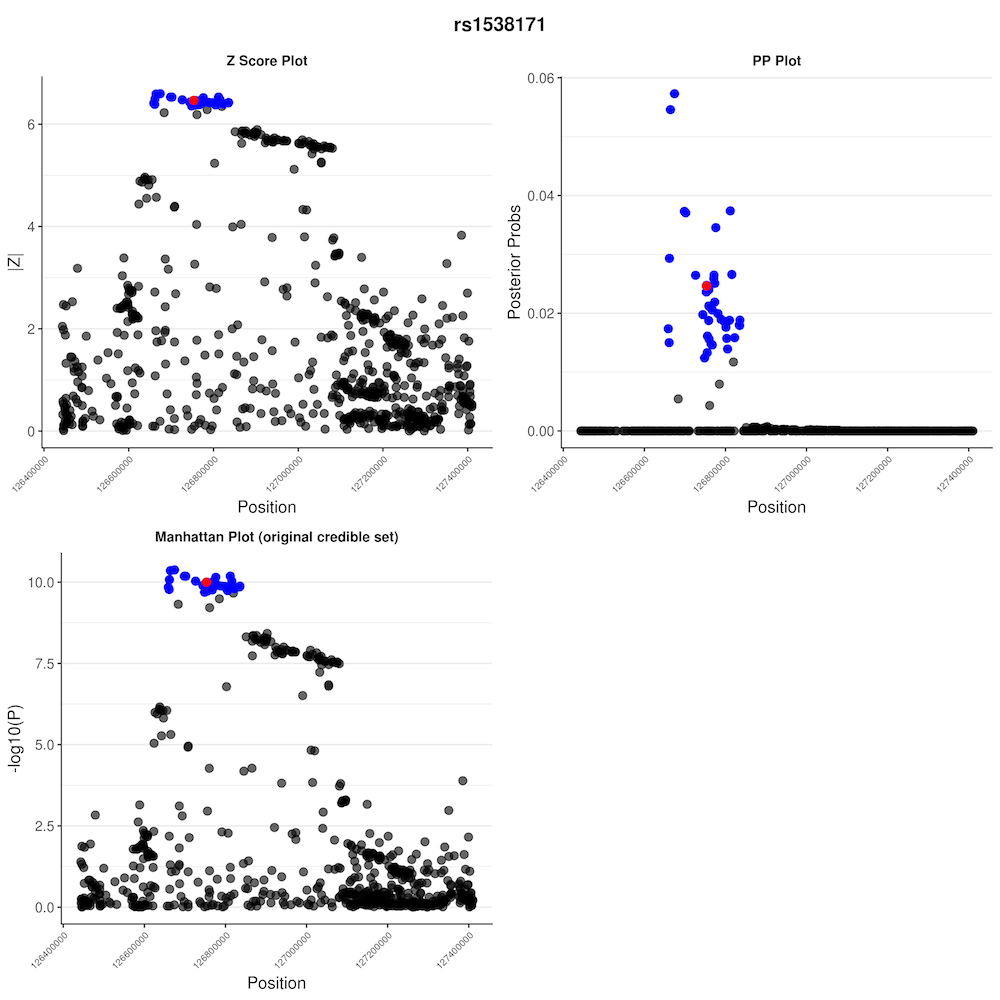

Supplement: S1 File — Zip file containing Z-score plots, PP plots and Manhattan plots for the 39 T1D association regions analysed. (ZIP) [file pcbi.1007829.s014.zip › S1_file/rs1538171.png]

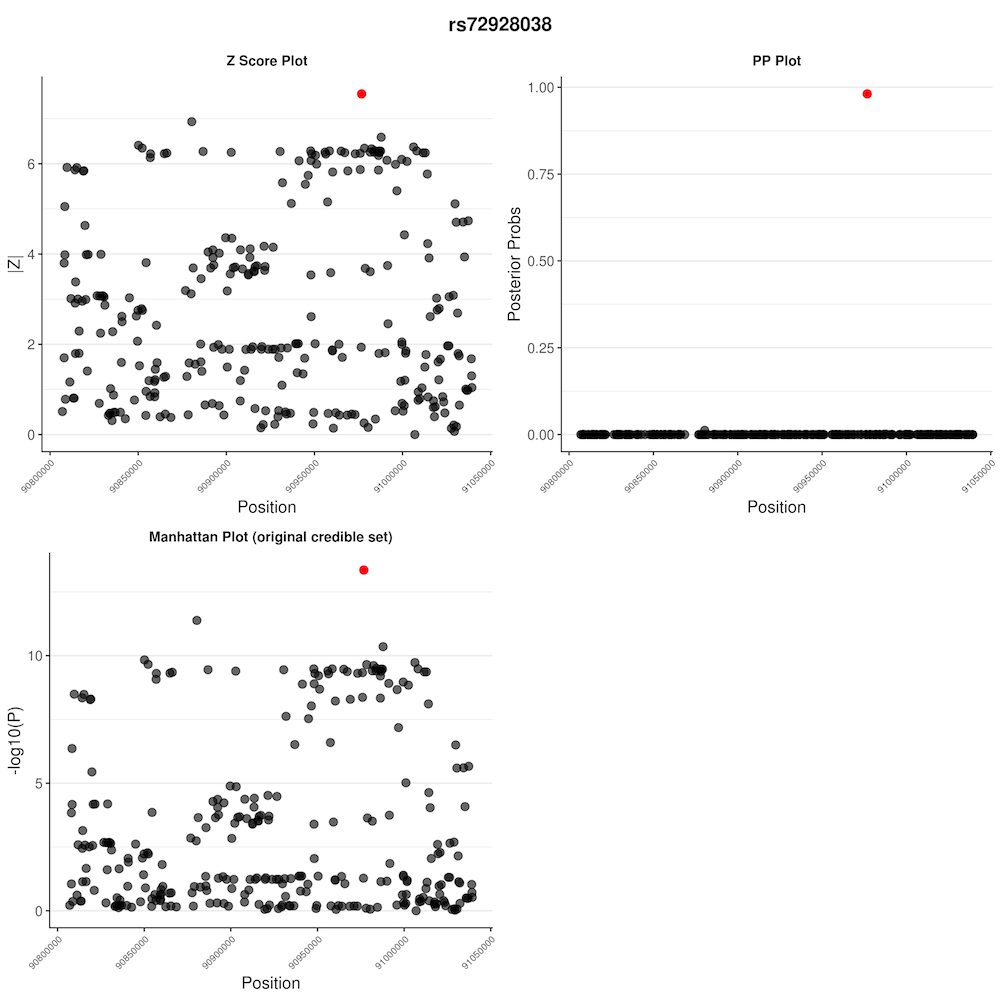

Supplement: S1 File — Zip file containing Z-score plots, PP plots and Manhattan plots for the 39 T1D association regions analysed. (ZIP) [file pcbi.1007829.s014.zip › S1_file/rs72928038.png]

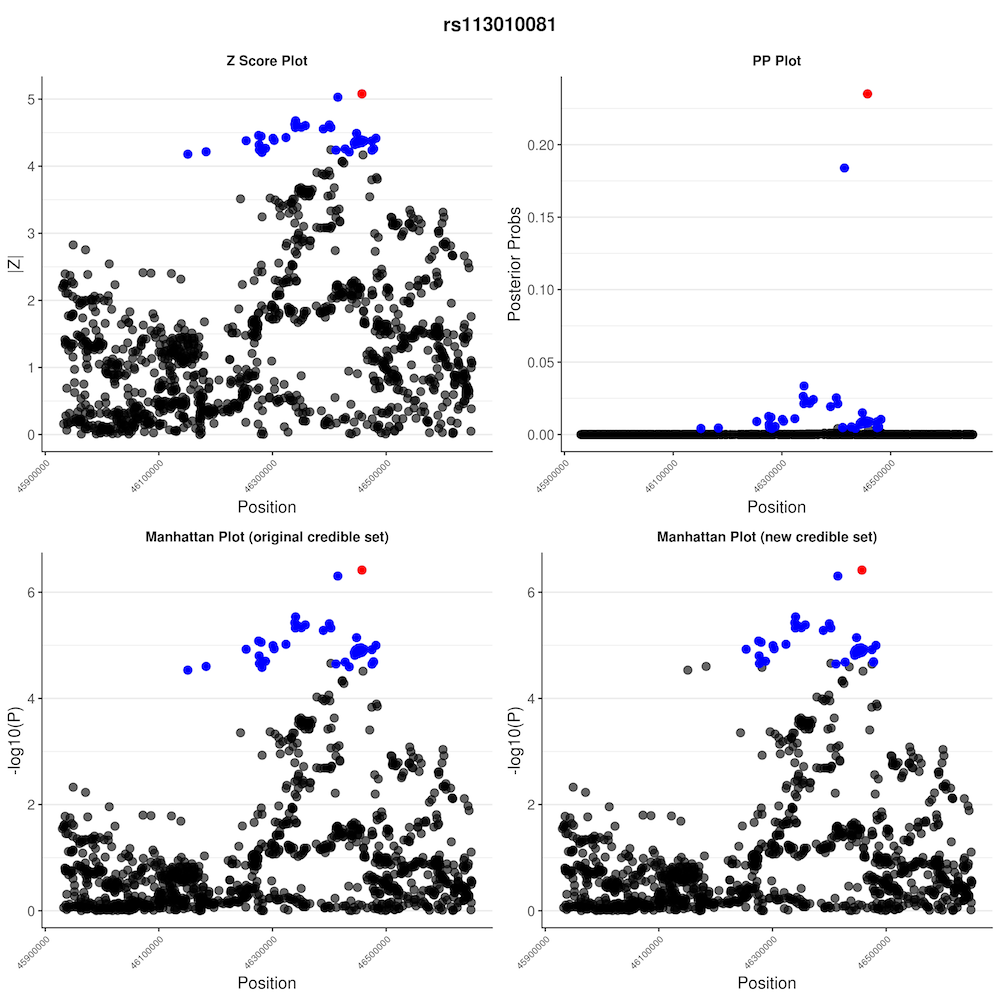

Supplement: S1 File — Zip file containing Z-score plots, PP plots and Manhattan plots for the 39 T1D association regions analysed. (ZIP) [file pcbi.1007829.s014.zip › S1_file/rs113010081.png]

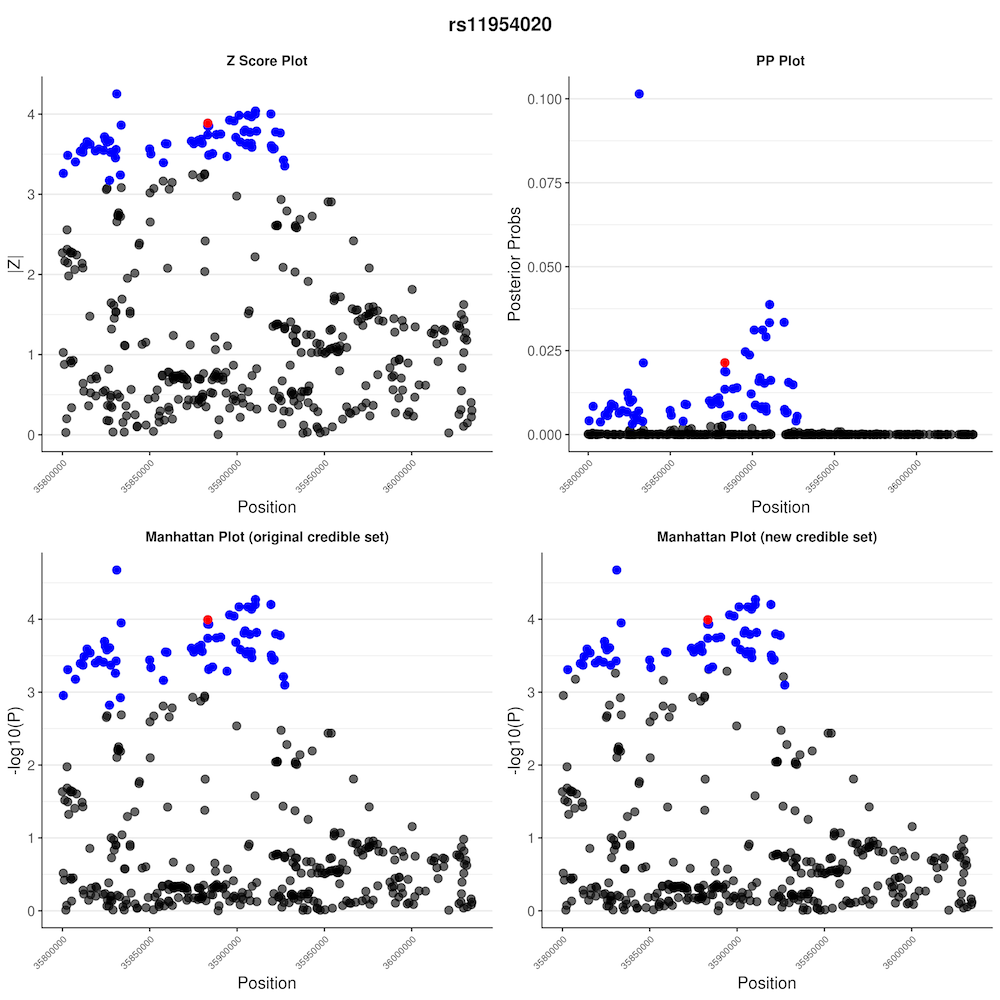

Supplement: S1 File — Zip file containing Z-score plots, PP plots and Manhattan plots for the 39 T1D association regions analysed. (ZIP) [file pcbi.1007829.s014.zip › S1_file/rs11954020.png]

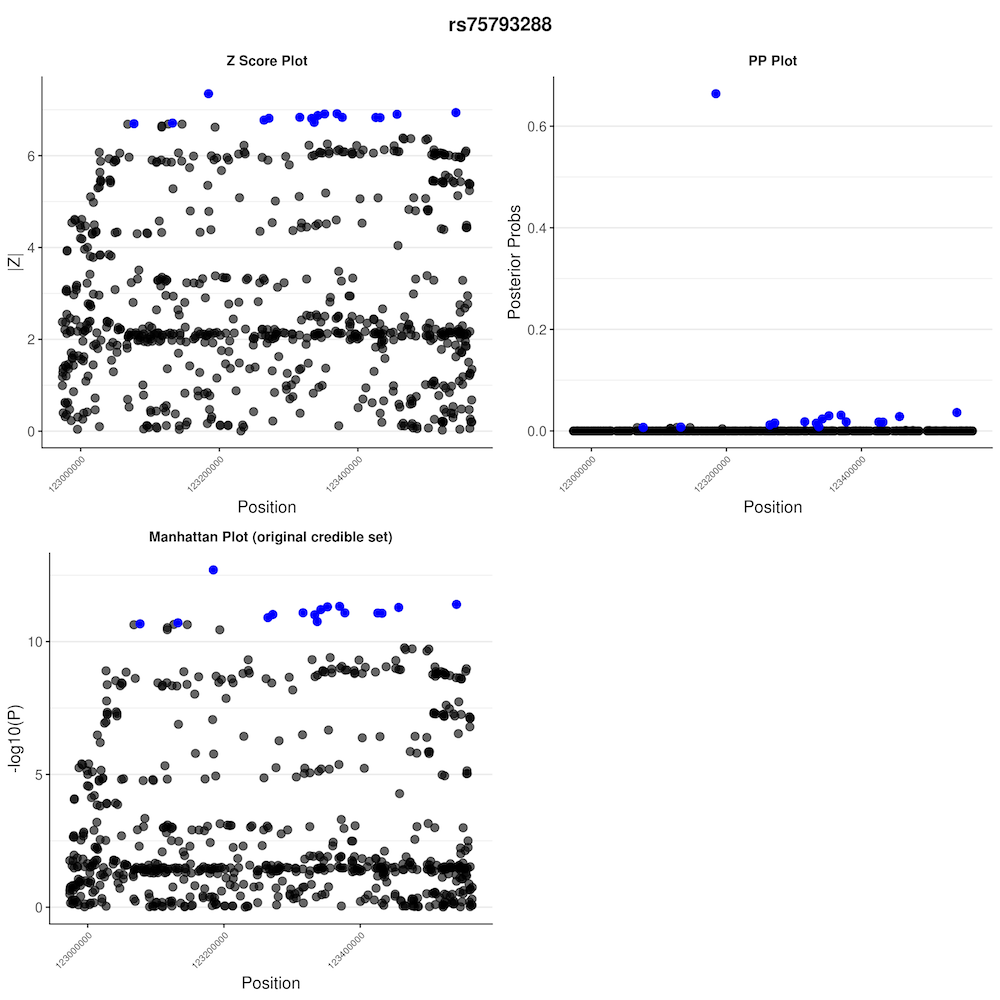

Supplement: S1 File — Zip file containing Z-score plots, PP plots and Manhattan plots for the 39 T1D association regions analysed. (ZIP) [file pcbi.1007829.s014.zip › S1_file/rs75793288.png]

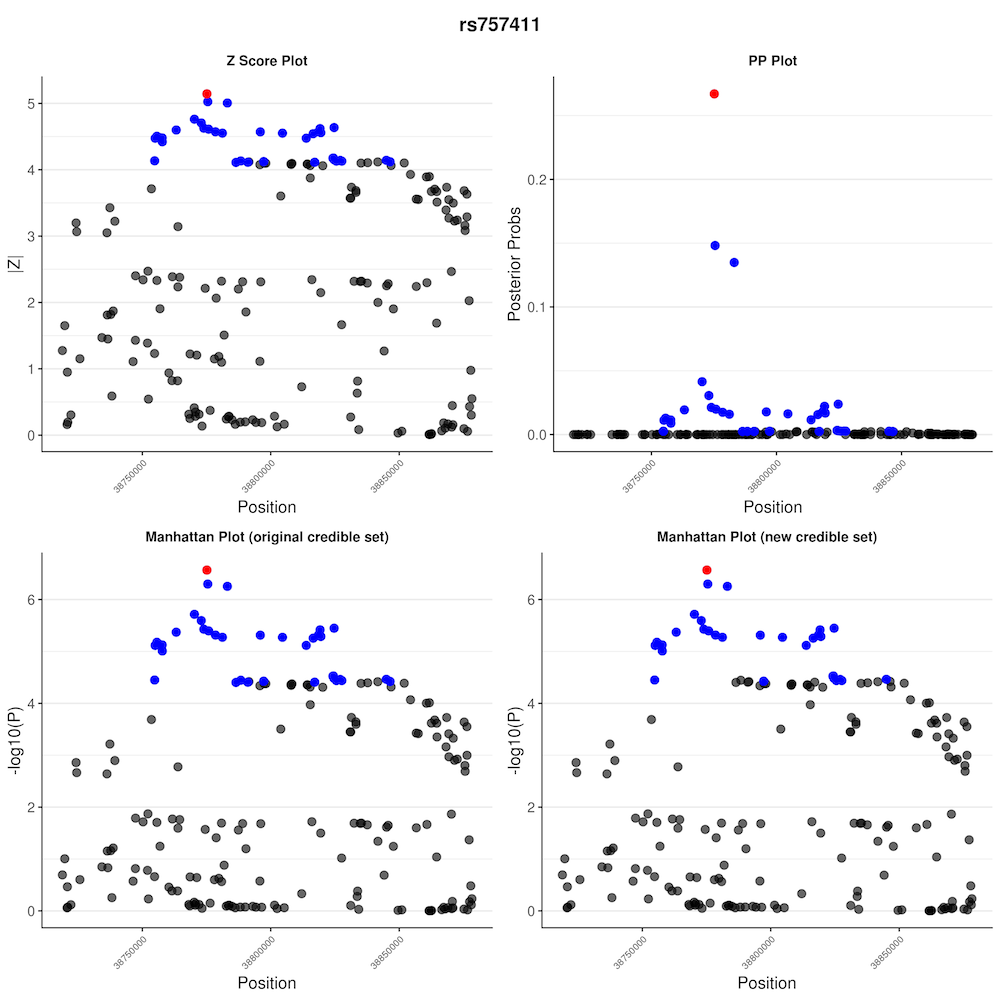

Supplement: S1 File — Zip file containing Z-score plots, PP plots and Manhattan plots for the 39 T1D association regions analysed. (ZIP) [file pcbi.1007829.s014.zip › S1_file/rs757411.png]

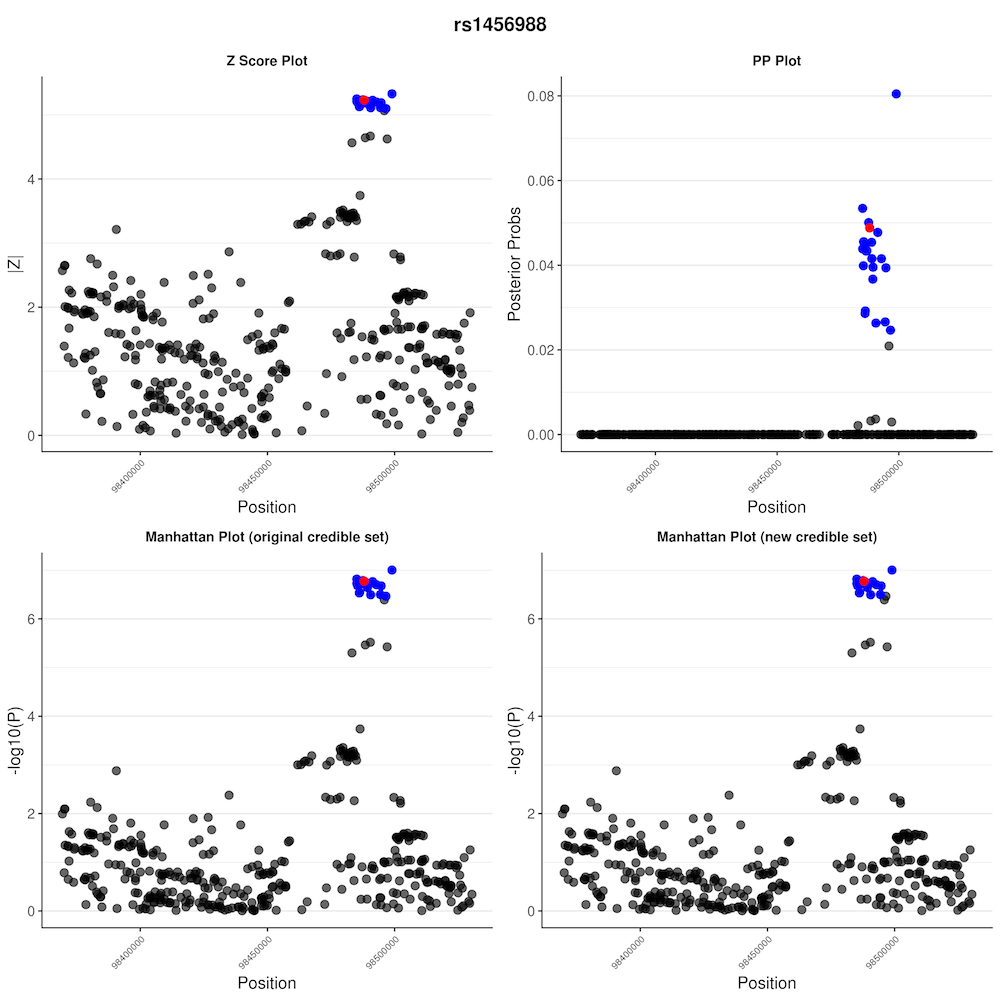

Supplement: S1 File — Zip file containing Z-score plots, PP plots and Manhattan plots for the 39 T1D association regions analysed. (ZIP) [file pcbi.1007829.s014.zip › S1_file/rs1456988.png]

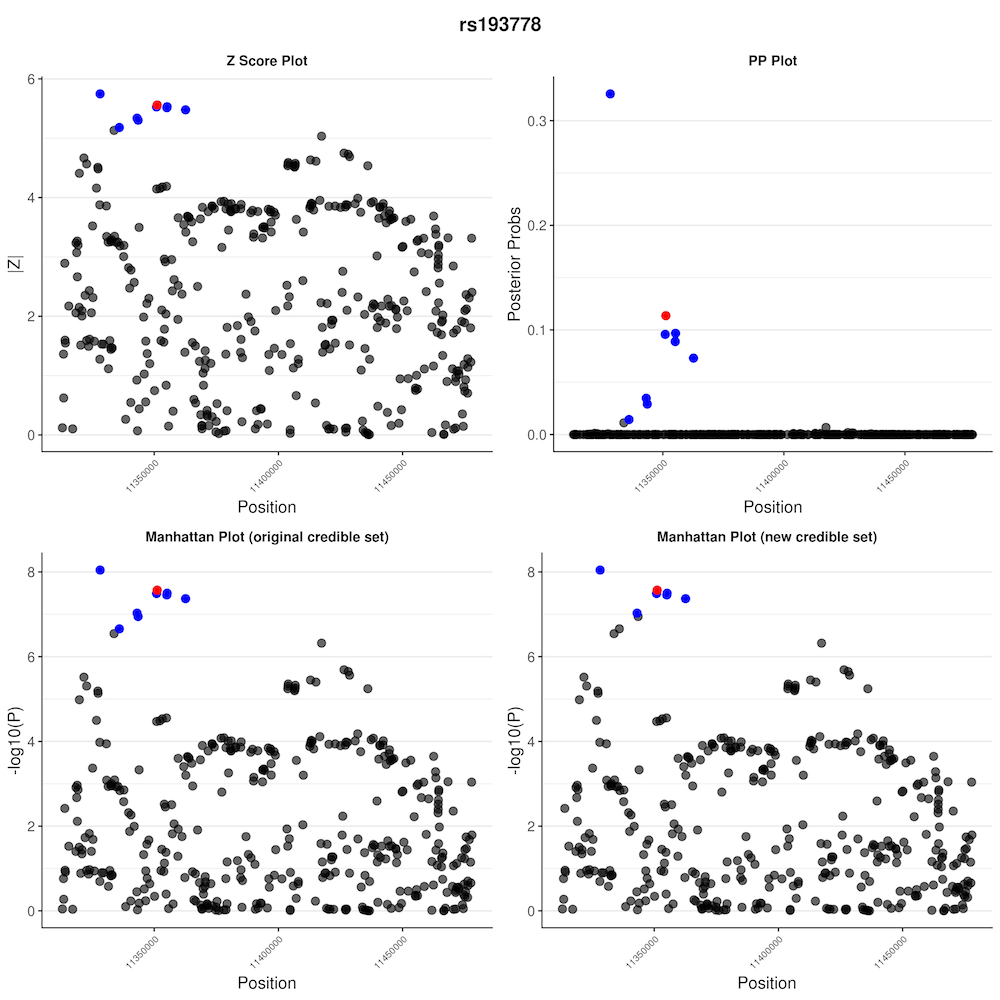

Supplement: S1 File — Zip file containing Z-score plots, PP plots and Manhattan plots for the 39 T1D association regions analysed. (ZIP) [file pcbi.1007829.s014.zip › S1_file/rs193778.png]

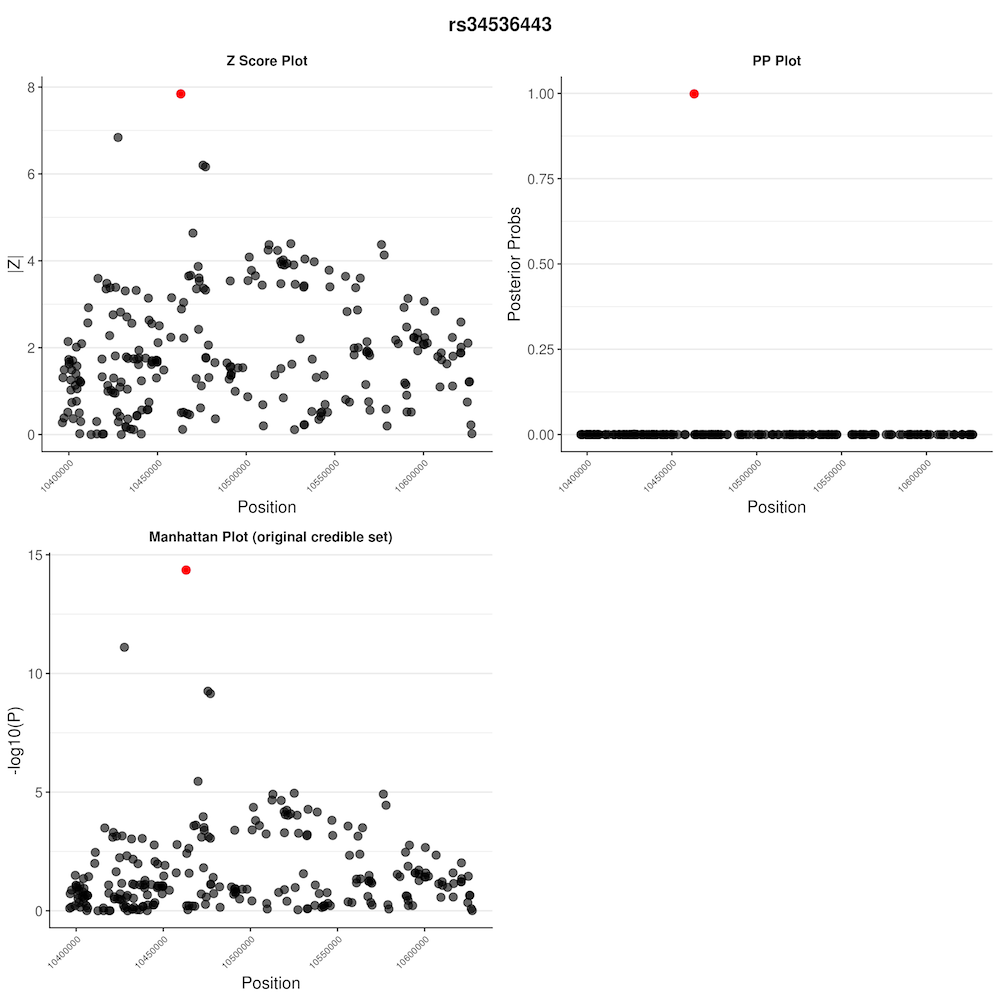

Supplement: S1 File — Zip file containing Z-score plots, PP plots and Manhattan plots for the 39 T1D association regions analysed. (ZIP) [file pcbi.1007829.s014.zip › S1_file/rs34536443.png]

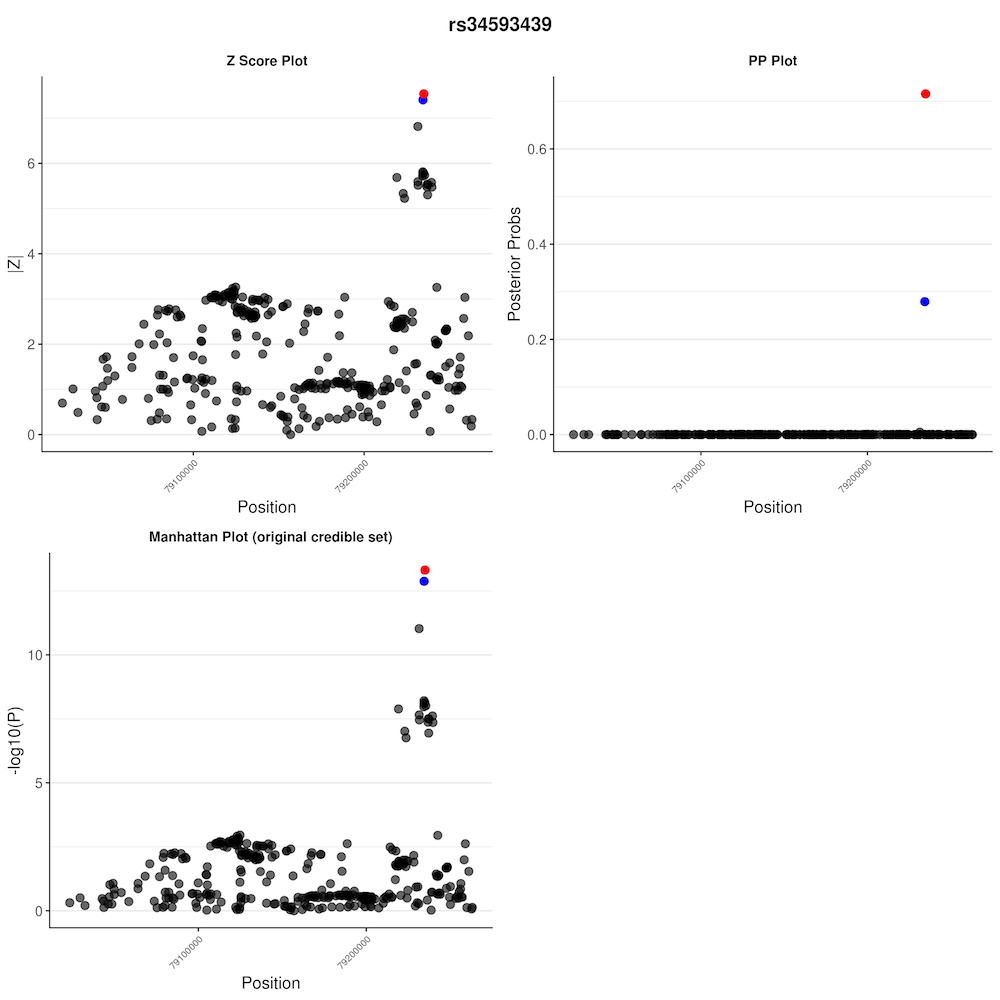

Supplement: S1 File — Zip file containing Z-score plots, PP plots and Manhattan plots for the 39 T1D association regions analysed. (ZIP) [file pcbi.1007829.s014.zip › S1_file/rs34593439.png]

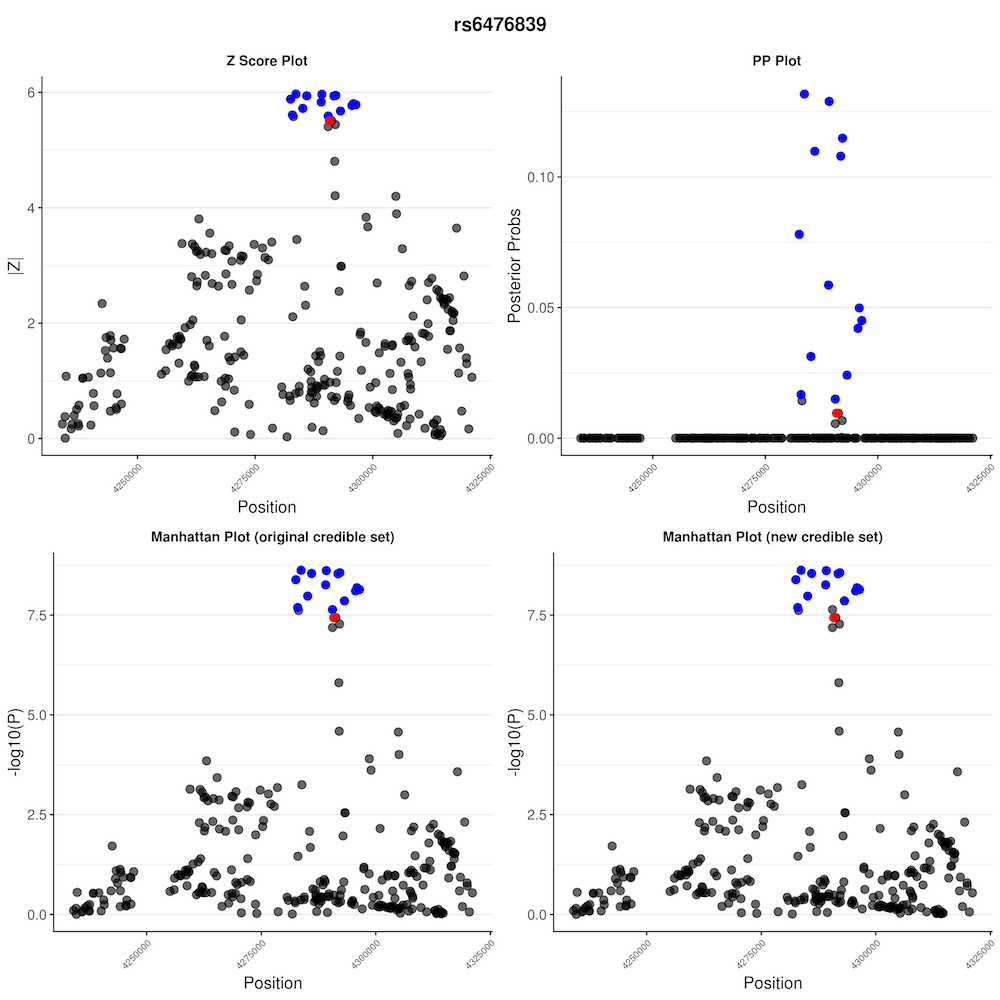

Supplement: S1 File — Zip file containing Z-score plots, PP plots and Manhattan plots for the 39 T1D association regions analysed. (ZIP) [file pcbi.1007829.s014.zip › S1_file/rs6476839.png]

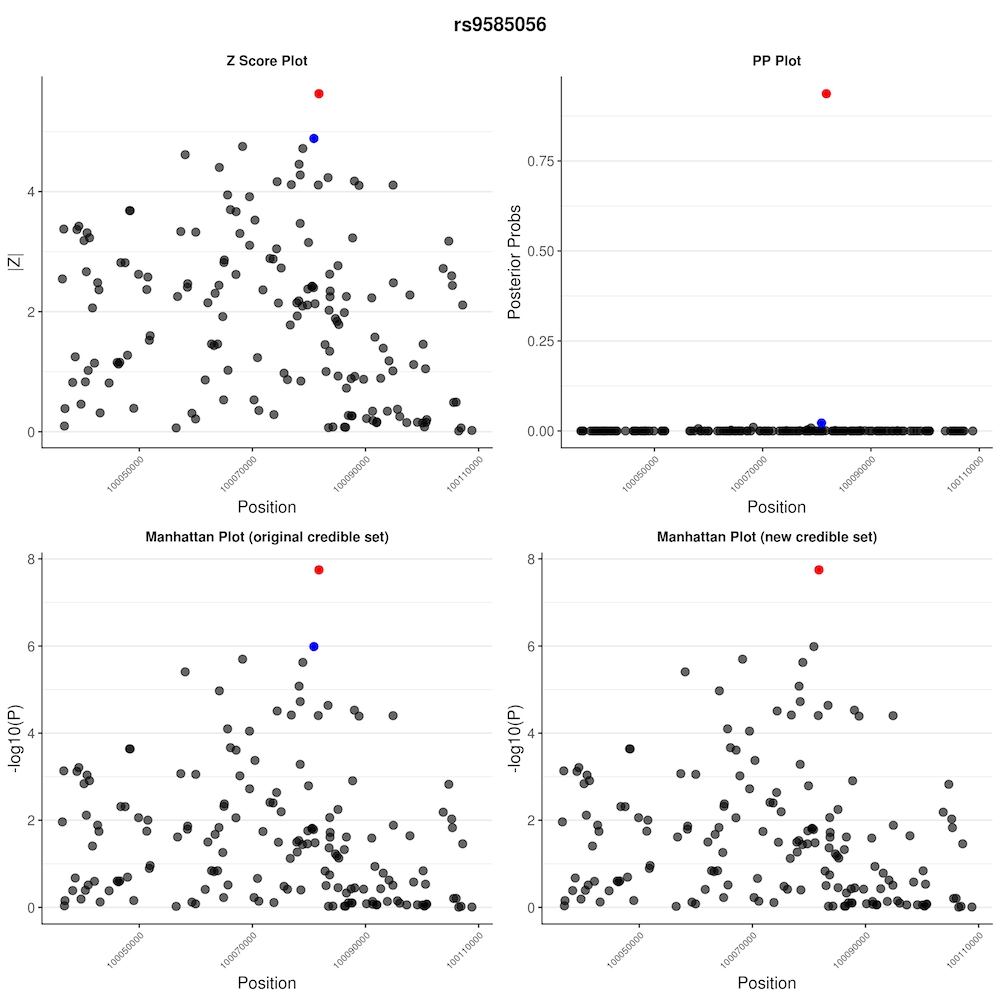

Supplement: S1 File — Zip file containing Z-score plots, PP plots and Manhattan plots for the 39 T1D association regions analysed. (ZIP) [file pcbi.1007829.s014.zip › S1_file/rs9585056.png]

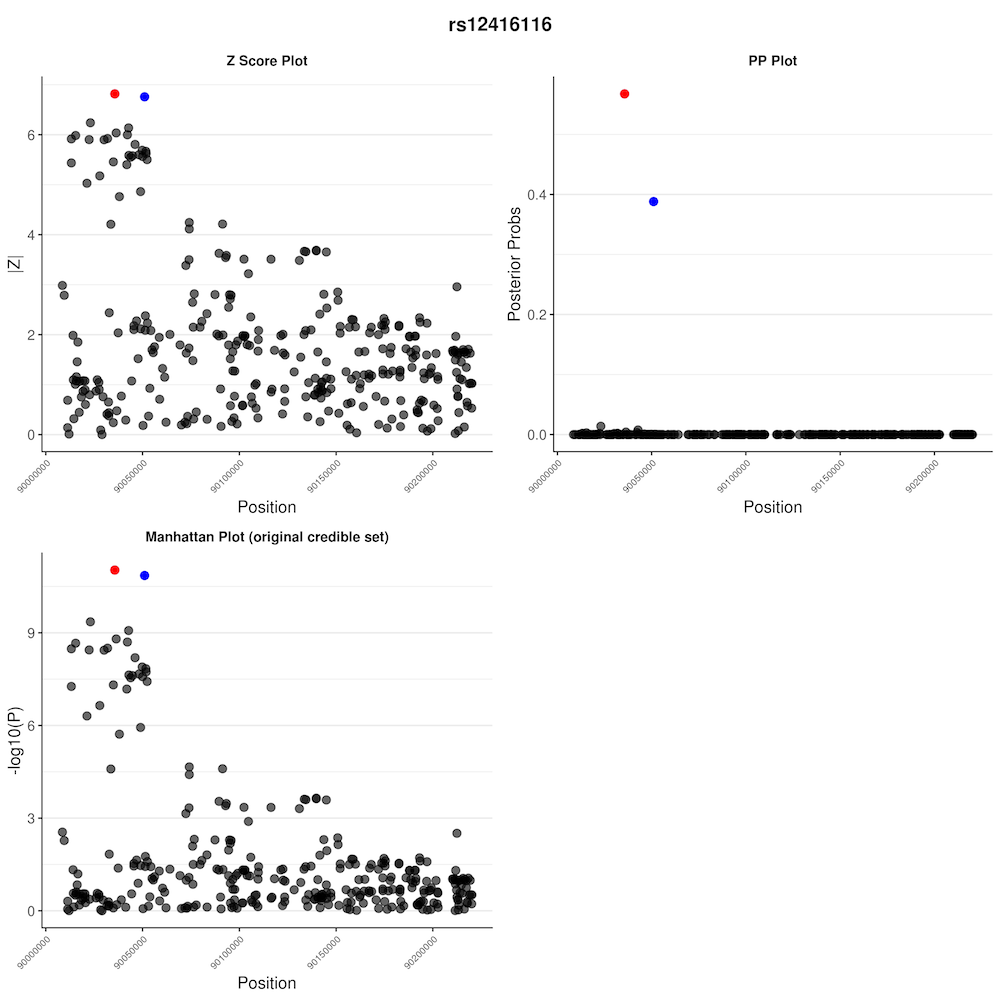

Supplement: S1 File — Zip file containing Z-score plots, PP plots and Manhattan plots for the 39 T1D association regions analysed. (ZIP) [file pcbi.1007829.s014.zip › S1_file/rs12416116.png]

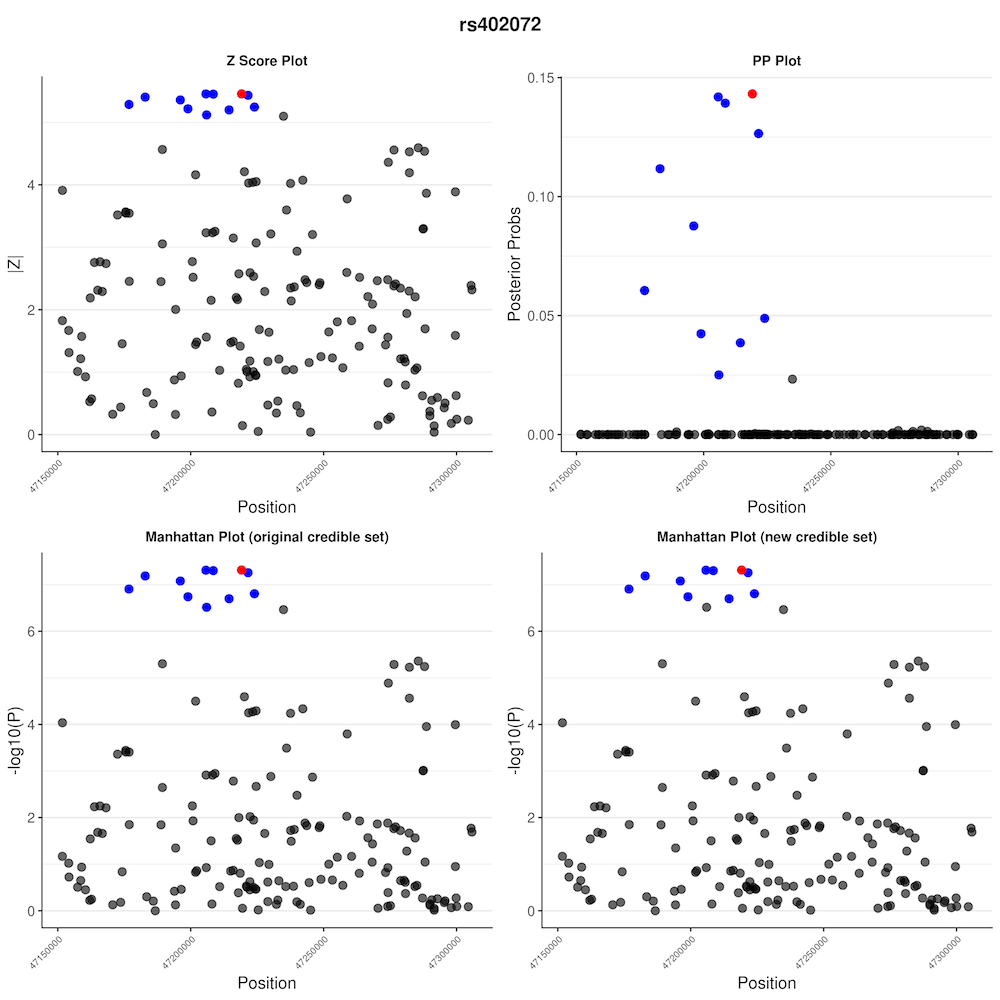

Supplement: S1 File — Zip file containing Z-score plots, PP plots and Manhattan plots for the 39 T1D association regions analysed. (ZIP) [file pcbi.1007829.s014.zip › S1_file/rs402072.png]

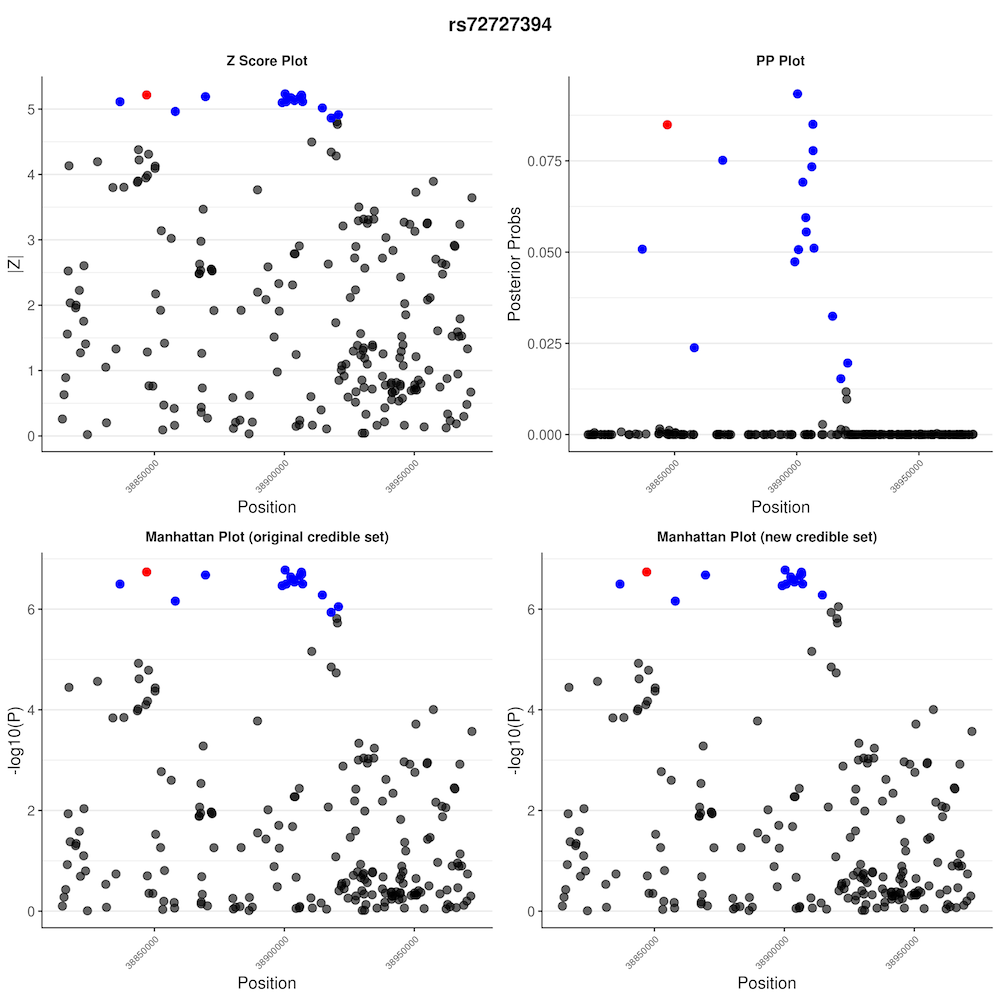

Supplement: S1 File — Zip file containing Z-score plots, PP plots and Manhattan plots for the 39 T1D association regions analysed. (ZIP) [file pcbi.1007829.s014.zip › S1_file/rs72727394.png]

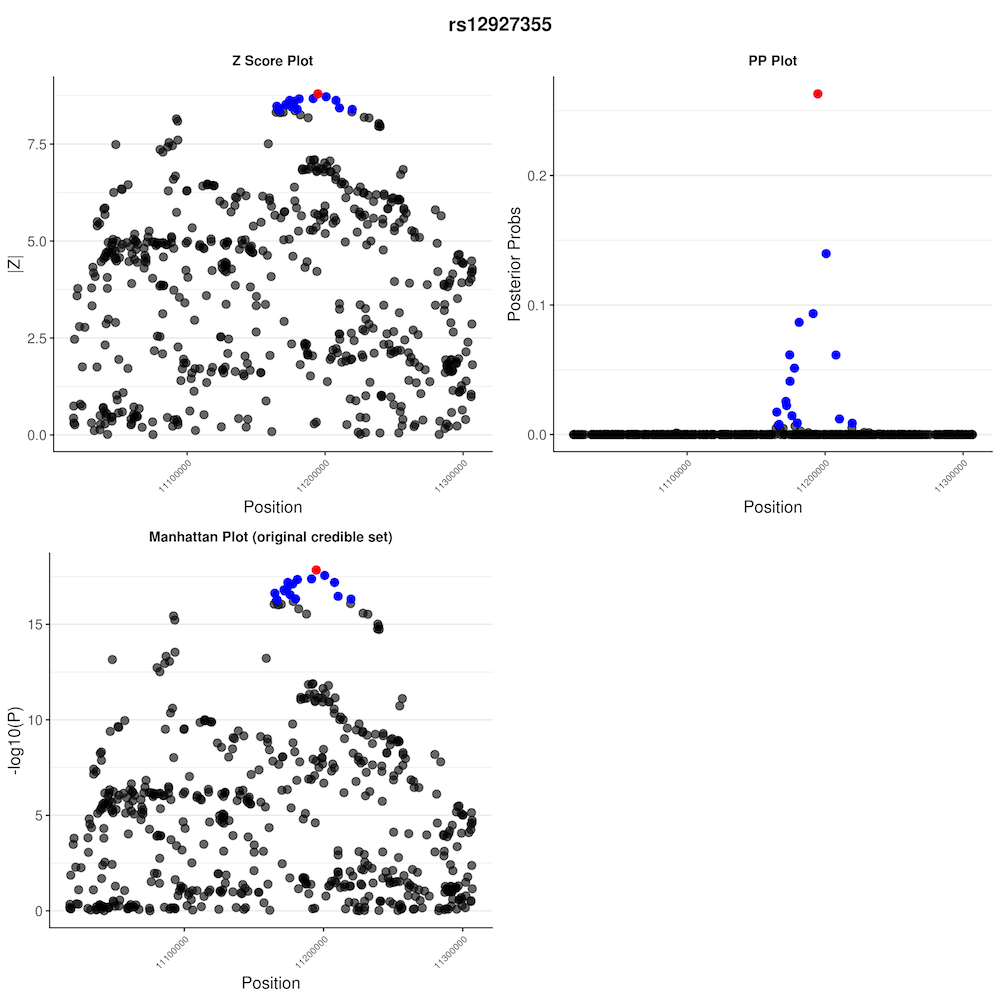

Supplement: S1 File — Zip file containing Z-score plots, PP plots and Manhattan plots for the 39 T1D association regions analysed. (ZIP) [file pcbi.1007829.s014.zip › S1_file/rs12927355.png]

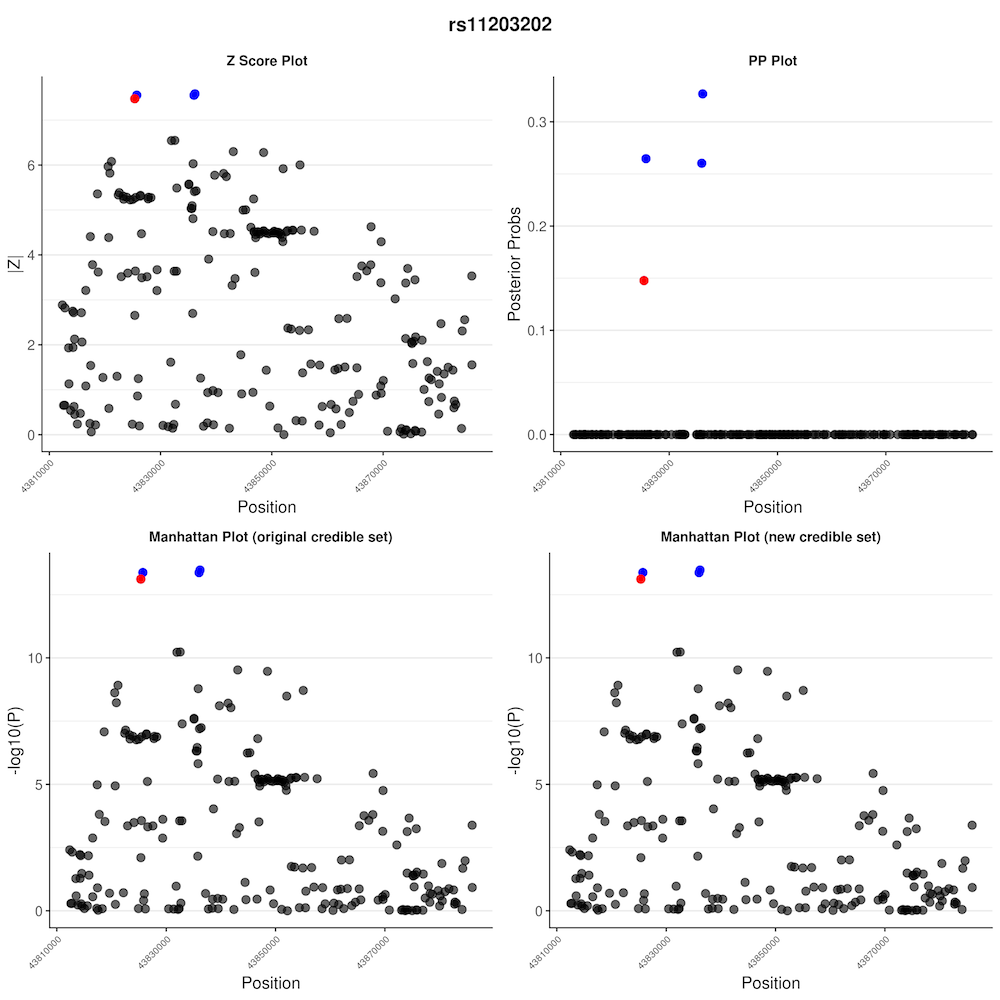

Supplement: S1 File — Zip file containing Z-score plots, PP plots and Manhattan plots for the 39 T1D association regions analysed. (ZIP) [file pcbi.1007829.s014.zip › S1_file/rs11203202.png]

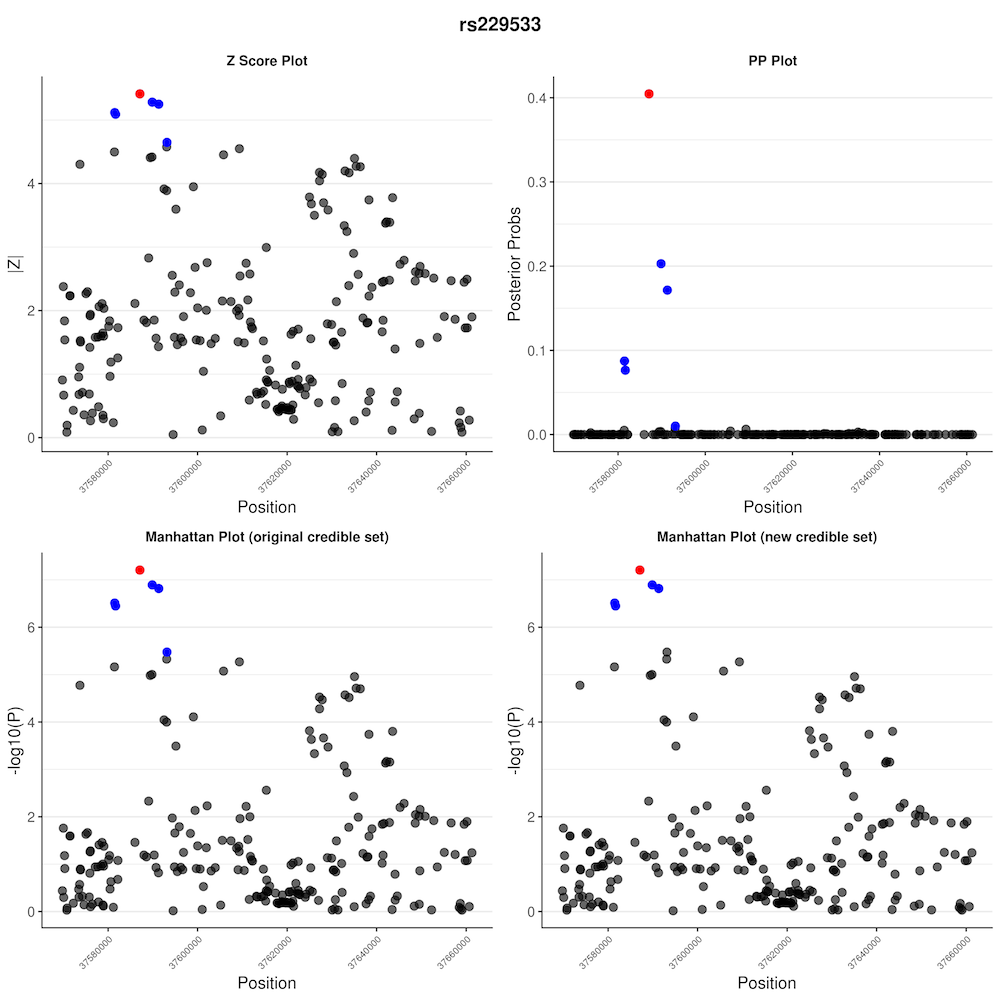

Supplement: S1 File — Zip file containing Z-score plots, PP plots and Manhattan plots for the 39 T1D association regions analysed. (ZIP) [file pcbi.1007829.s014.zip › S1_file/rs229533.png]

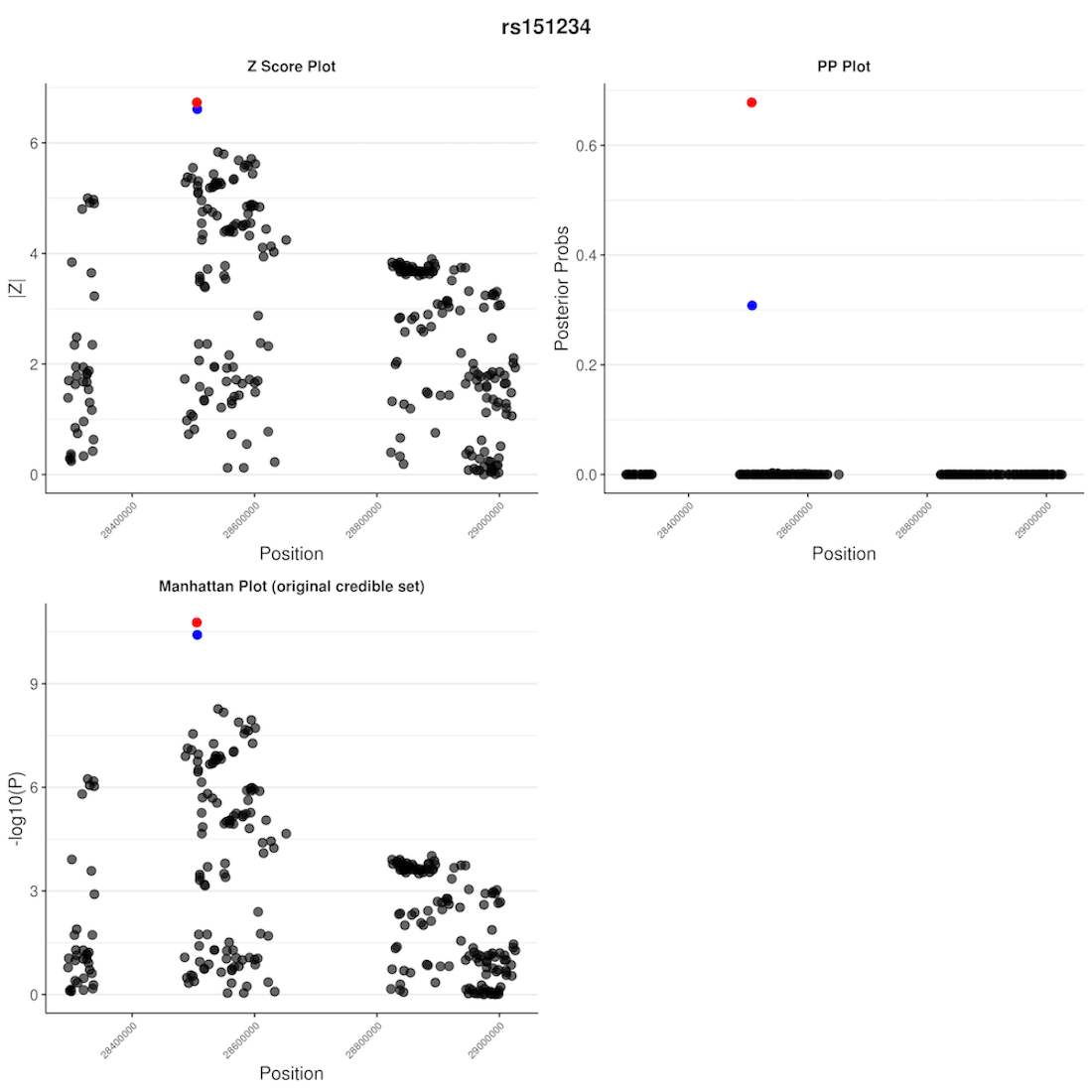

Supplement: S1 File — Zip file containing Z-score plots, PP plots and Manhattan plots for the 39 T1D association regions analysed. (ZIP) [file pcbi.1007829.s014.zip › S1_file/rs151234.png]

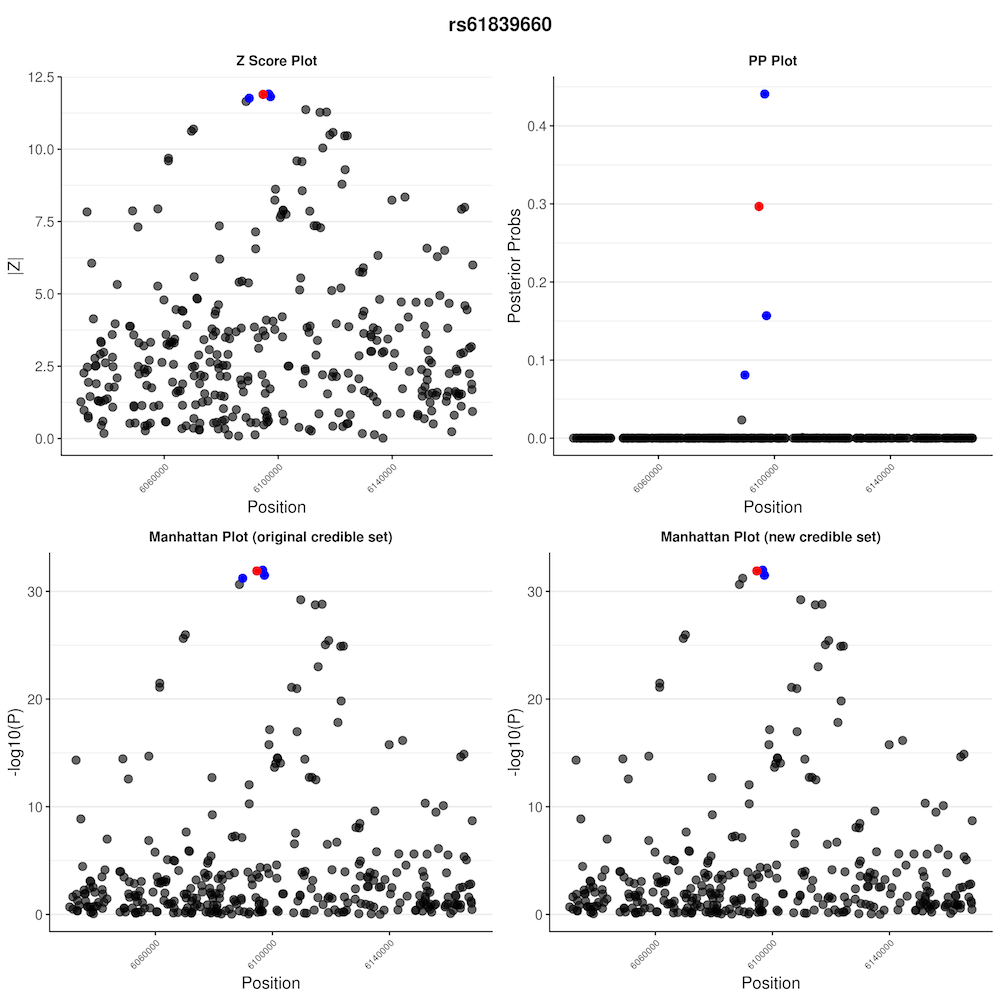

Supplement: S1 File — Zip file containing Z-score plots, PP plots and Manhattan plots for the 39 T1D association regions analysed. (ZIP) [file pcbi.1007829.s014.zip › S1_file/rs61839660.png]

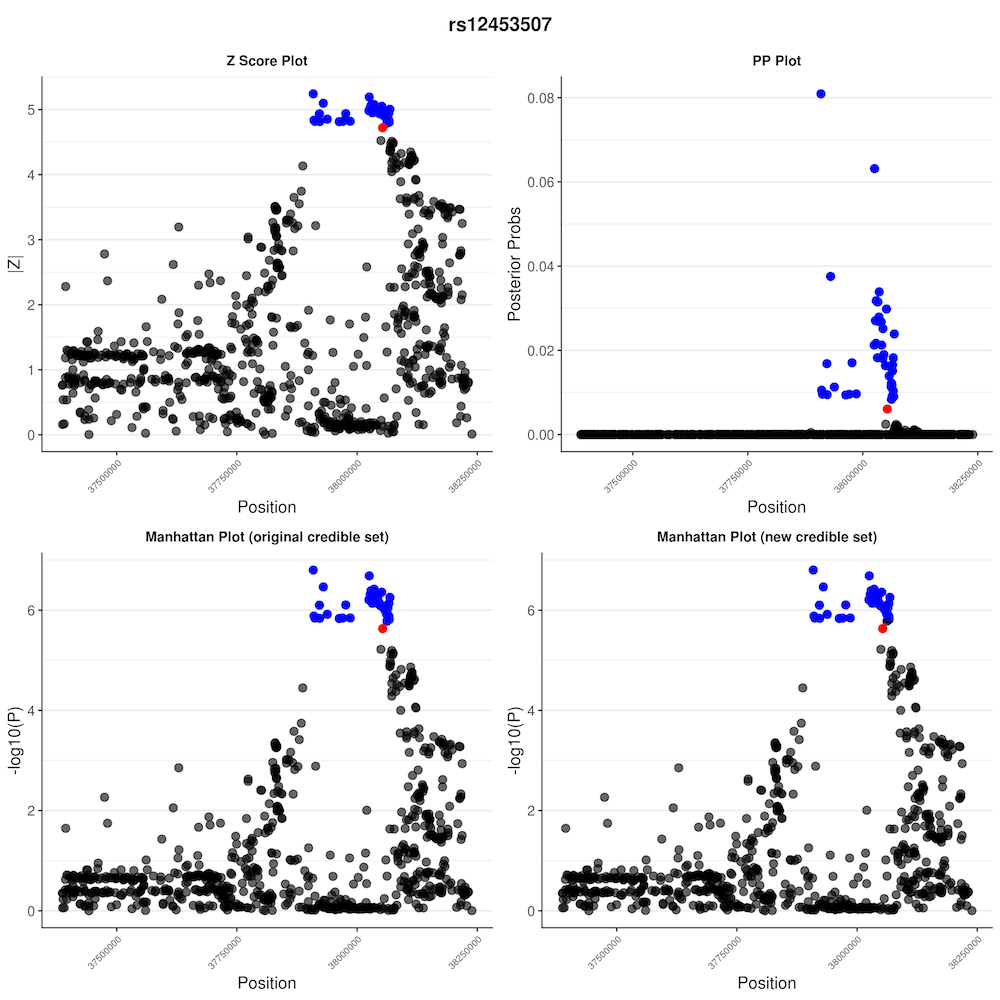

Supplement: S1 File — Zip file containing Z-score plots, PP plots and Manhattan plots for the 39 T1D association regions analysed. (ZIP) [file pcbi.1007829.s014.zip › S1_file/rs12453507.png]

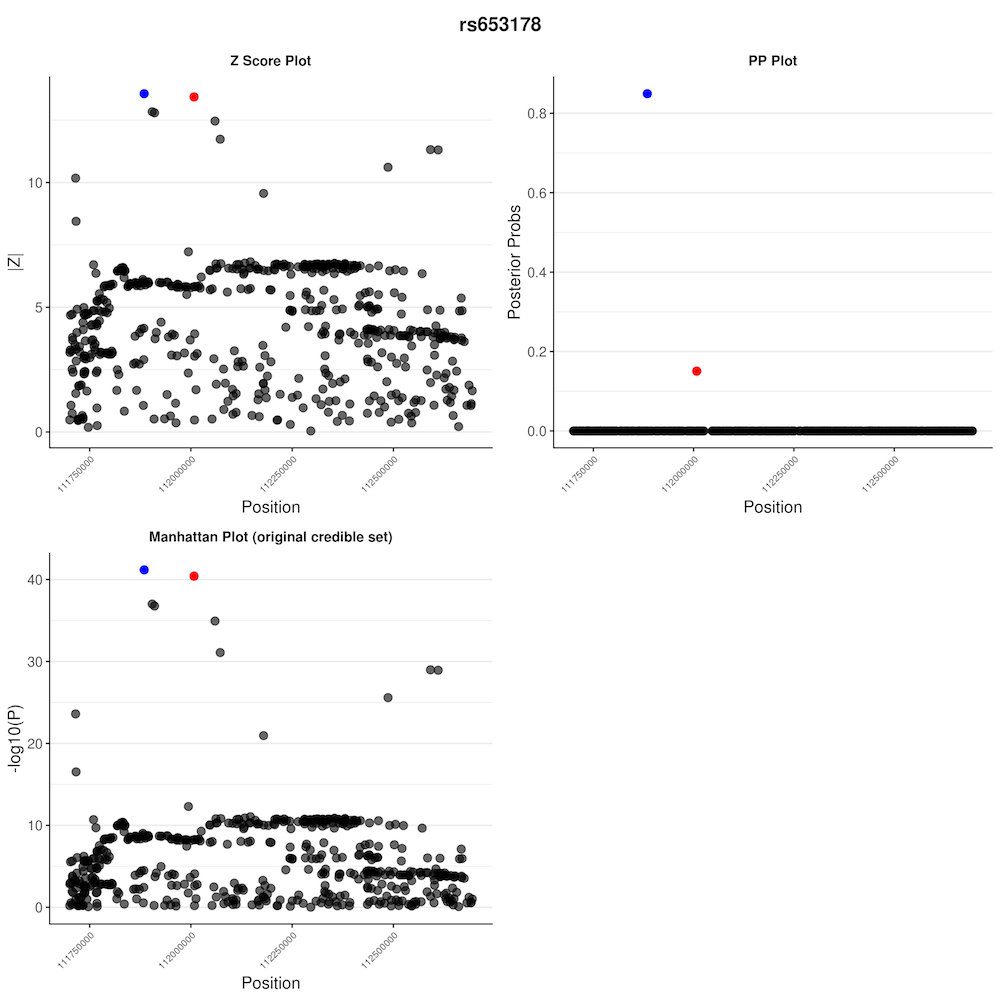

Supplement: S1 File — Zip file containing Z-score plots, PP plots and Manhattan plots for the 39 T1D association regions analysed. (ZIP) [file pcbi.1007829.s014.zip › S1_file/rs653178.png]

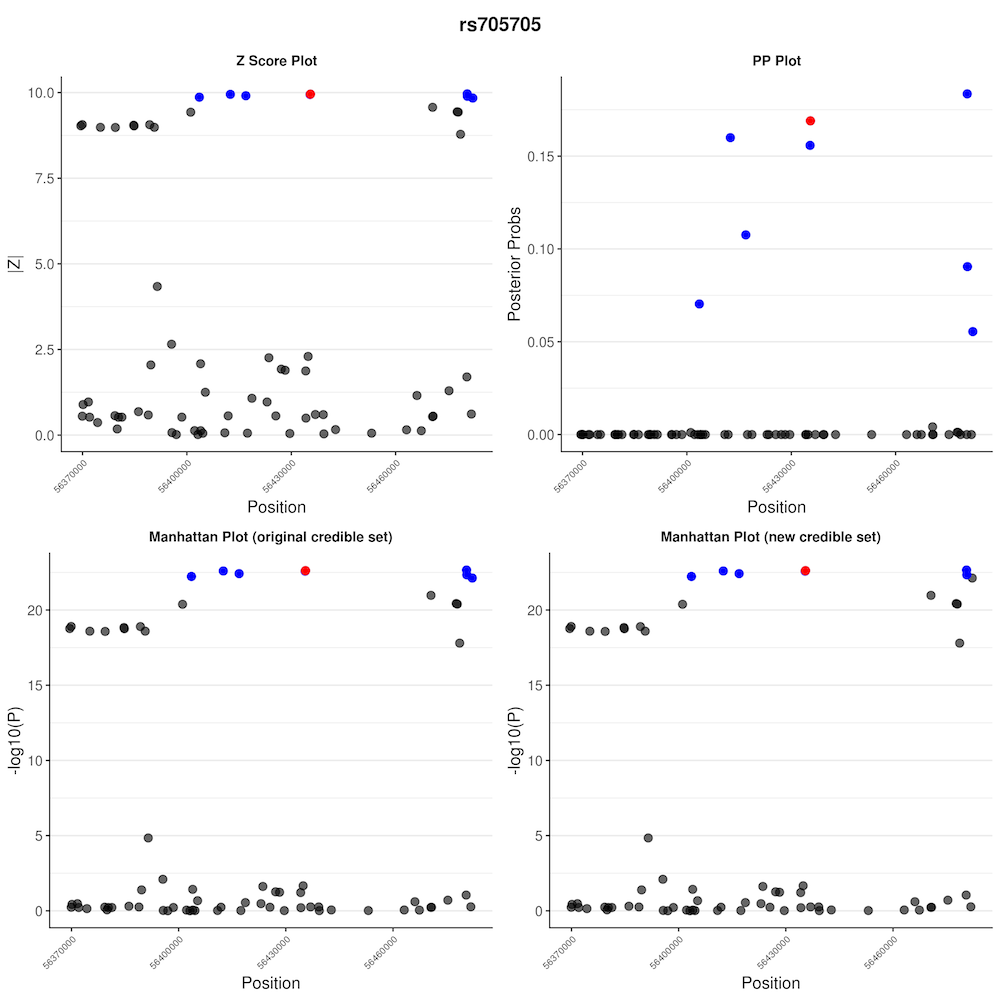

Supplement: S1 File — Zip file containing Z-score plots, PP plots and Manhattan plots for the 39 T1D association regions analysed. (ZIP) [file pcbi.1007829.s014.zip › S1_file/rs705705.png]

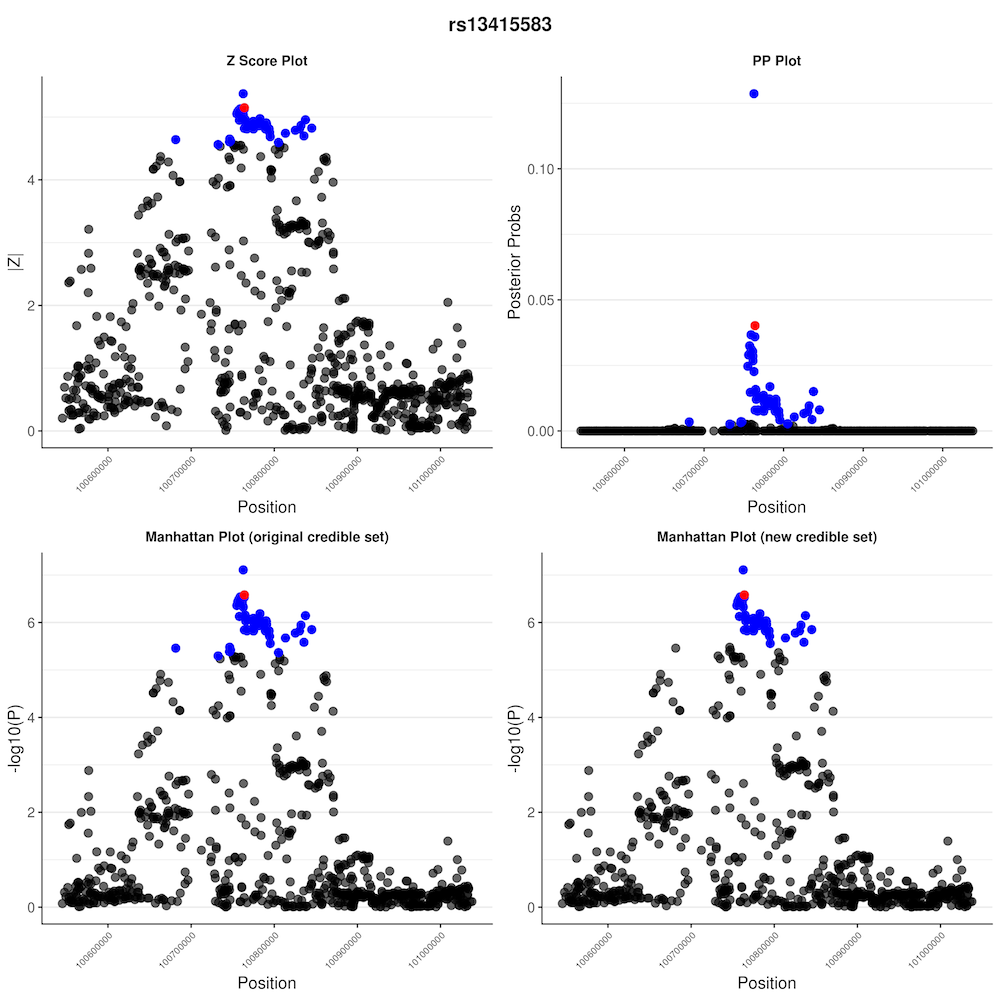

Supplement: S1 File — Zip file containing Z-score plots, PP plots and Manhattan plots for the 39 T1D association regions analysed. (ZIP) [file pcbi.1007829.s014.zip › S1_file/rs13415583.png]

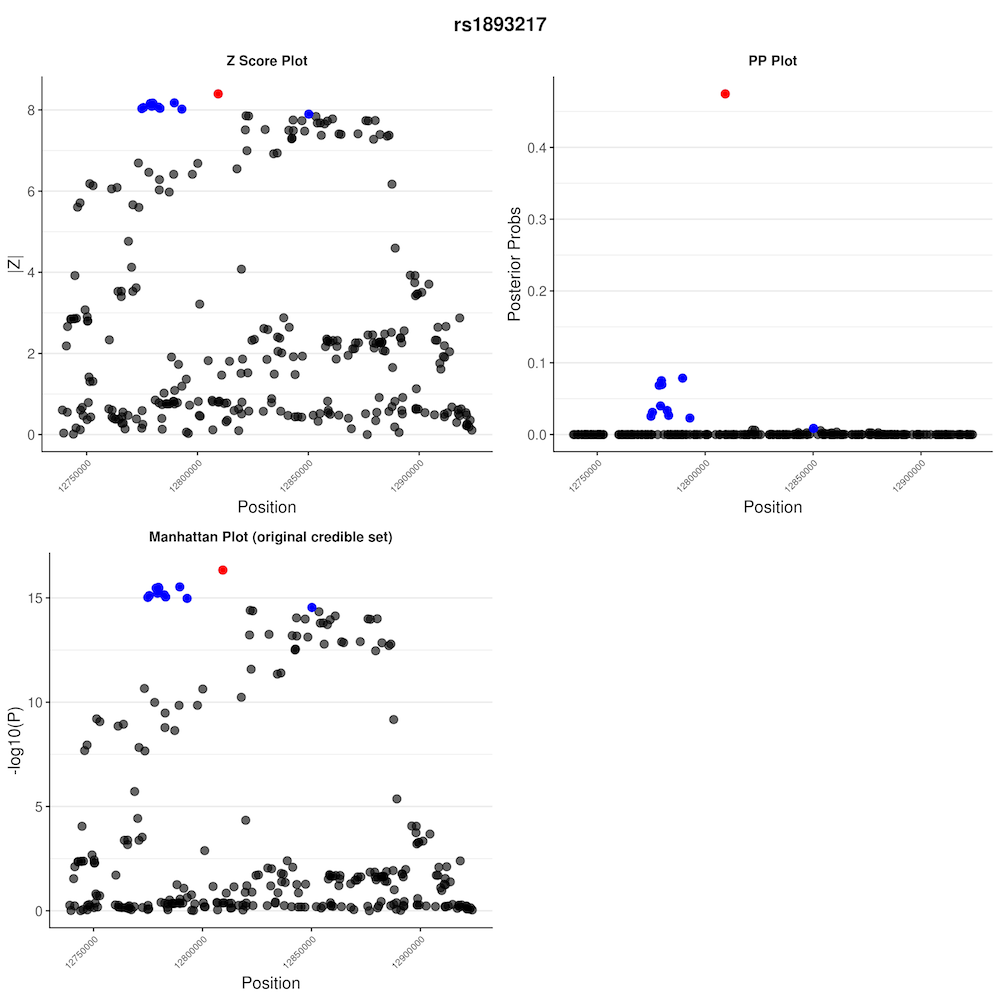

Supplement: S1 File — Zip file containing Z-score plots, PP plots and Manhattan plots for the 39 T1D association regions analysed. (ZIP) [file pcbi.1007829.s014.zip › S1_file/rs1893217.png]

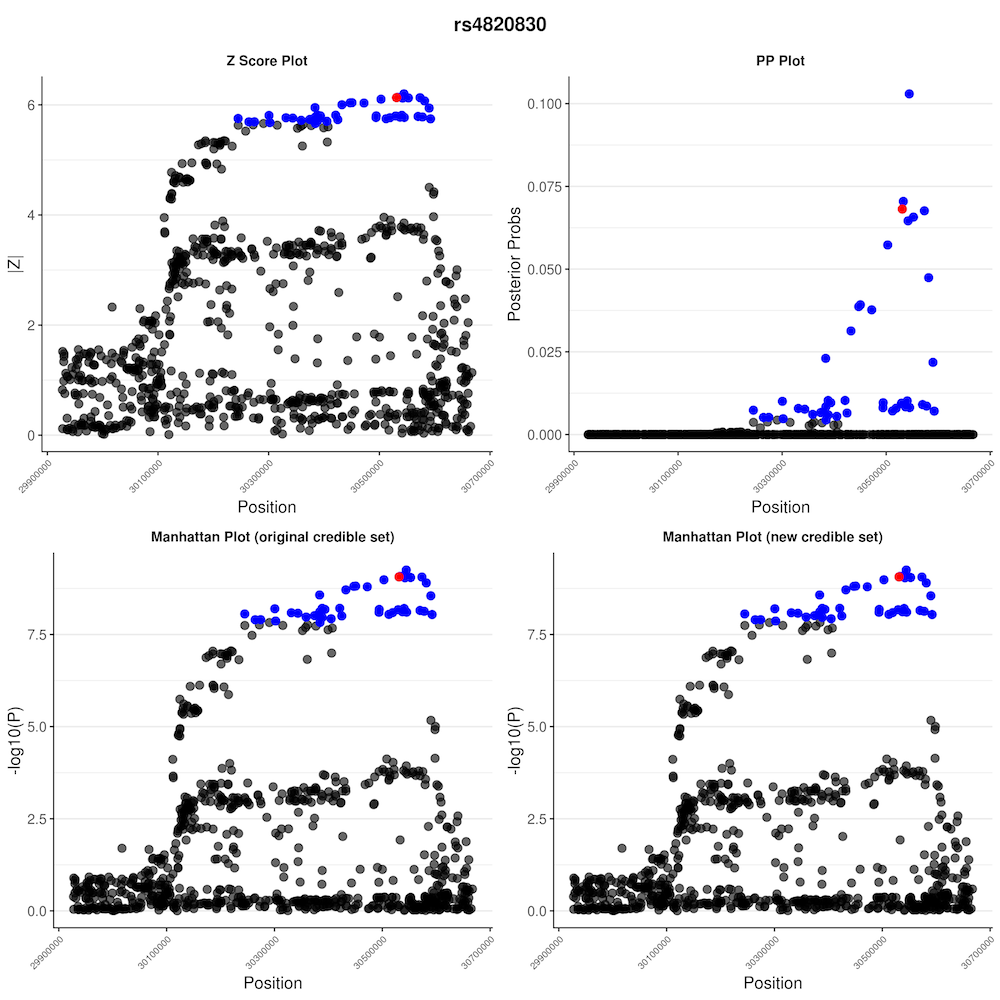

Supplement: S1 File — Zip file containing Z-score plots, PP plots and Manhattan plots for the 39 T1D association regions analysed. (ZIP) [file pcbi.1007829.s014.zip › S1_file/rs4820830.png]

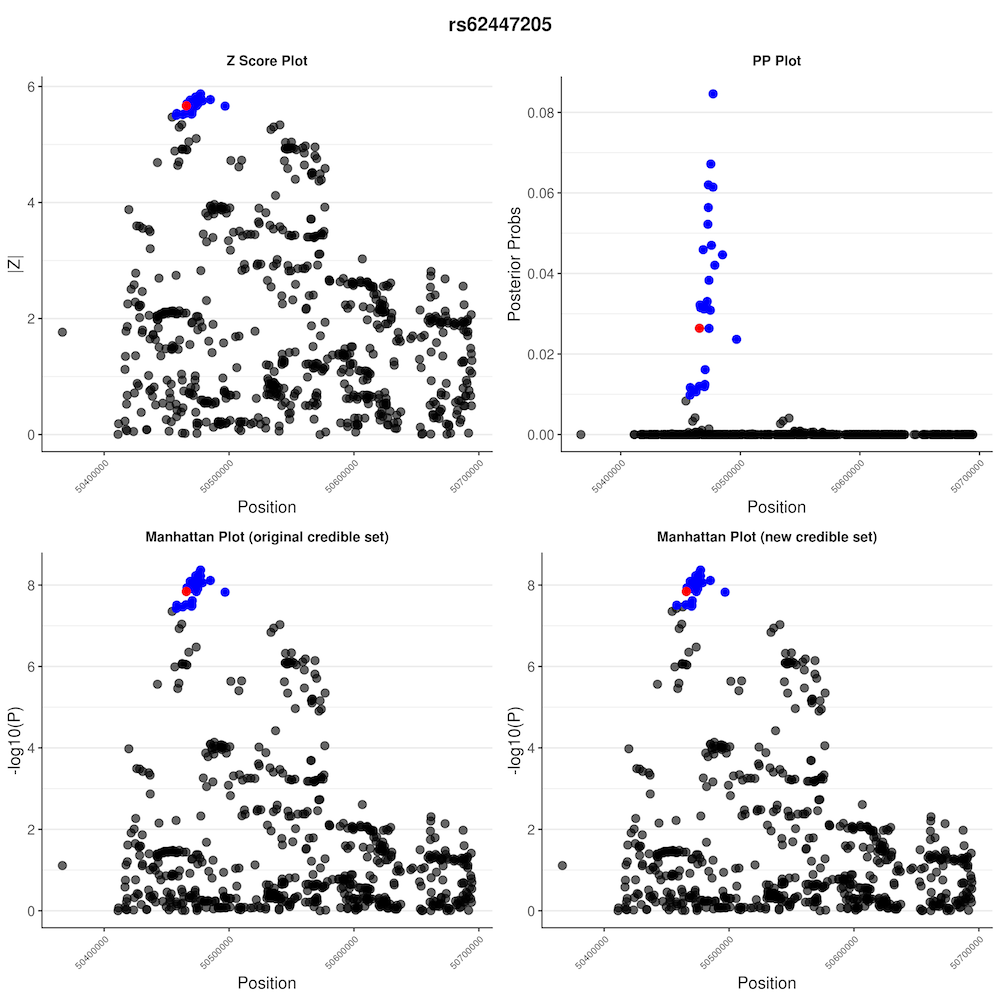

Supplement: S1 File — Zip file containing Z-score plots, PP plots and Manhattan plots for the 39 T1D association regions analysed. (ZIP) [file pcbi.1007829.s014.zip › S1_file/rs62447205.png]

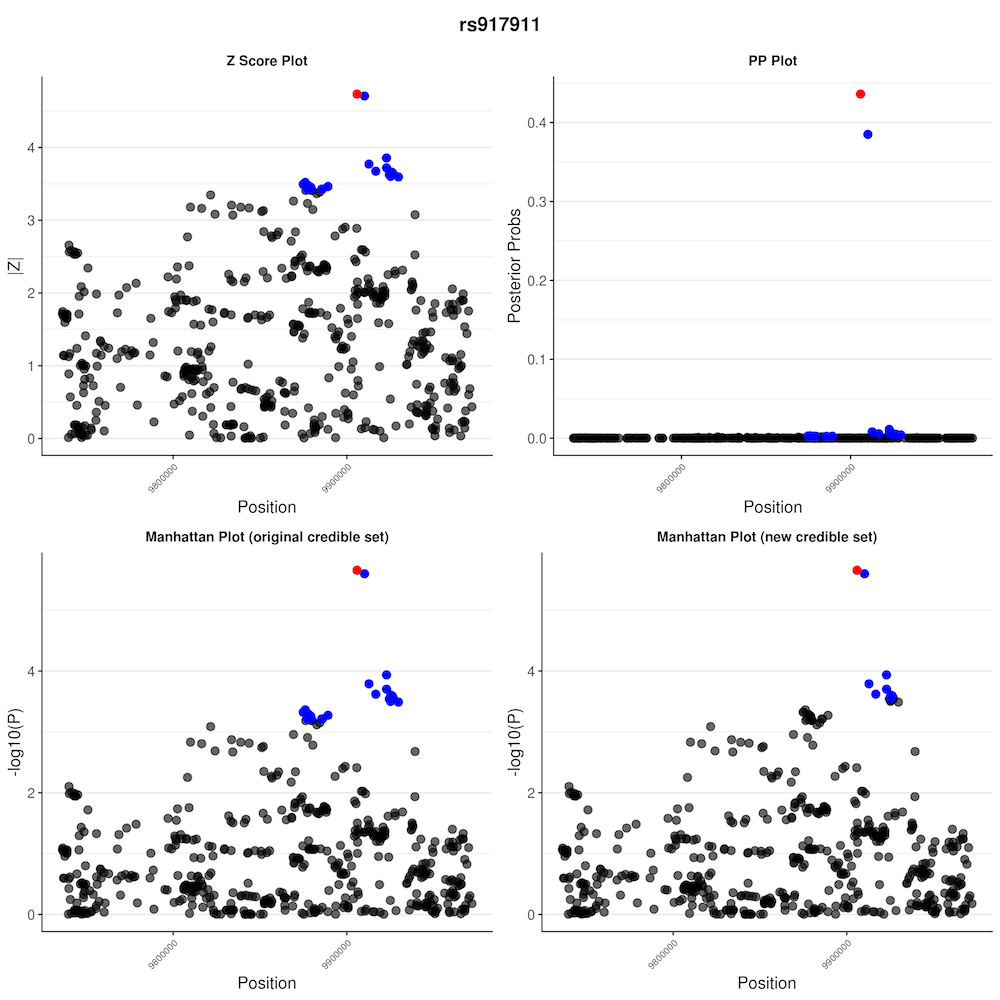

Supplement: S1 File — Zip file containing Z-score plots, PP plots and Manhattan plots for the 39 T1D association regions analysed. (ZIP) [file pcbi.1007829.s014.zip › S1_file/rs917911.png]

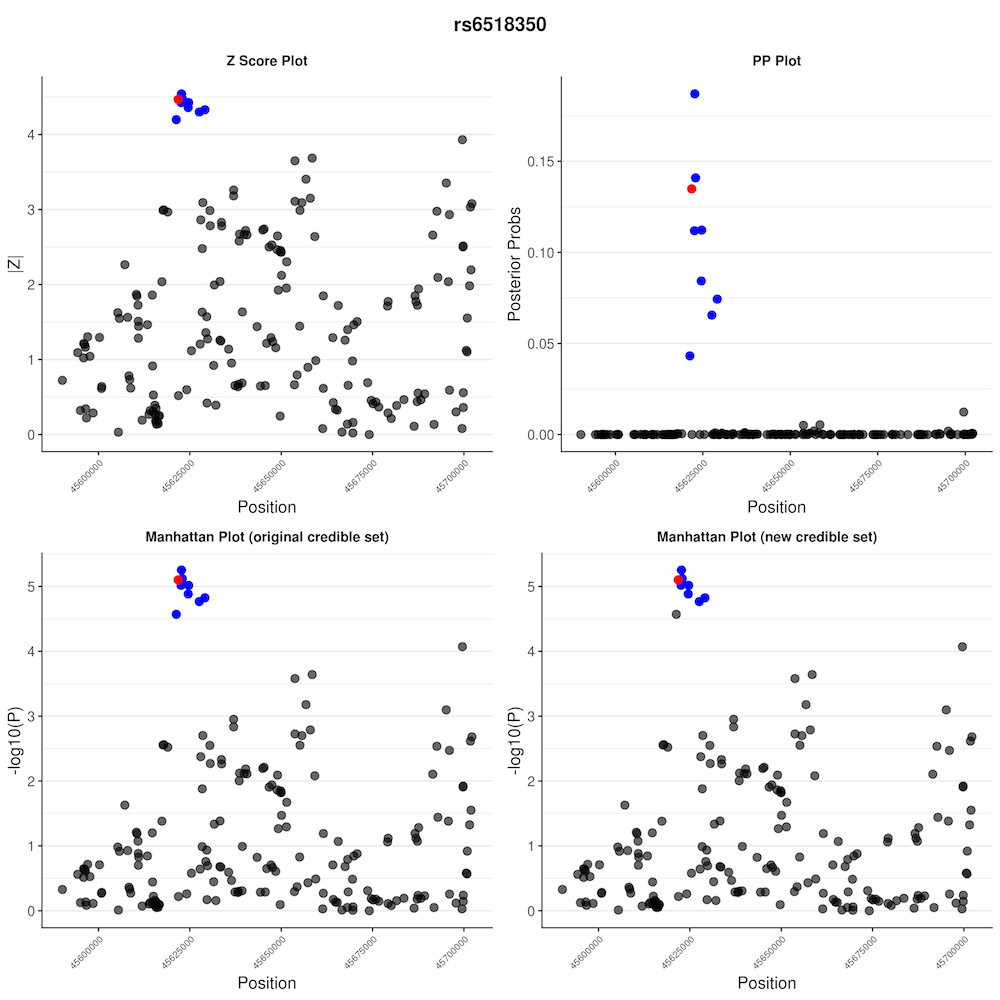

Supplement: S1 File — Zip file containing Z-score plots, PP plots and Manhattan plots for the 39 T1D association regions analysed. (ZIP) [file pcbi.1007829.s014.zip › S1_file/rs6518350.png]

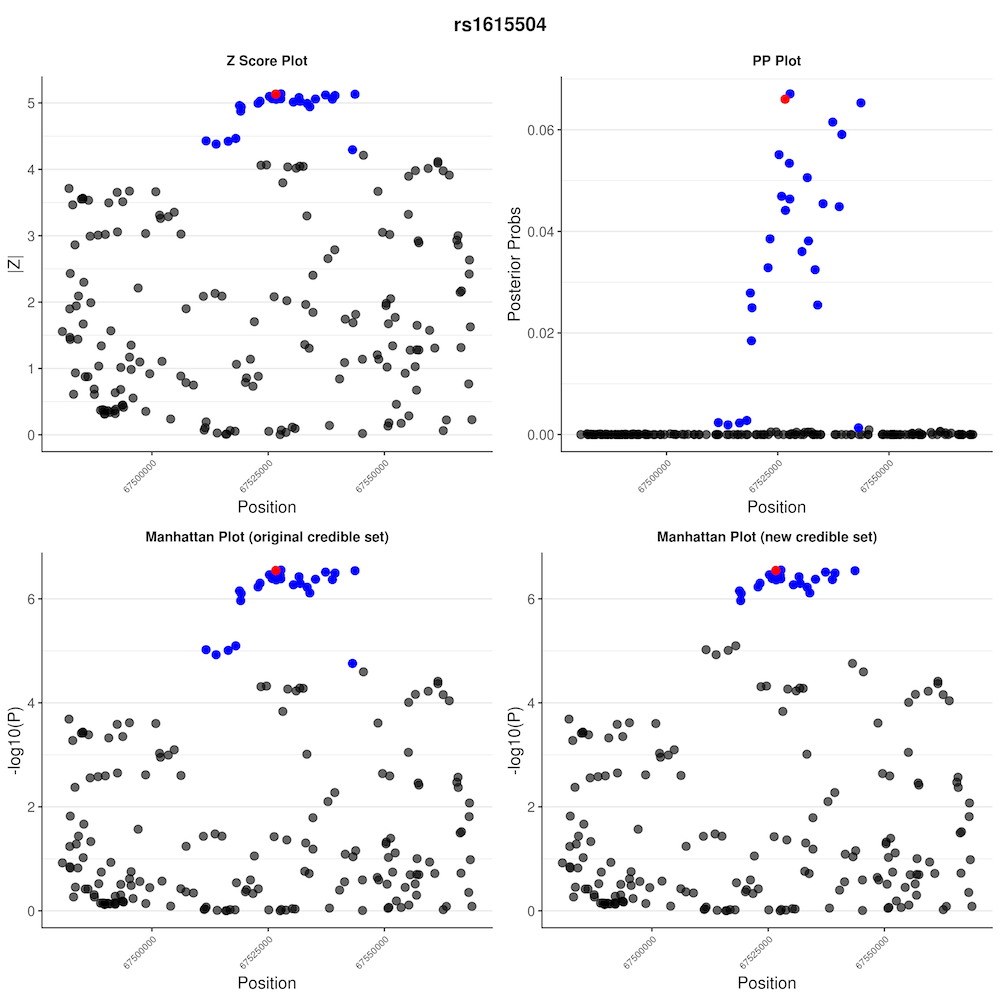

Supplement: S2 File — Zip file containing Z-score plots, PP plots and Manhattan plots for the 39 T1D association regions analysed. (ZIP) [file pcbi.1007829.s015.zip › S2_file/rs1615504.png]

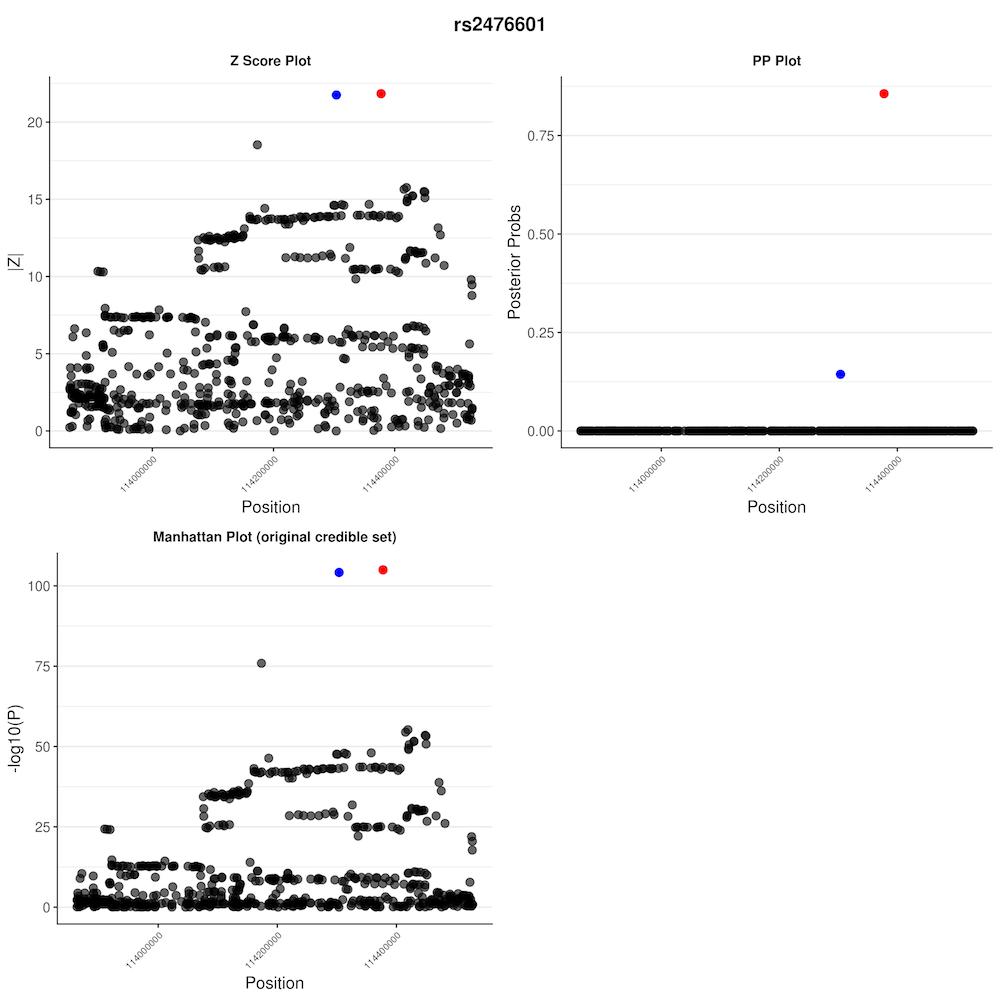

Supplement: S2 File — Zip file containing Z-score plots, PP plots and Manhattan plots for the 39 T1D association regions analysed. (ZIP) [file pcbi.1007829.s015.zip › S2_file/rs2476601.png]

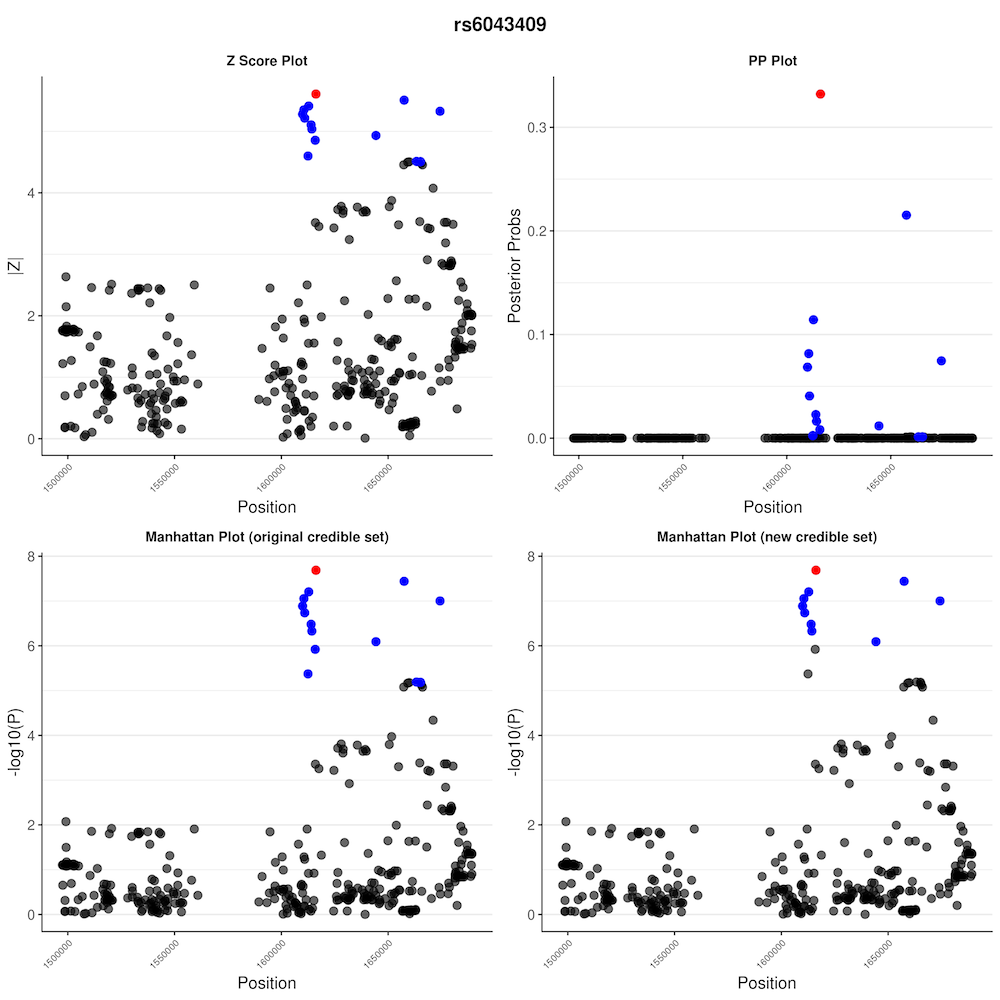

Supplement: S2 File — Zip file containing Z-score plots, PP plots and Manhattan plots for the 39 T1D association regions analysed. (ZIP) [file pcbi.1007829.s015.zip › S2_file/rs6043409.png]

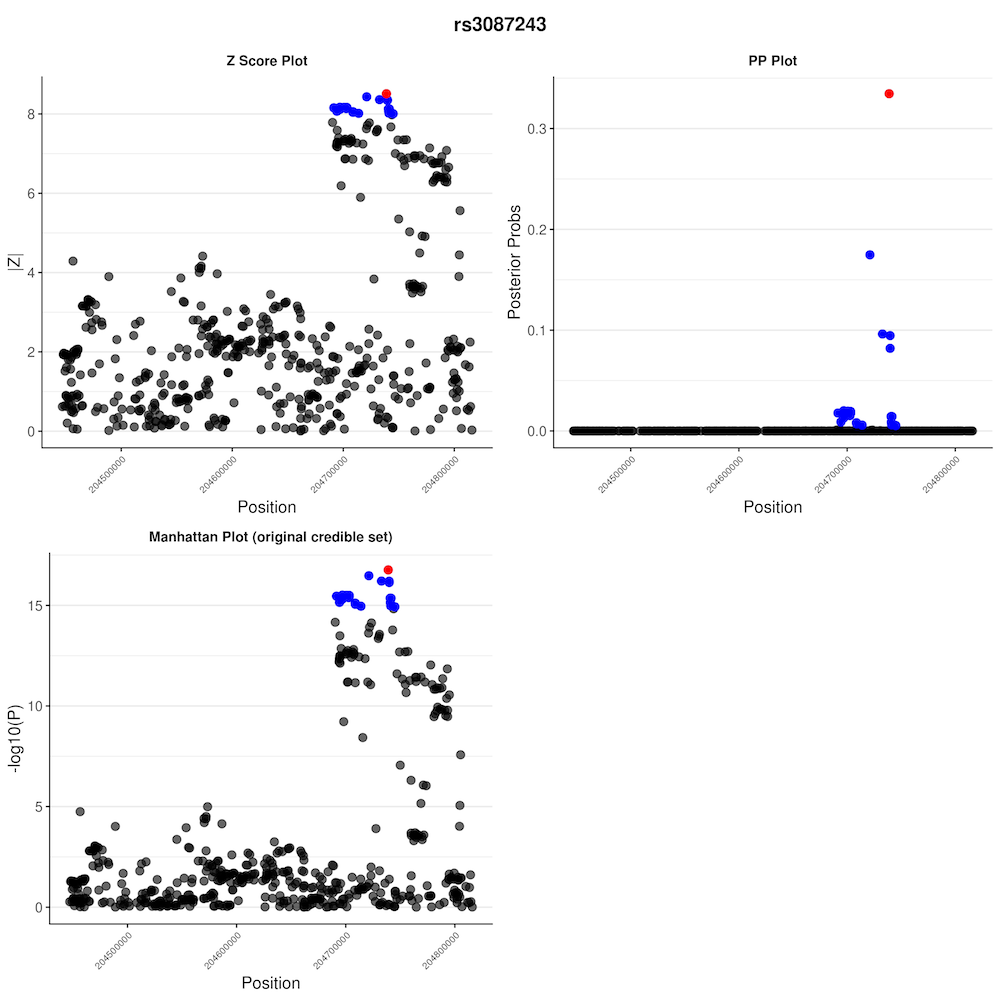

Supplement: S2 File — Zip file containing Z-score plots, PP plots and Manhattan plots for the 39 T1D association regions analysed. (ZIP) [file pcbi.1007829.s015.zip › S2_file/rs3087243.png]

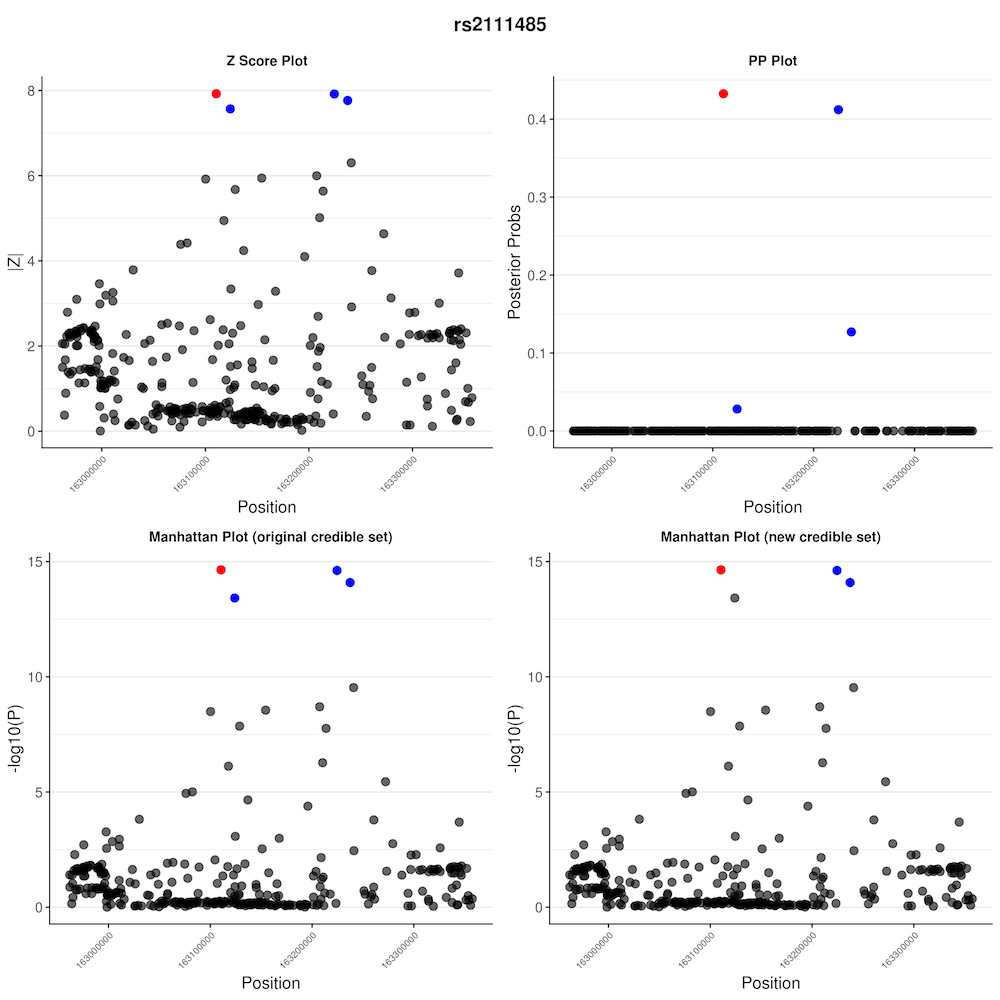

Supplement: S2 File — Zip file containing Z-score plots, PP plots and Manhattan plots for the 39 T1D association regions analysed. (ZIP) [file pcbi.1007829.s015.zip › S2_file/rs2111485.png]

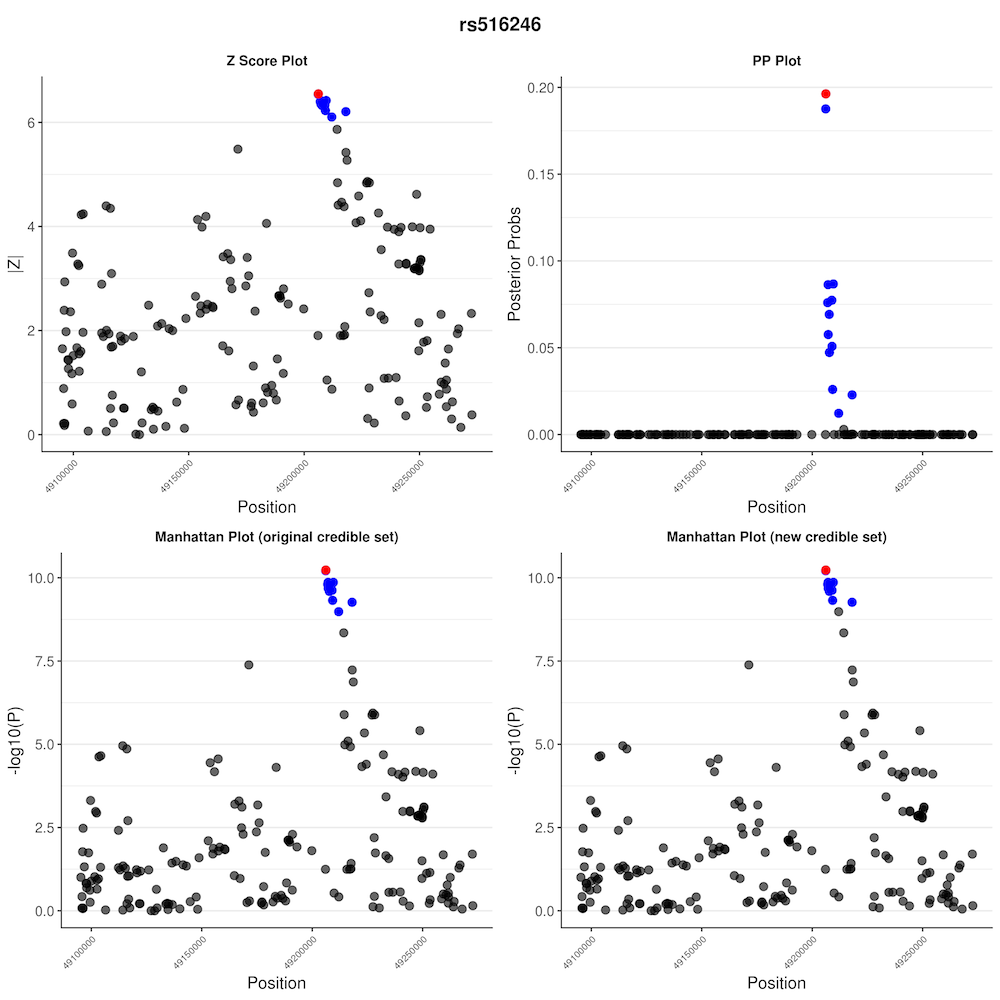

Supplement: S2 File — Zip file containing Z-score plots, PP plots and Manhattan plots for the 39 T1D association regions analysed. (ZIP) [file pcbi.1007829.s015.zip › S2_file/rs516246.png]

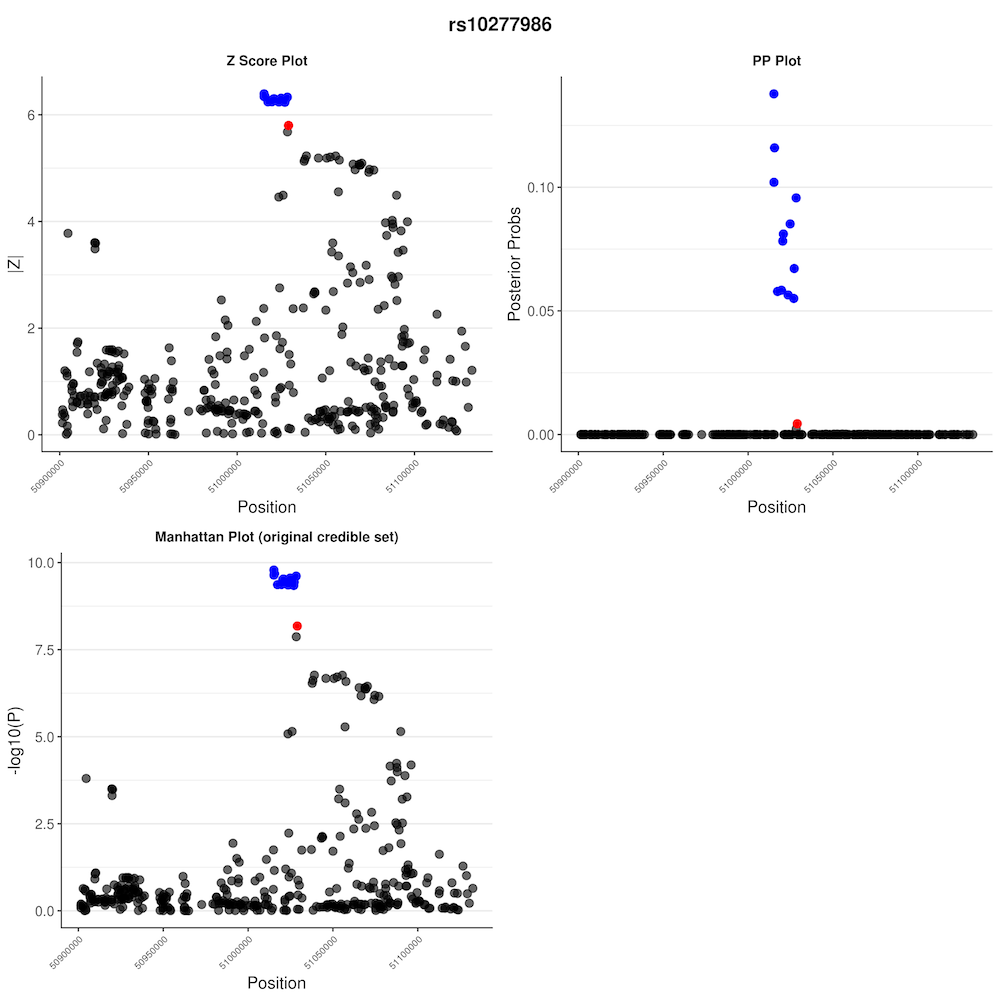

Supplement: S2 File — Zip file containing Z-score plots, PP plots and Manhattan plots for the 39 T1D association regions analysed. (ZIP) [file pcbi.1007829.s015.zip › S2_file/rs10277986.png]

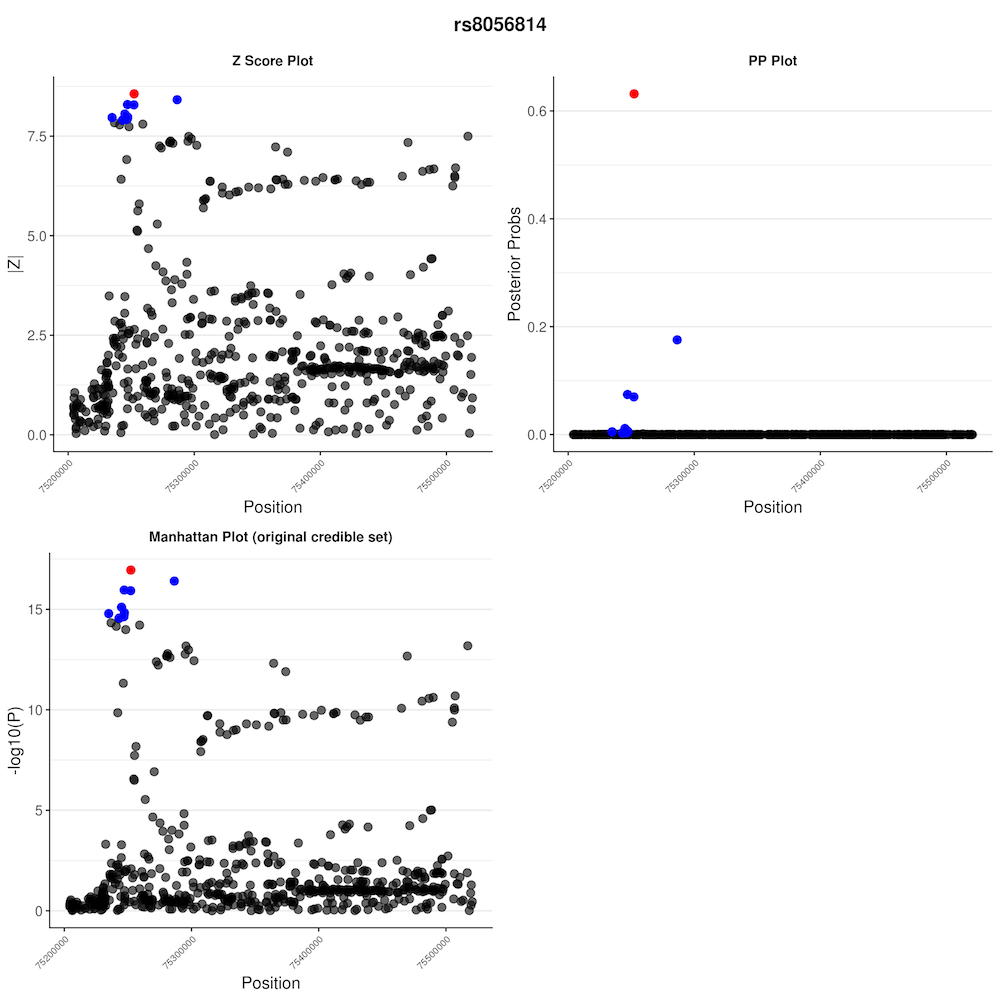

Supplement: S2 File — Zip file containing Z-score plots, PP plots and Manhattan plots for the 39 T1D association regions analysed. (ZIP) [file pcbi.1007829.s015.zip › S2_file/rs8056814.png]

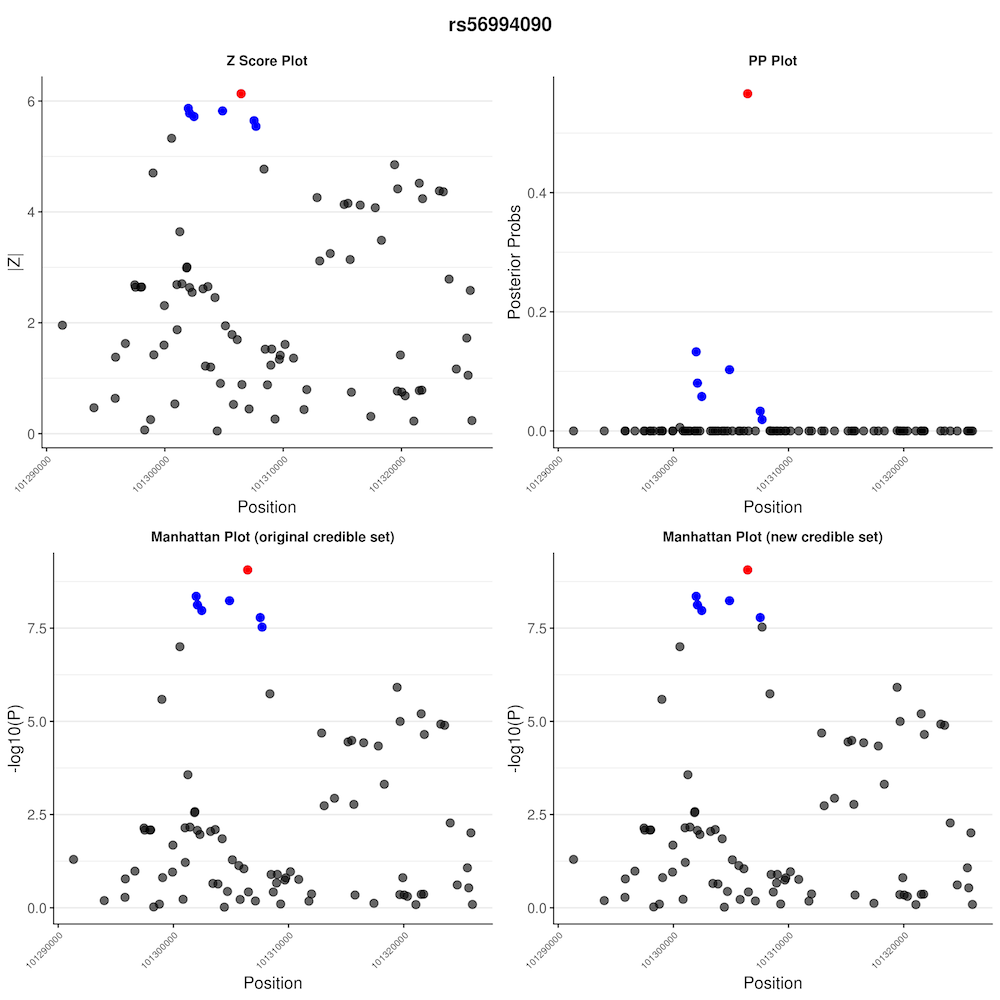

Supplement: S2 File — Zip file containing Z-score plots, PP plots and Manhattan plots for the 39 T1D association regions analysed. (ZIP) [file pcbi.1007829.s015.zip › S2_file/rs56994090.png]

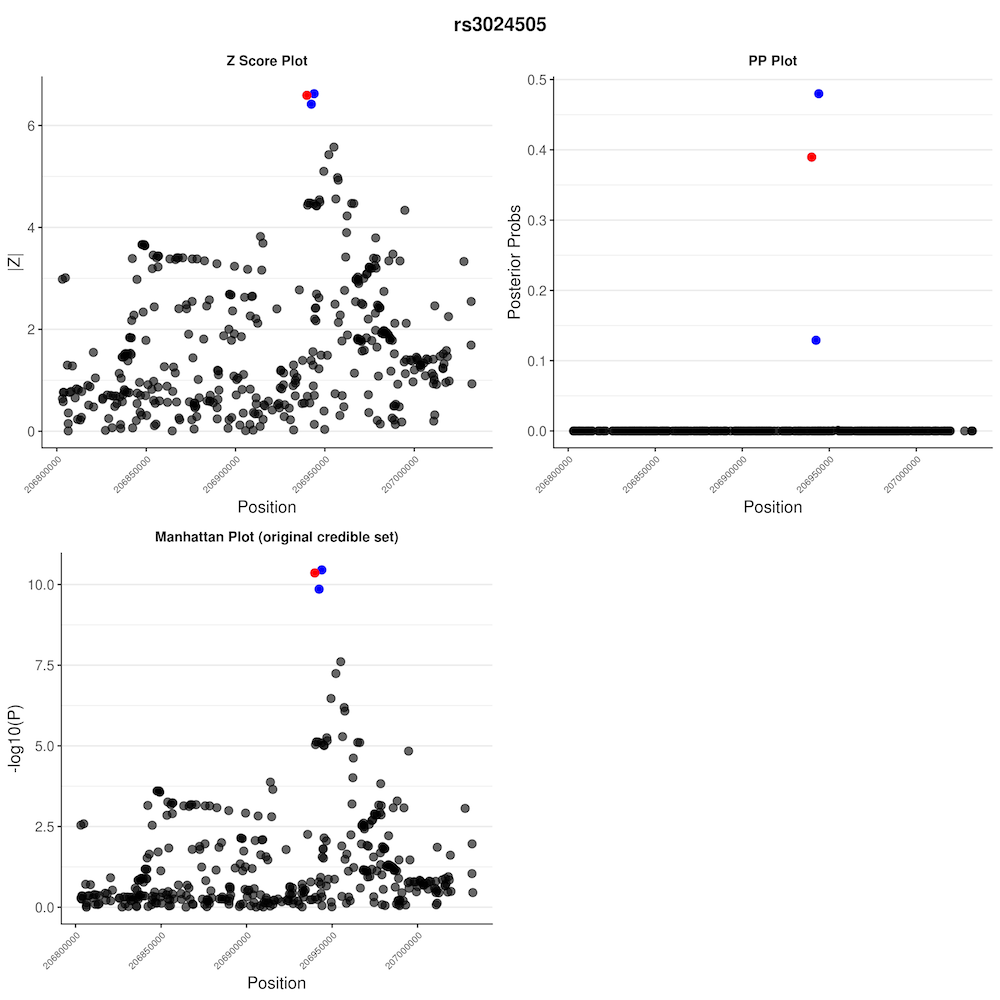

Supplement: S2 File — Zip file containing Z-score plots, PP plots and Manhattan plots for the 39 T1D association regions analysed. (ZIP) [file pcbi.1007829.s015.zip › S2_file/rs3024505.png]

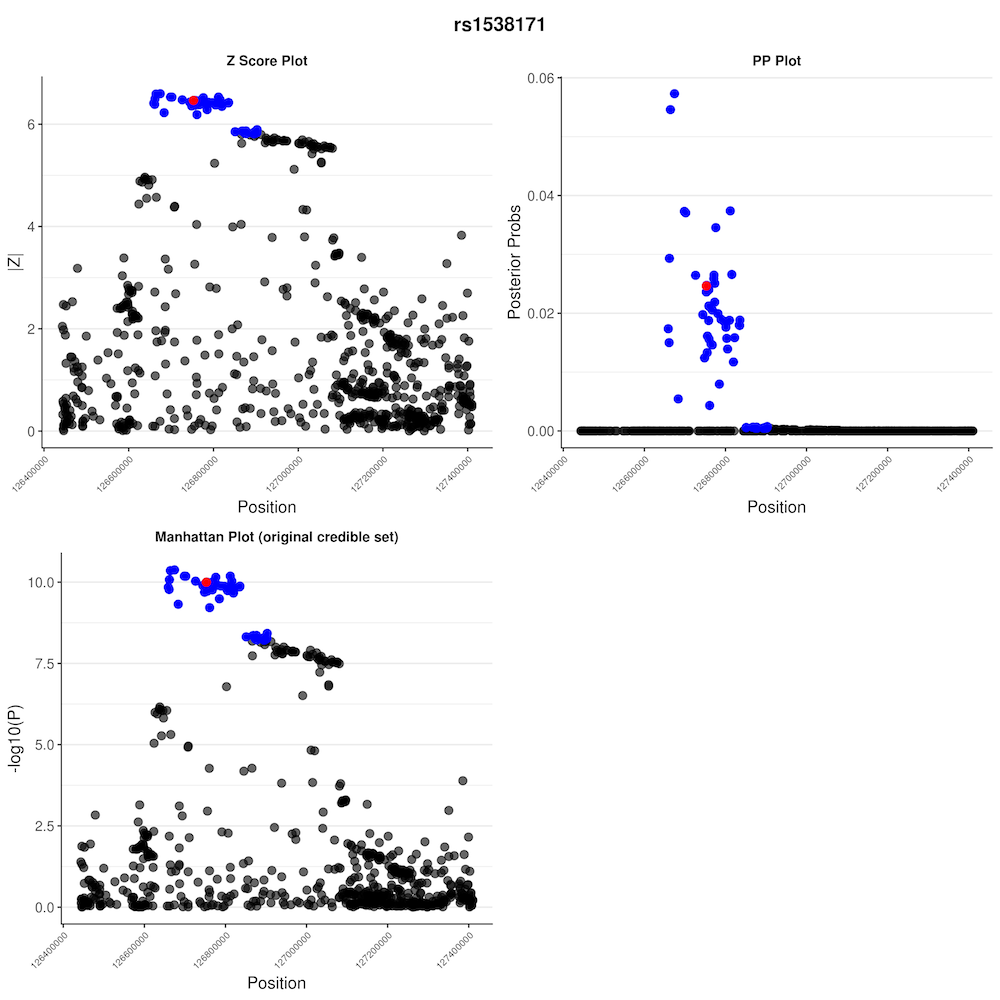

Supplement: S2 File — Zip file containing Z-score plots, PP plots and Manhattan plots for the 39 T1D association regions analysed. (ZIP) [file pcbi.1007829.s015.zip › S2_file/rs1538171.png]

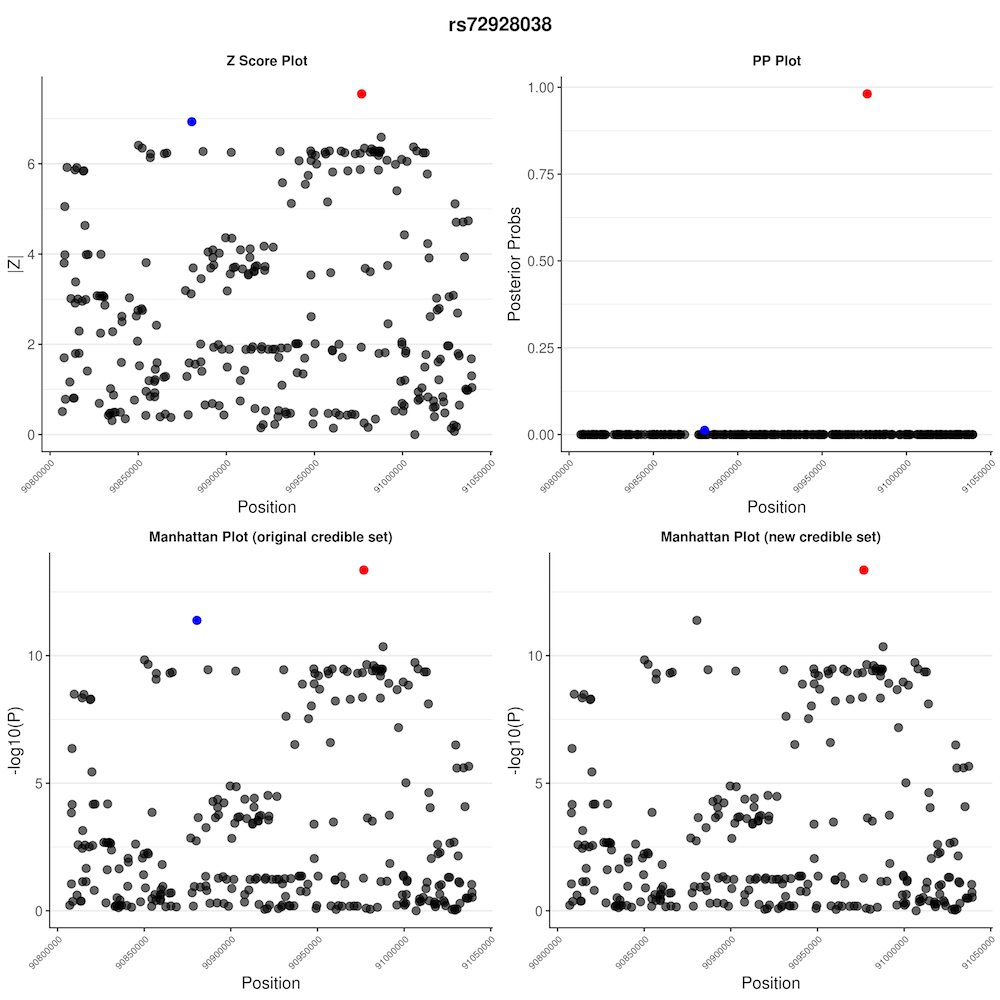

Supplement: S2 File — Zip file containing Z-score plots, PP plots and Manhattan plots for the 39 T1D association regions analysed. (ZIP) [file pcbi.1007829.s015.zip › S2_file/rs72928038.png]

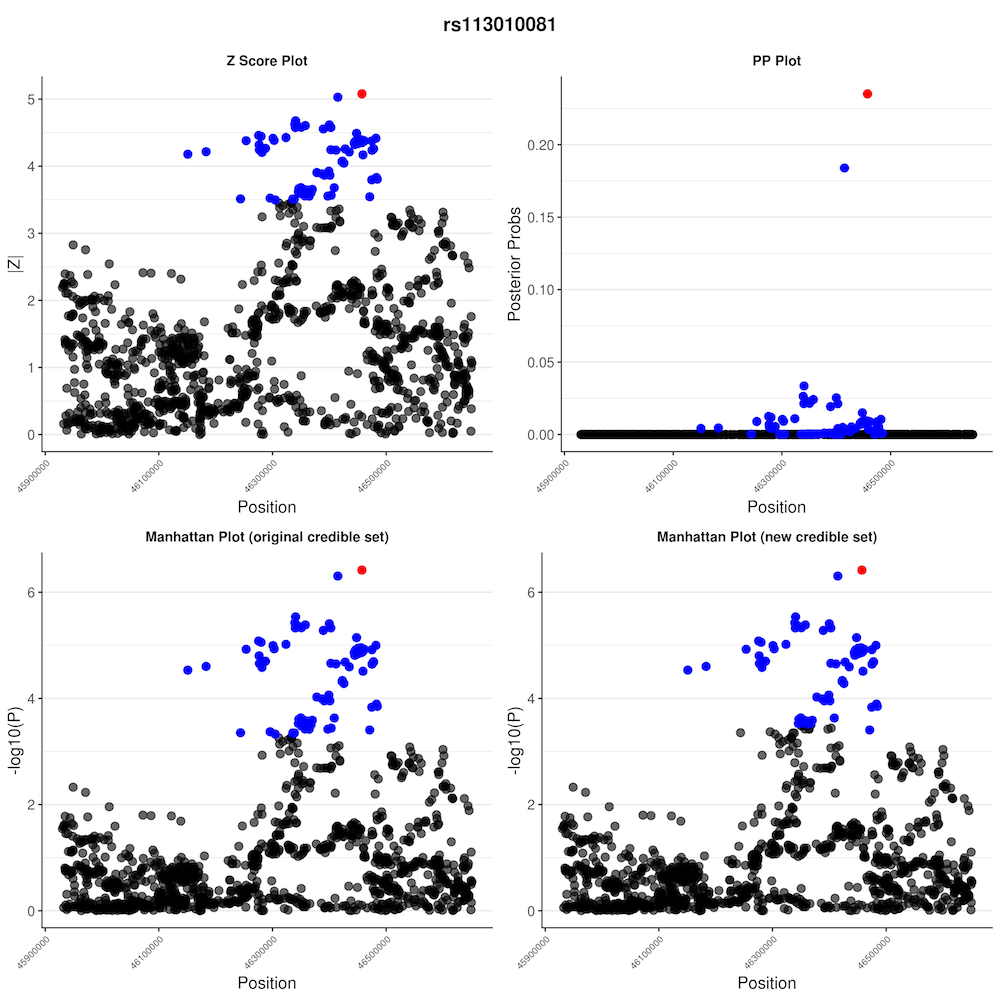

Supplement: S2 File — Zip file containing Z-score plots, PP plots and Manhattan plots for the 39 T1D association regions analysed. (ZIP) [file pcbi.1007829.s015.zip › S2_file/rs113010081.png]

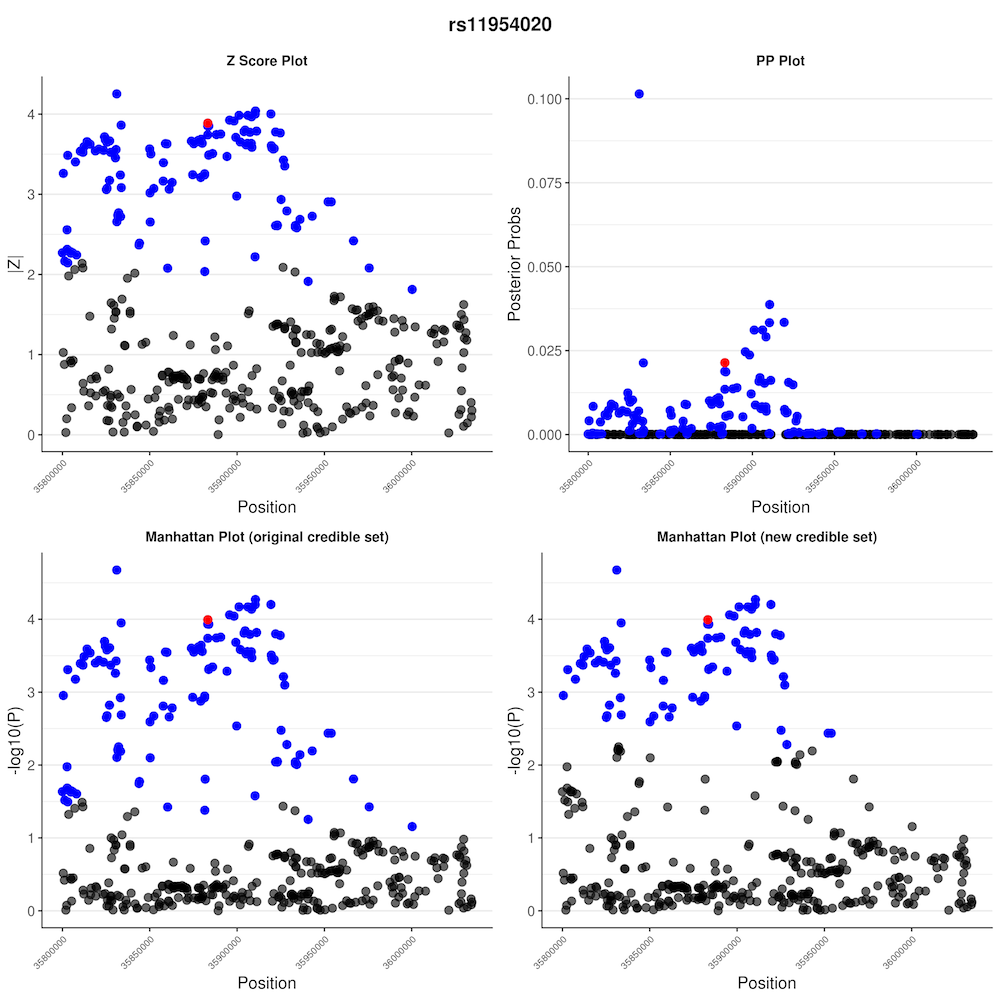

Supplement: S2 File — Zip file containing Z-score plots, PP plots and Manhattan plots for the 39 T1D association regions analysed. (ZIP) [file pcbi.1007829.s015.zip › S2_file/rs11954020.png]

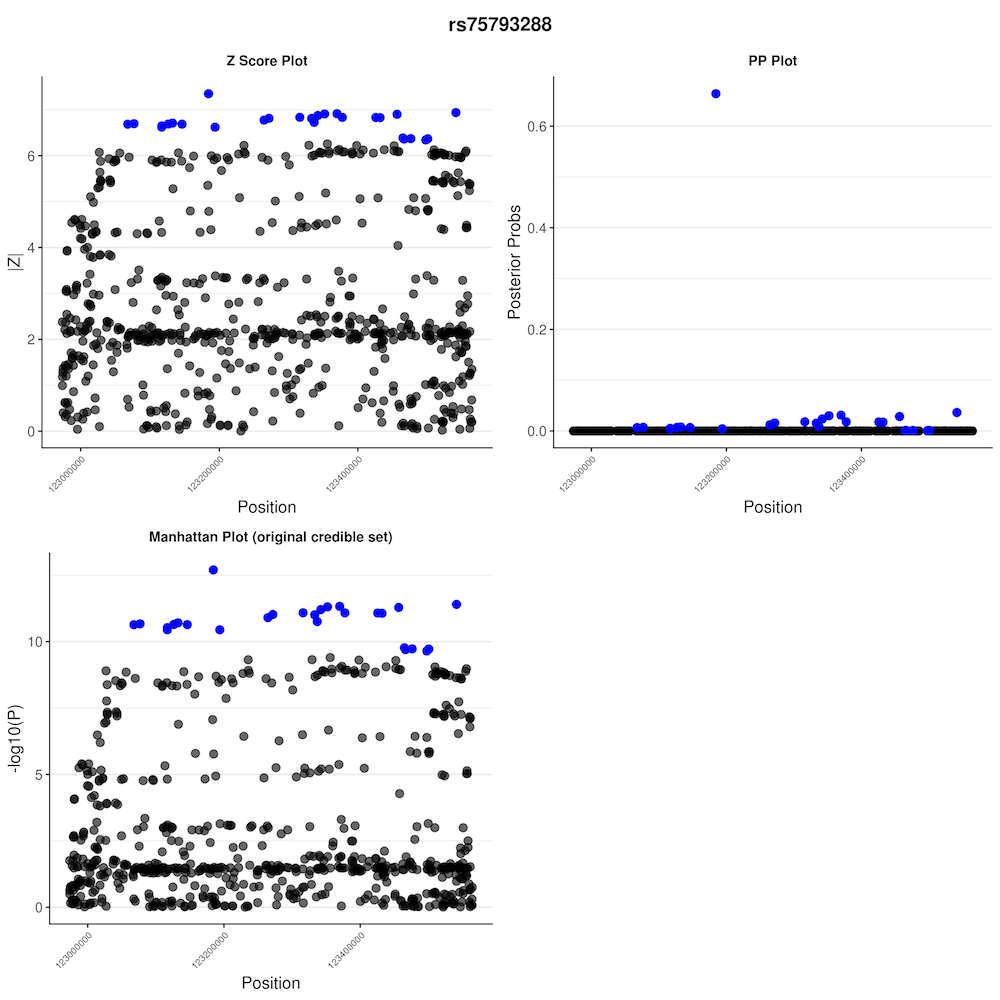

Supplement: S2 File — Zip file containing Z-score plots, PP plots and Manhattan plots for the 39 T1D association regions analysed. (ZIP) [file pcbi.1007829.s015.zip › S2_file/rs75793288.png]

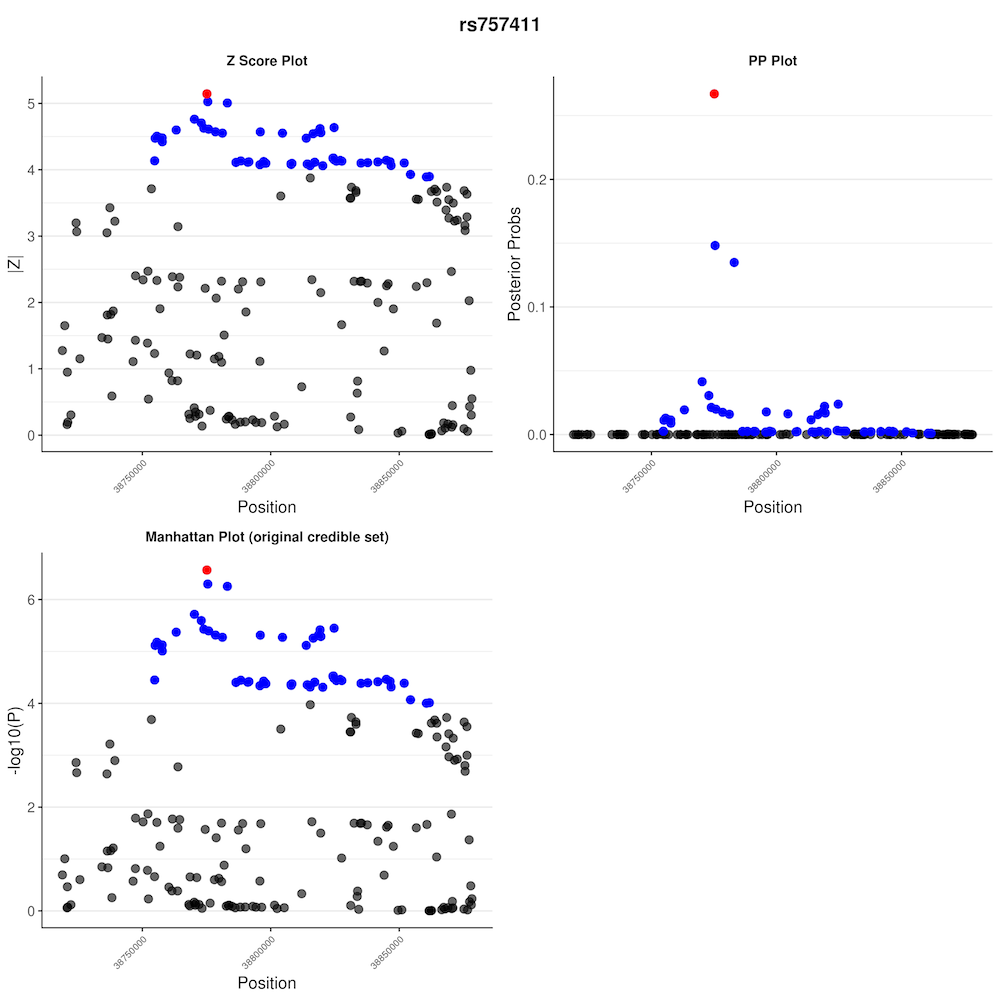

Supplement: S2 File — Zip file containing Z-score plots, PP plots and Manhattan plots for the 39 T1D association regions analysed. (ZIP) [file pcbi.1007829.s015.zip › S2_file/rs757411.png]

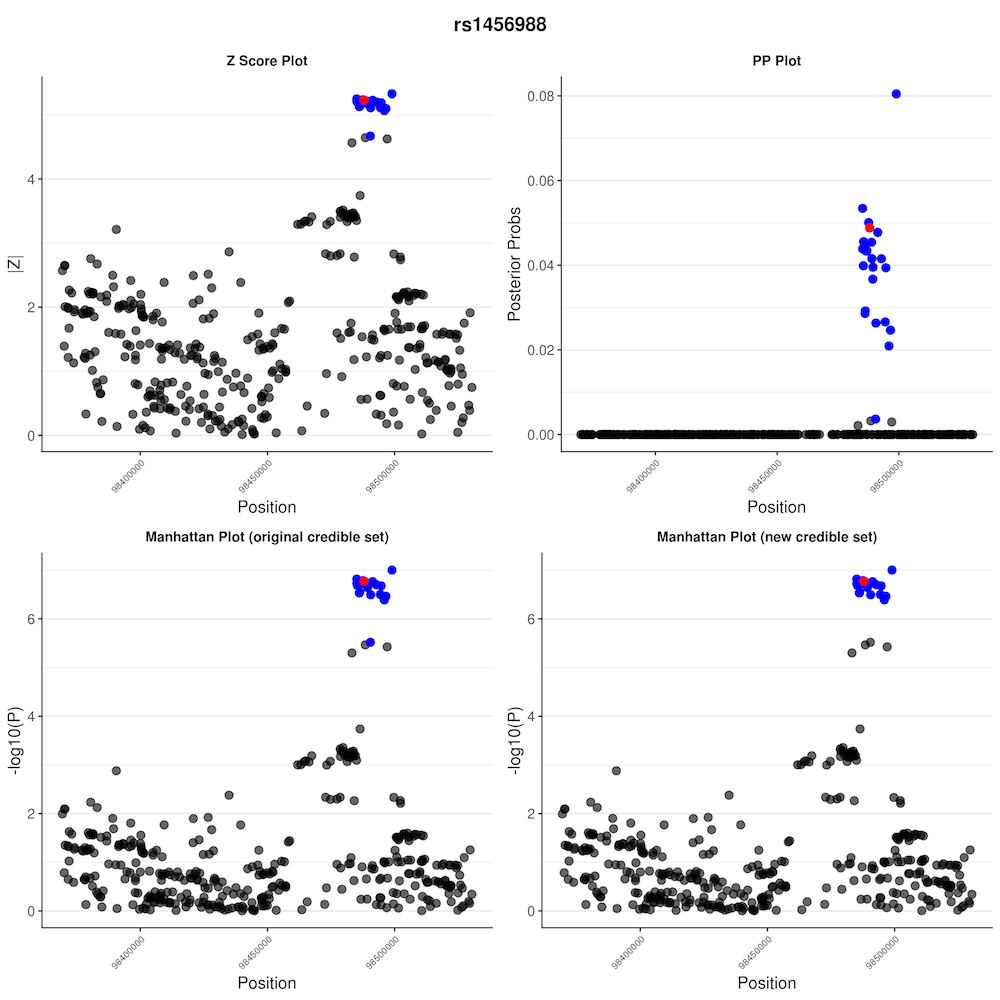

Supplement: S2 File — Zip file containing Z-score plots, PP plots and Manhattan plots for the 39 T1D association regions analysed. (ZIP) [file pcbi.1007829.s015.zip › S2_file/rs1456988.png]

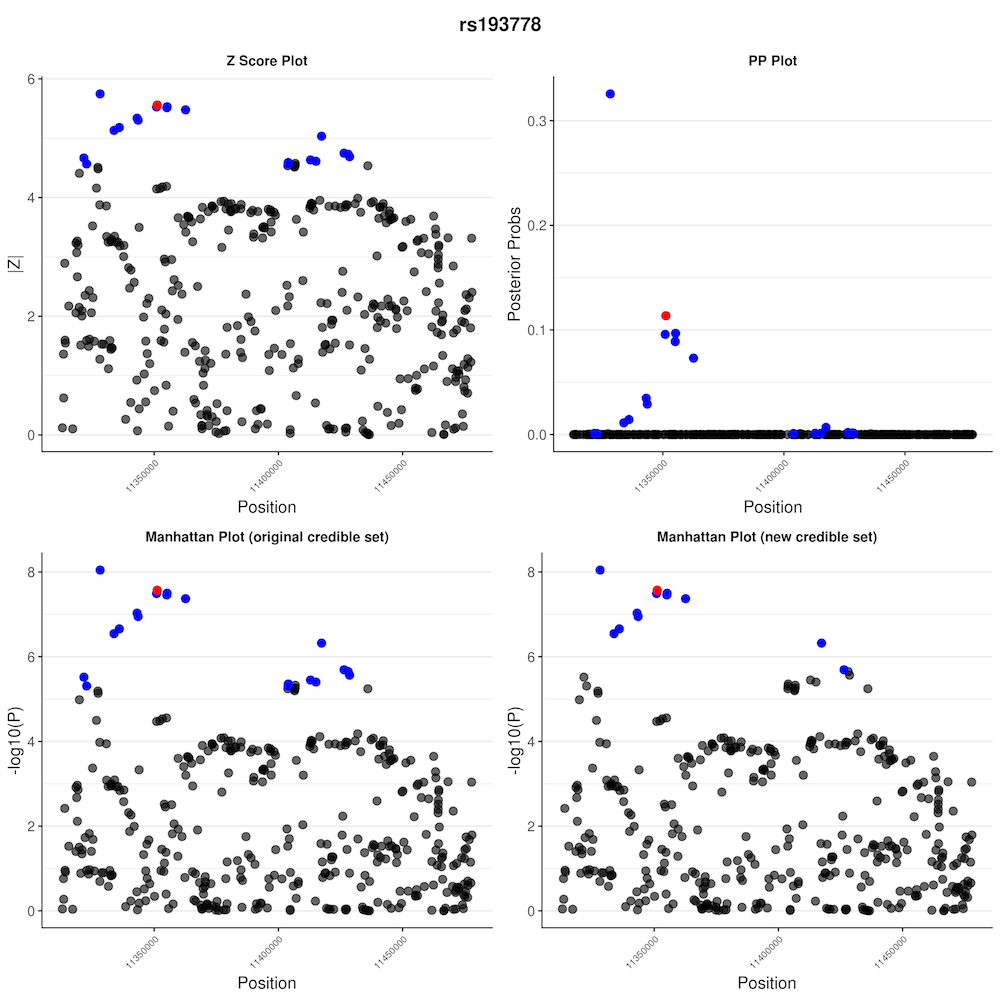

Supplement: S2 File — Zip file containing Z-score plots, PP plots and Manhattan plots for the 39 T1D association regions analysed. (ZIP) [file pcbi.1007829.s015.zip › S2_file/rs193778.png]

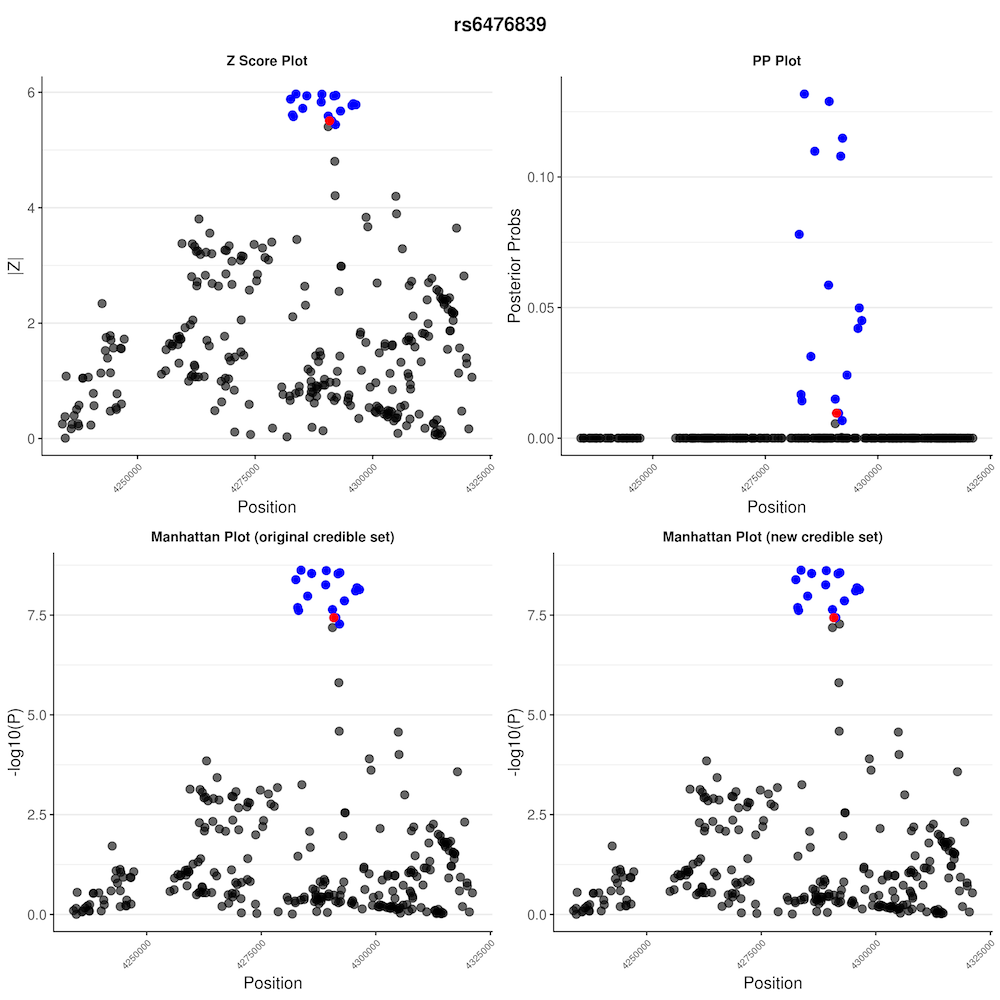

Supplement: S2 File — Zip file containing Z-score plots, PP plots and Manhattan plots for the 39 T1D association regions analysed. (ZIP) [file pcbi.1007829.s015.zip › S2_file/rs6476839.png]

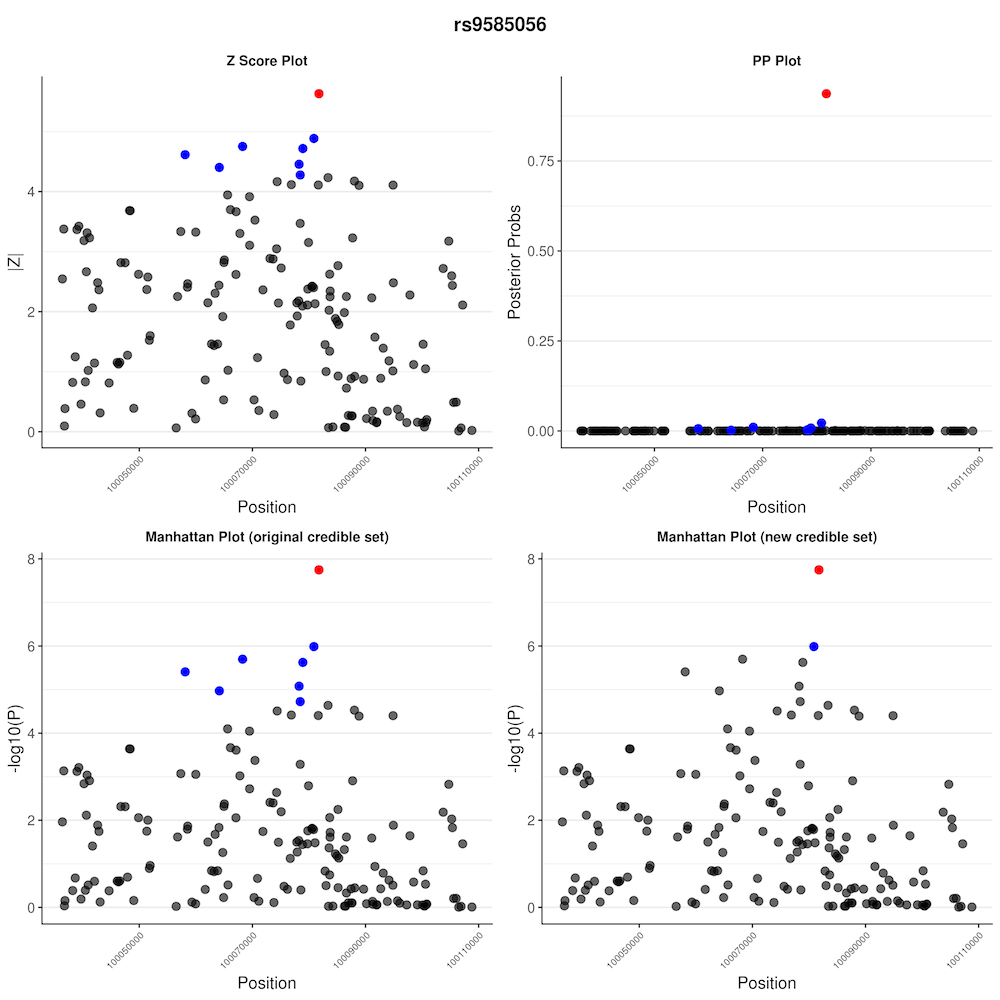

Supplement: S2 File — Zip file containing Z-score plots, PP plots and Manhattan plots for the 39 T1D association regions analysed. (ZIP) [file pcbi.1007829.s015.zip › S2_file/rs9585056.png]

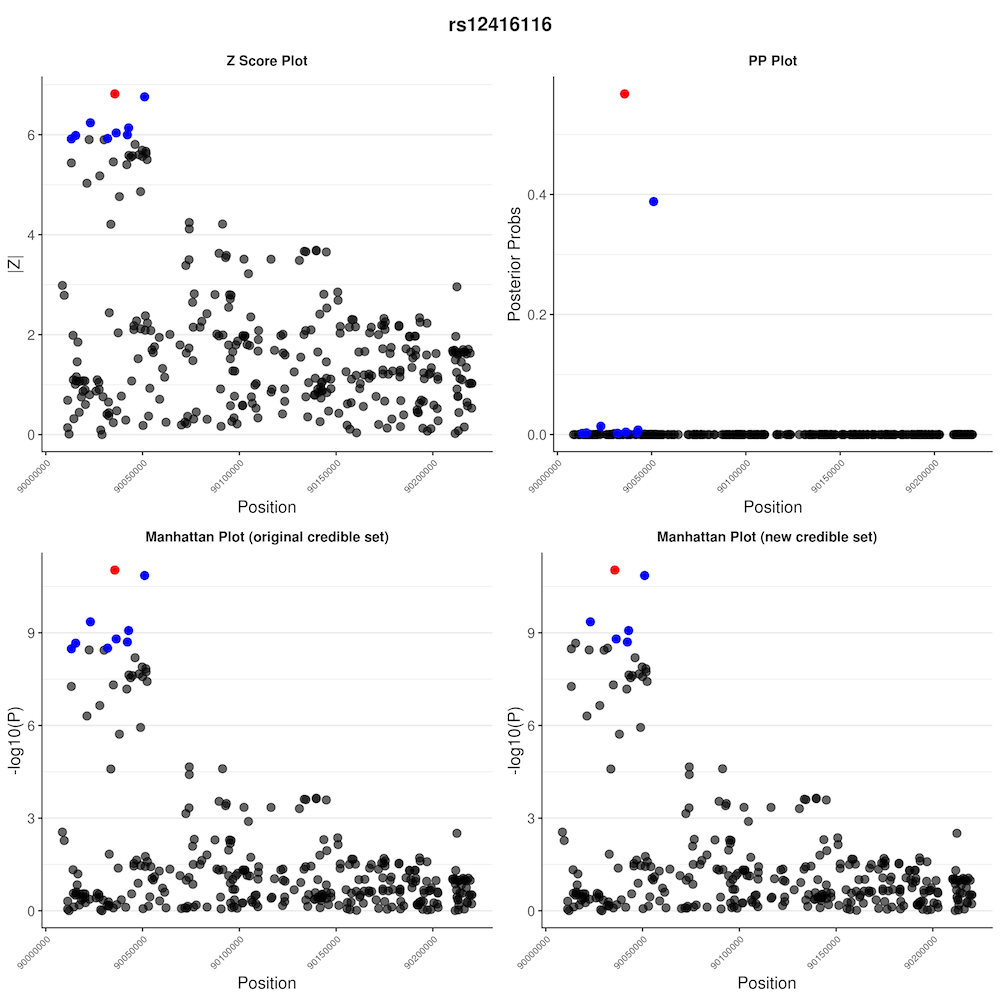

Supplement: S2 File — Zip file containing Z-score plots, PP plots and Manhattan plots for the 39 T1D association regions analysed. (ZIP) [file pcbi.1007829.s015.zip › S2_file/rs12416116.png]

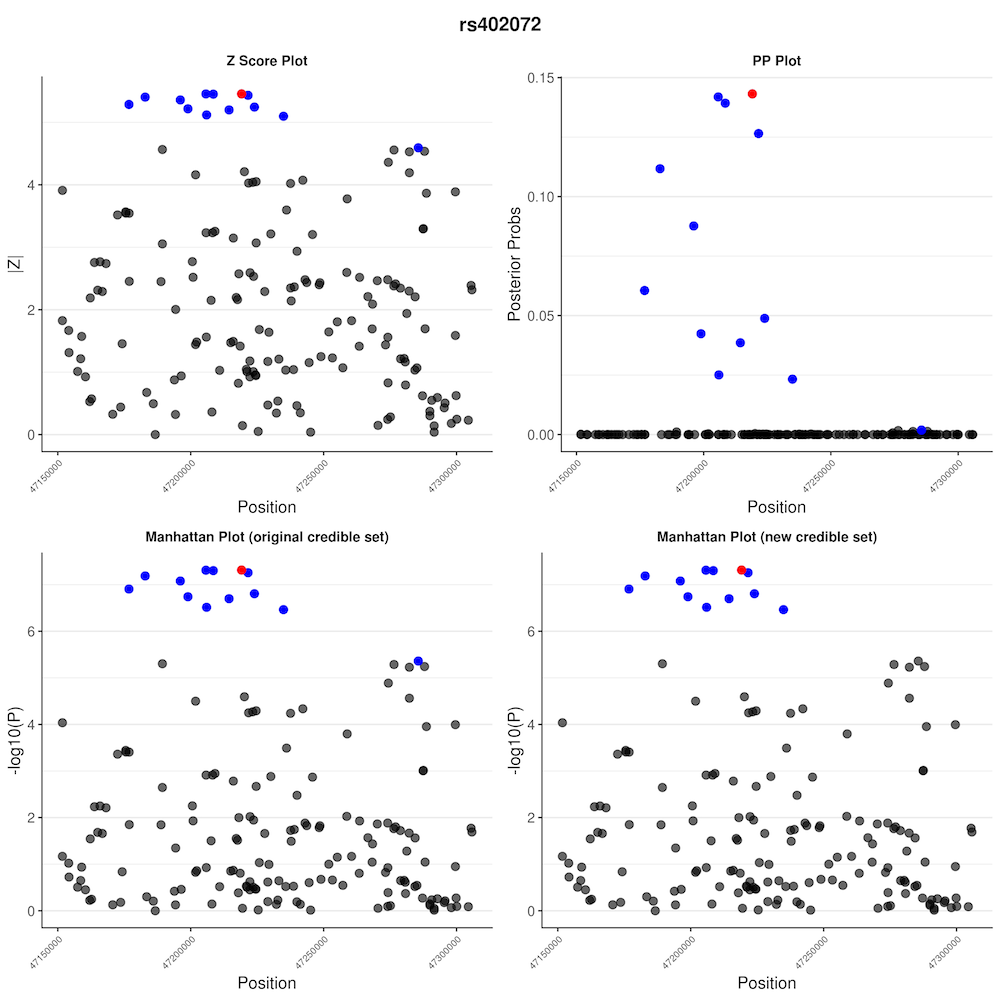

Supplement: S2 File — Zip file containing Z-score plots, PP plots and Manhattan plots for the 39 T1D association regions analysed. (ZIP) [file pcbi.1007829.s015.zip › S2_file/rs402072.png]

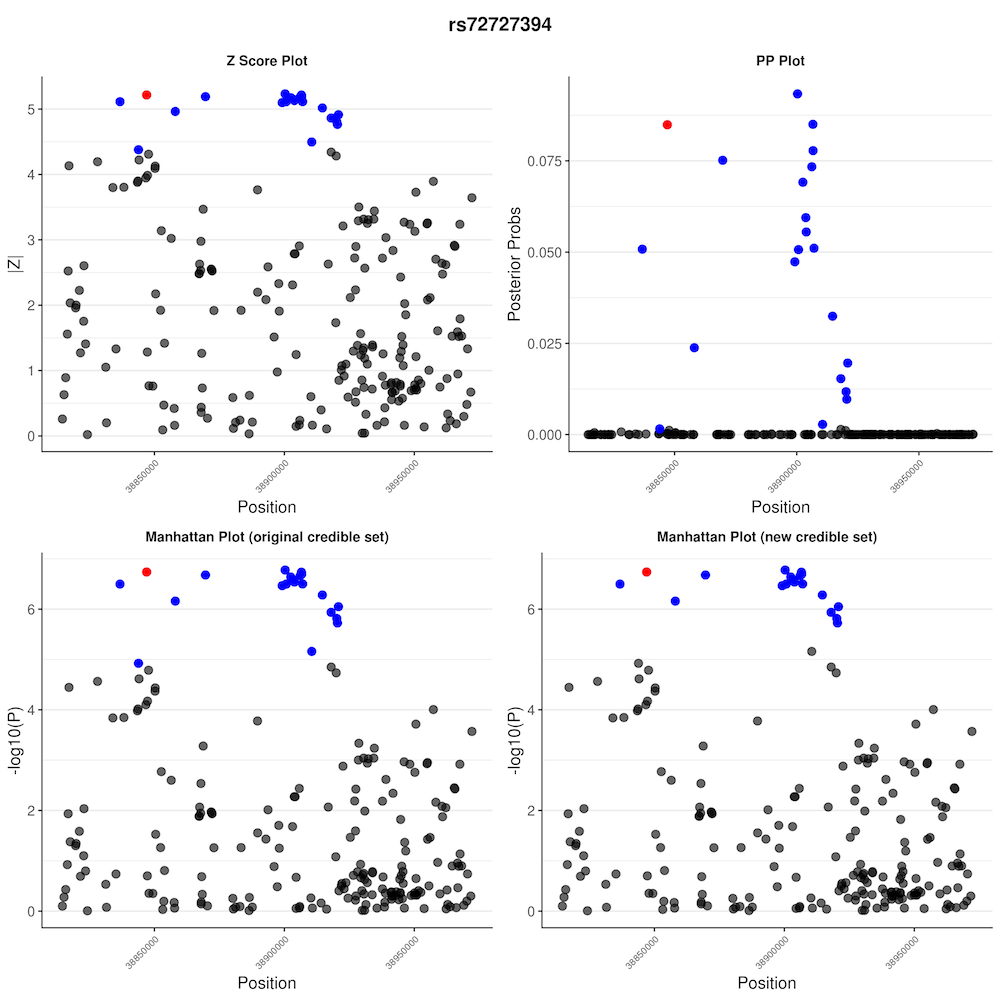

Supplement: S2 File — Zip file containing Z-score plots, PP plots and Manhattan plots for the 39 T1D association regions analysed. (ZIP) [file pcbi.1007829.s015.zip › S2_file/rs72727394.png]

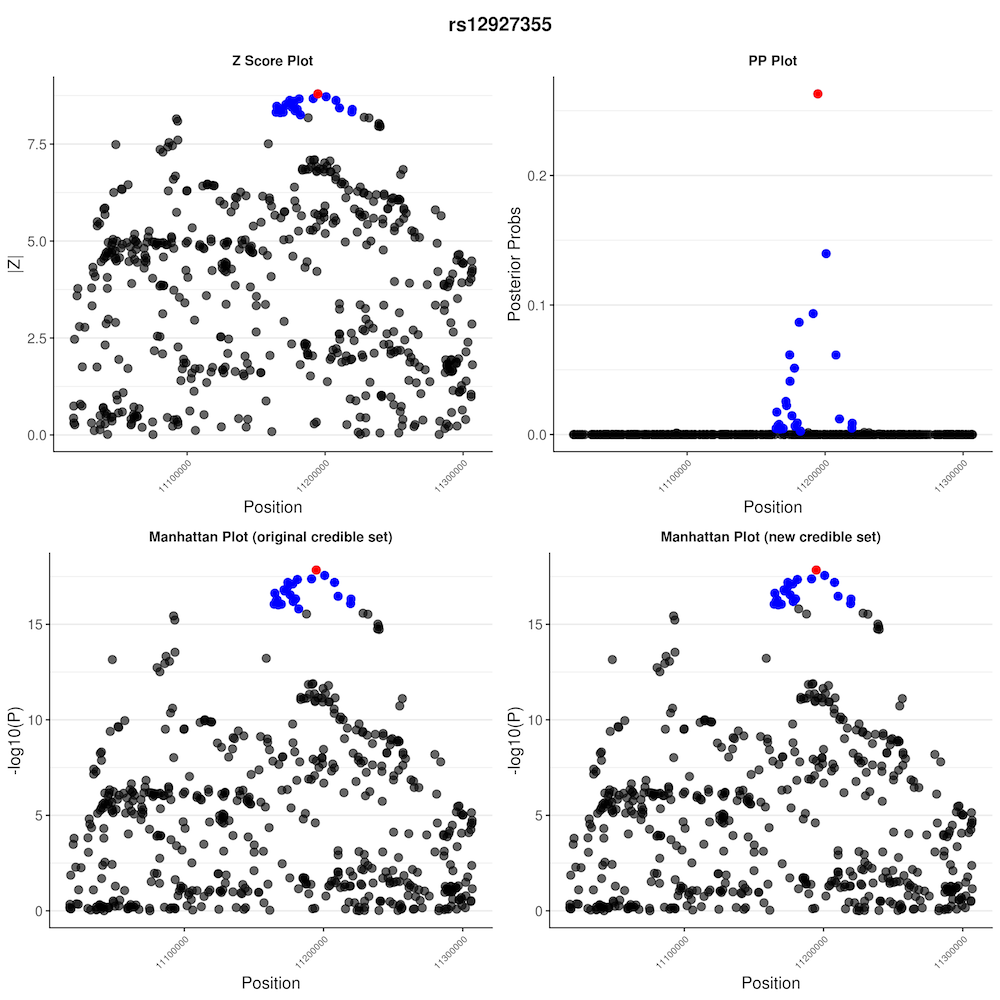

Supplement: S2 File — Zip file containing Z-score plots, PP plots and Manhattan plots for the 39 T1D association regions analysed. (ZIP) [file pcbi.1007829.s015.zip › S2_file/rs12927355.png]

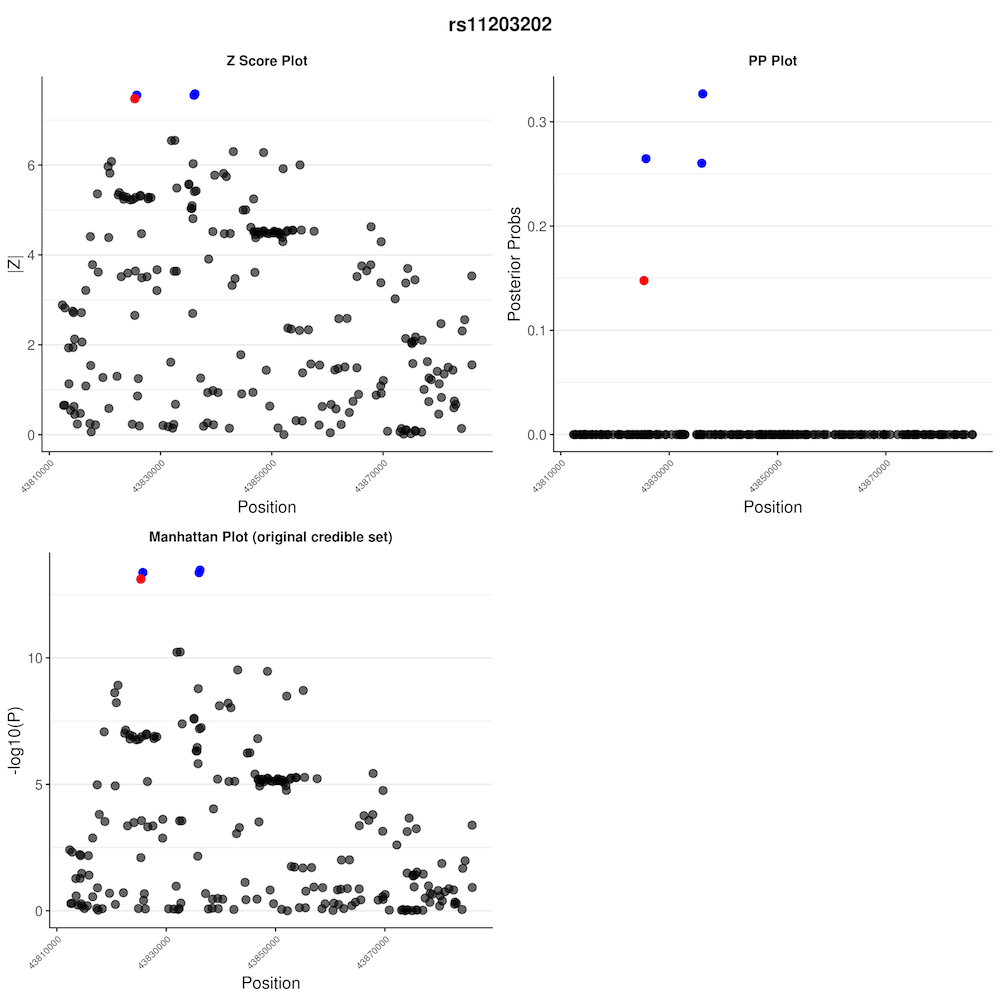

Supplement: S2 File — Zip file containing Z-score plots, PP plots and Manhattan plots for the 39 T1D association regions analysed. (ZIP) [file pcbi.1007829.s015.zip › S2_file/rs11203202.png]

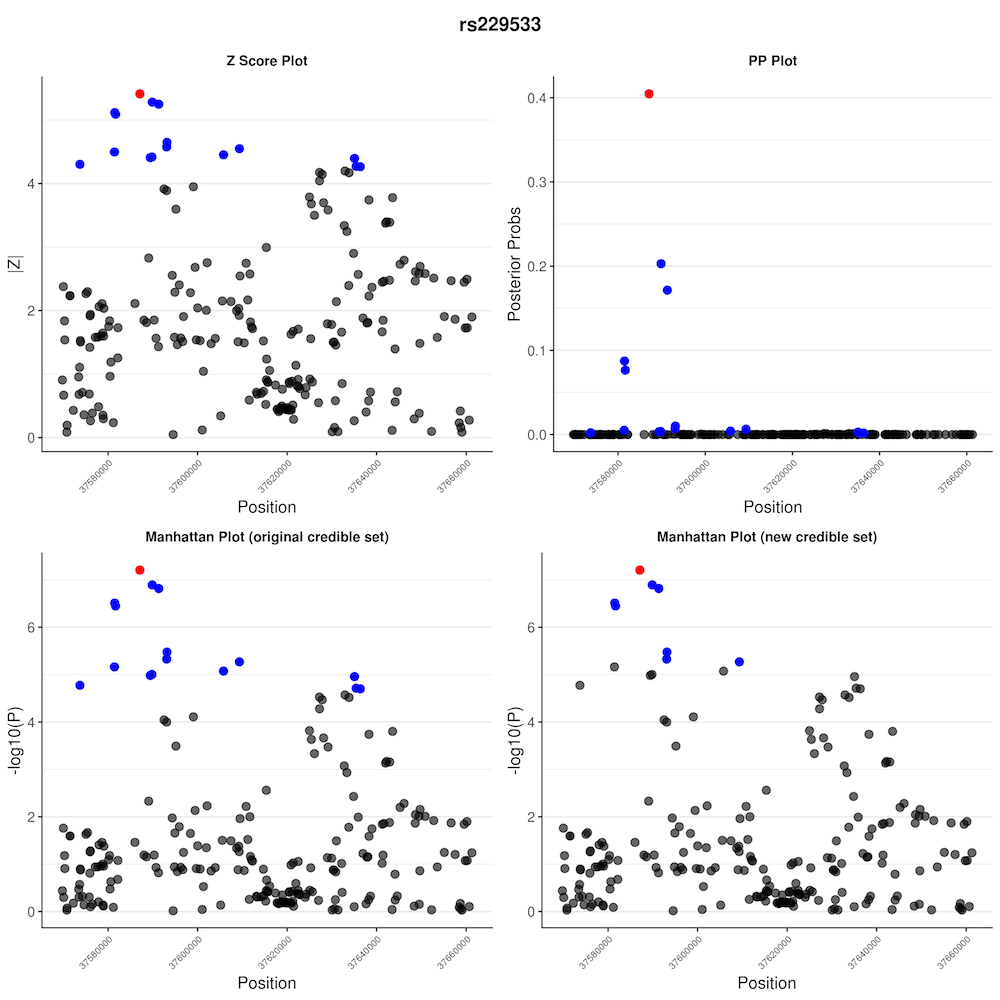

Supplement: S2 File — Zip file containing Z-score plots, PP plots and Manhattan plots for the 39 T1D association regions analysed. (ZIP) [file pcbi.1007829.s015.zip › S2_file/rs229533.png]

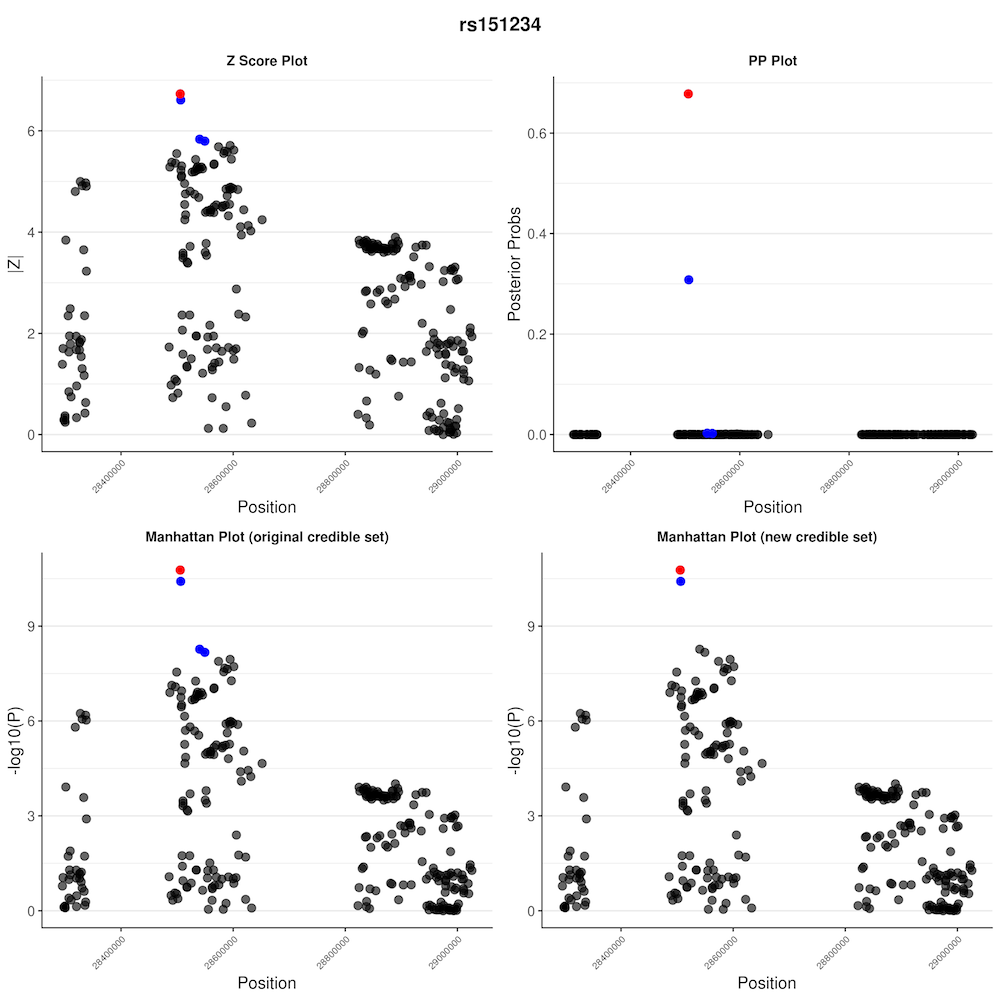

Supplement: S2 File — Zip file containing Z-score plots, PP plots and Manhattan plots for the 39 T1D association regions analysed. (ZIP) [file pcbi.1007829.s015.zip › S2_file/rs151234.png]

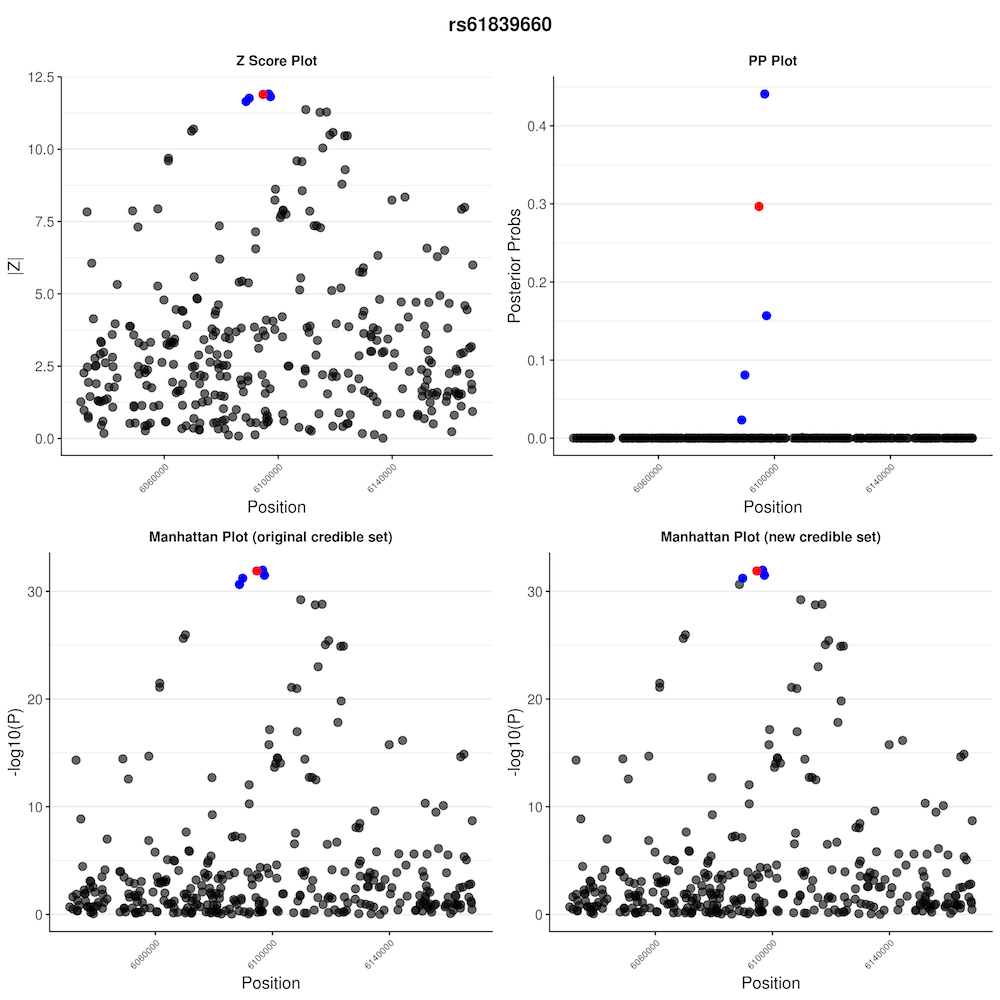

Supplement: S2 File — Zip file containing Z-score plots, PP plots and Manhattan plots for the 39 T1D association regions analysed. (ZIP) [file pcbi.1007829.s015.zip › S2_file/rs61839660.png]

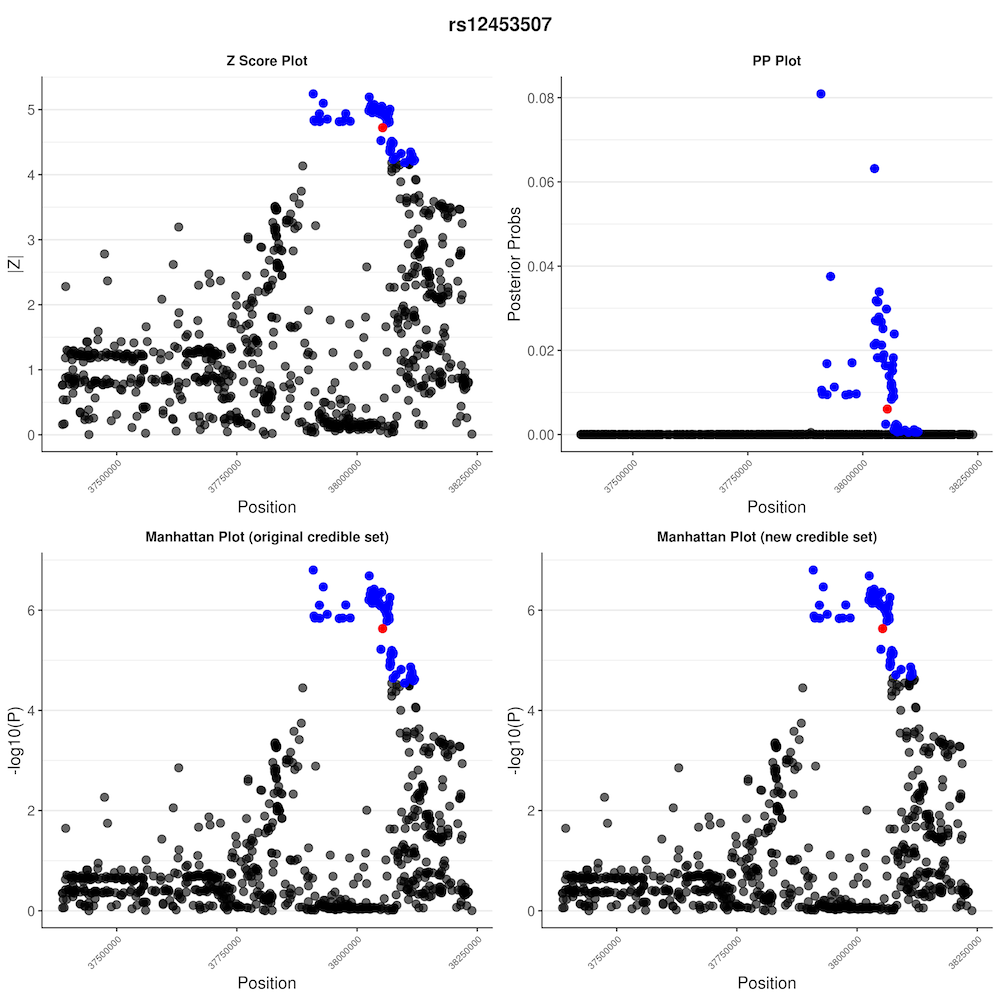

Supplement: S2 File — Zip file containing Z-score plots, PP plots and Manhattan plots for the 39 T1D association regions analysed. (ZIP) [file pcbi.1007829.s015.zip › S2_file/rs12453507.png]

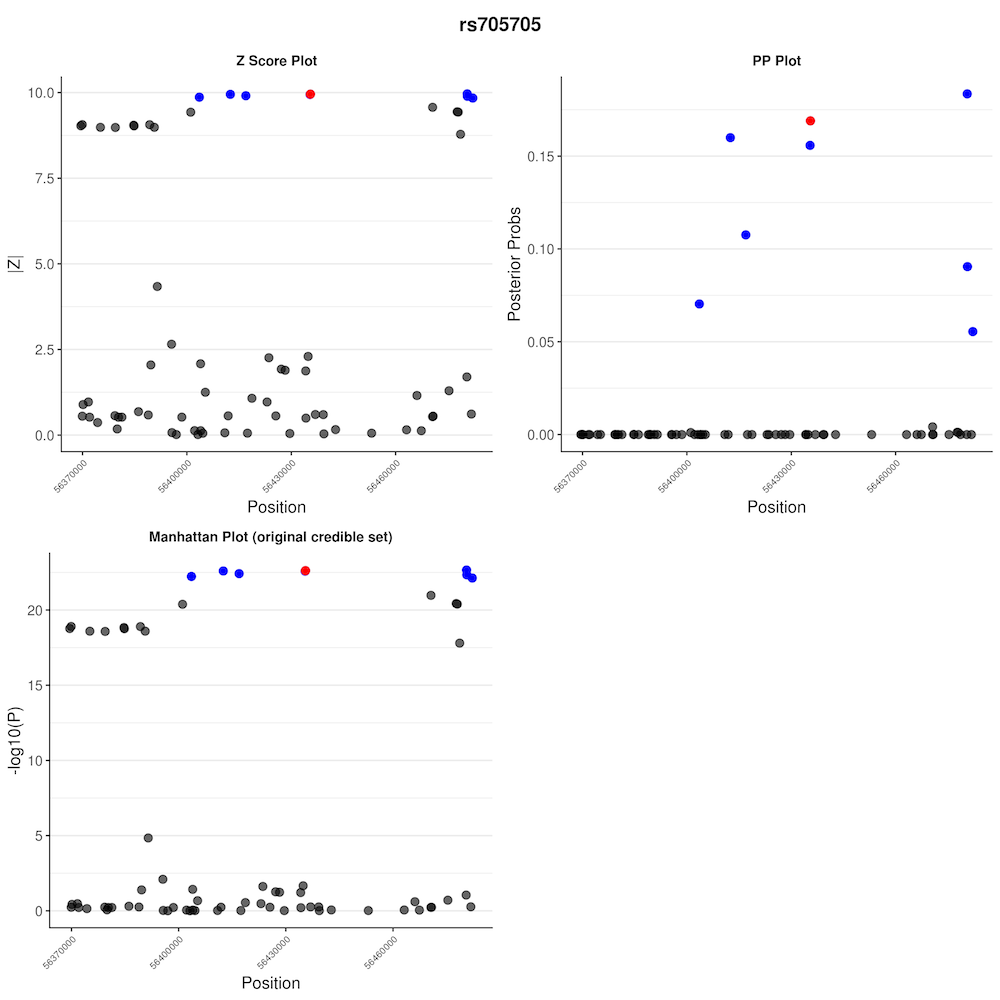

Supplement: S2 File — Zip file containing Z-score plots, PP plots and Manhattan plots for the 39 T1D association regions analysed. (ZIP) [file pcbi.1007829.s015.zip › S2_file/rs705705.png]

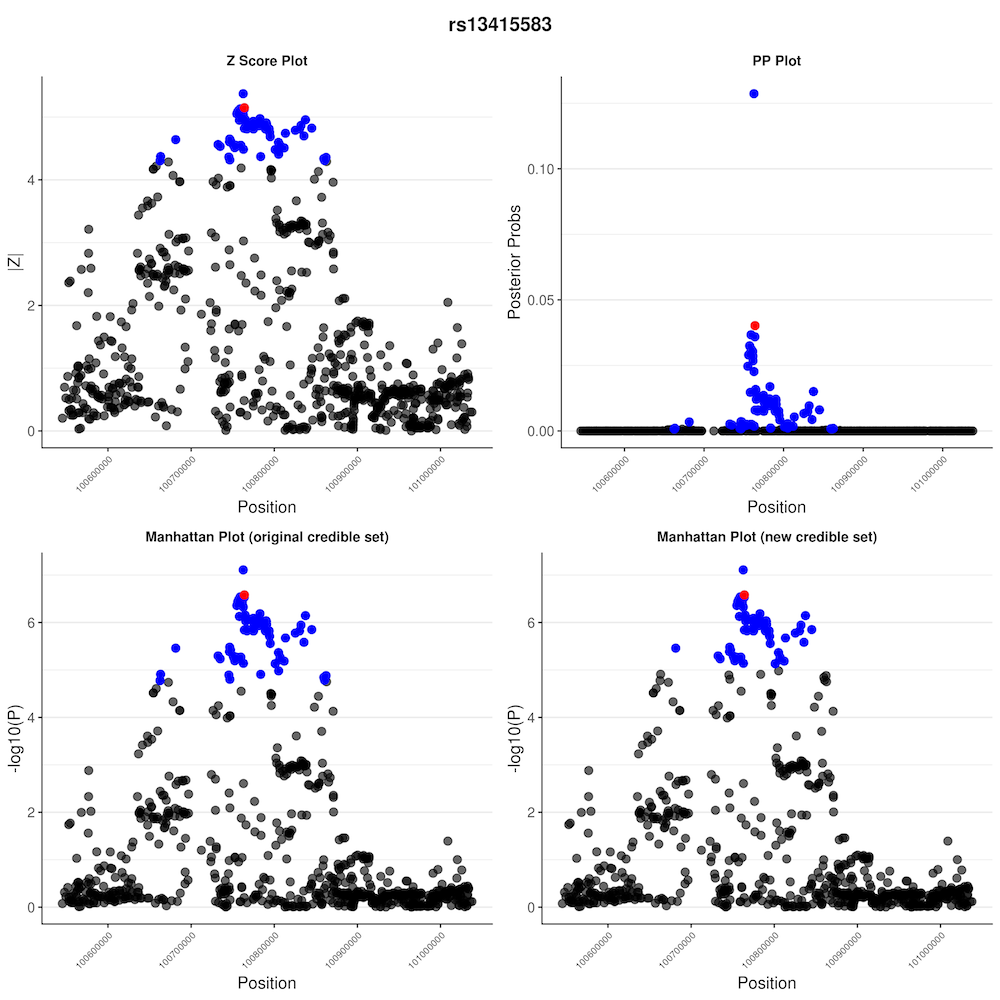

Supplement: S2 File — Zip file containing Z-score plots, PP plots and Manhattan plots for the 39 T1D association regions analysed. (ZIP) [file pcbi.1007829.s015.zip › S2_file/rs13415583.png]

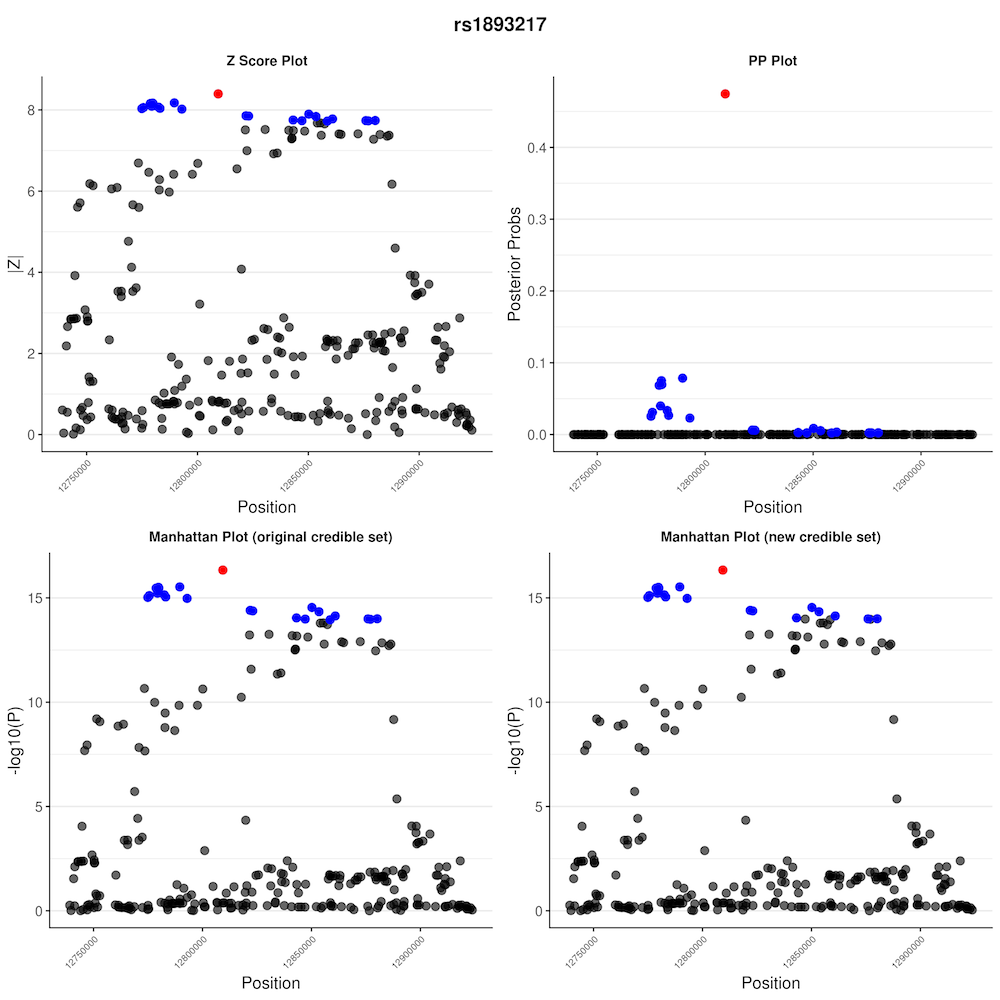

Supplement: S2 File — Zip file containing Z-score plots, PP plots and Manhattan plots for the 39 T1D association regions analysed. (ZIP) [file pcbi.1007829.s015.zip › S2_file/rs1893217.png]

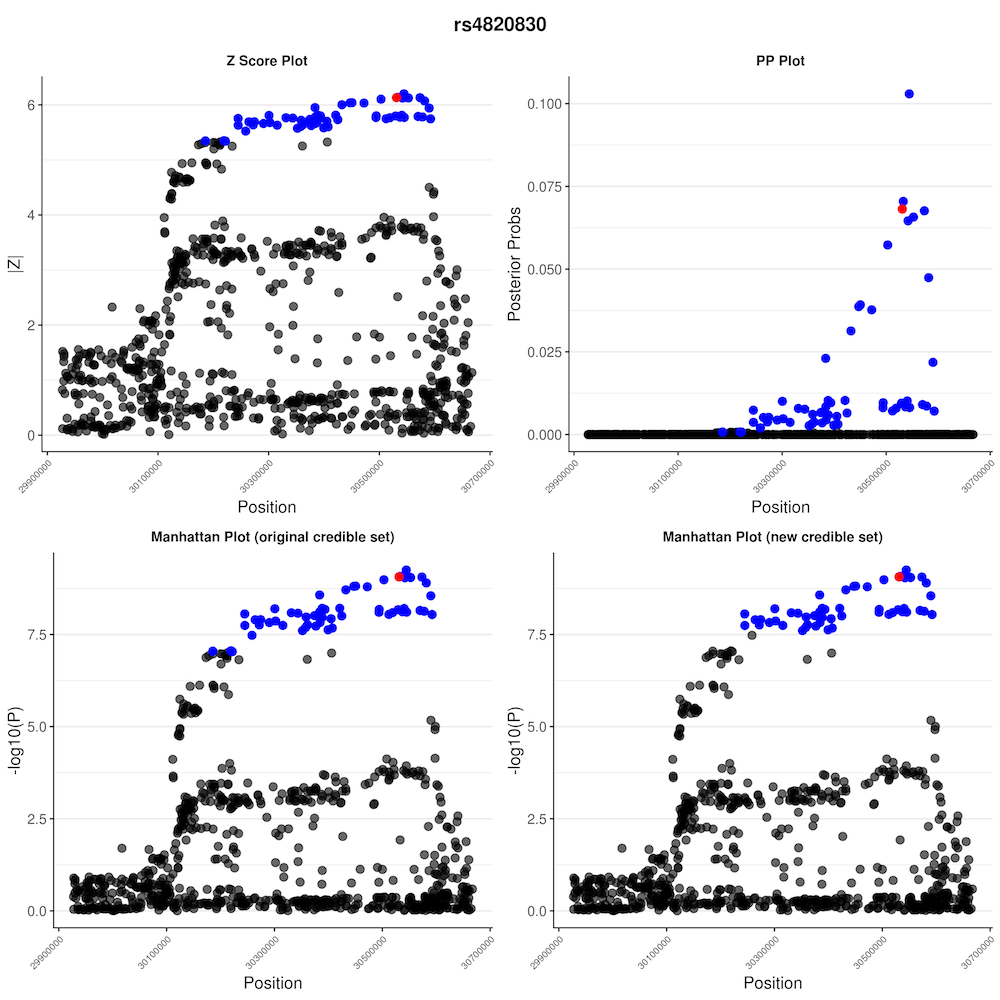

Supplement: S2 File — Zip file containing Z-score plots, PP plots and Manhattan plots for the 39 T1D association regions analysed. (ZIP) [file pcbi.1007829.s015.zip › S2_file/rs4820830.png]

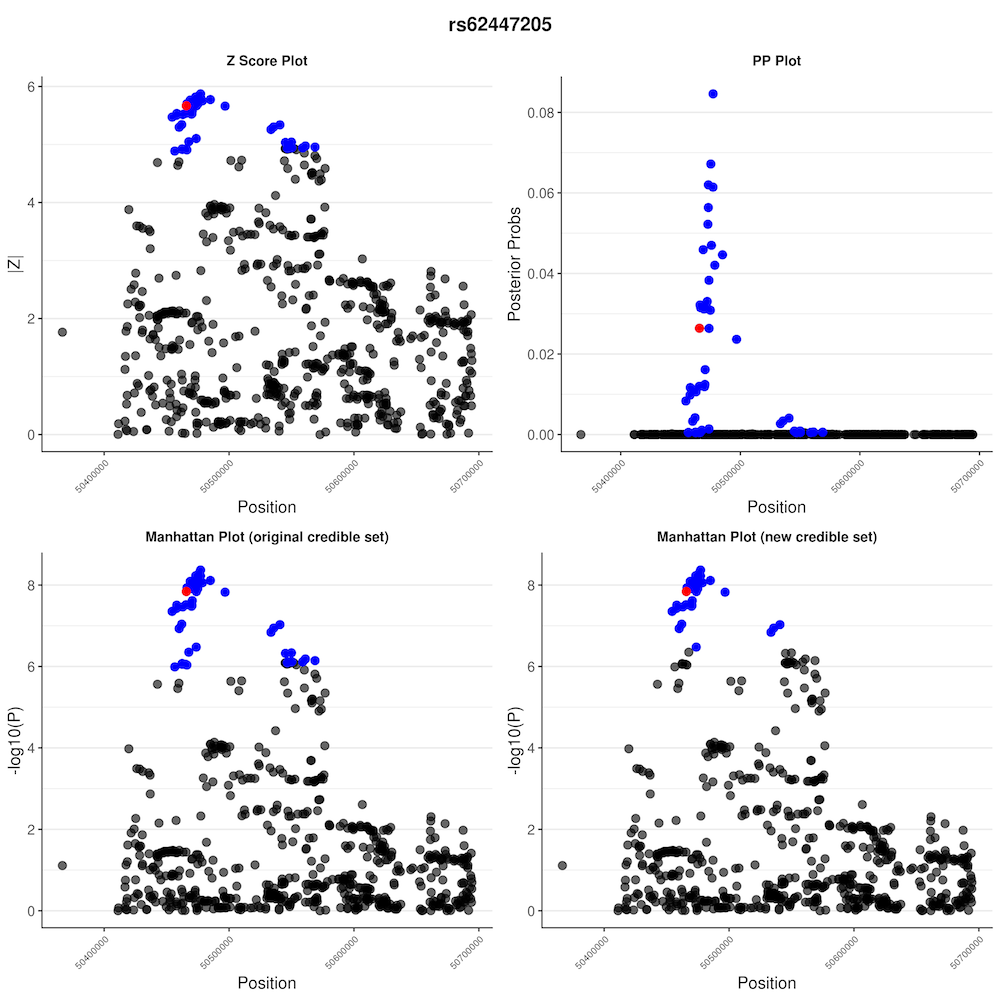

Supplement: S2 File — Zip file containing Z-score plots, PP plots and Manhattan plots for the 39 T1D association regions analysed. (ZIP) [file pcbi.1007829.s015.zip › S2_file/rs62447205.png]

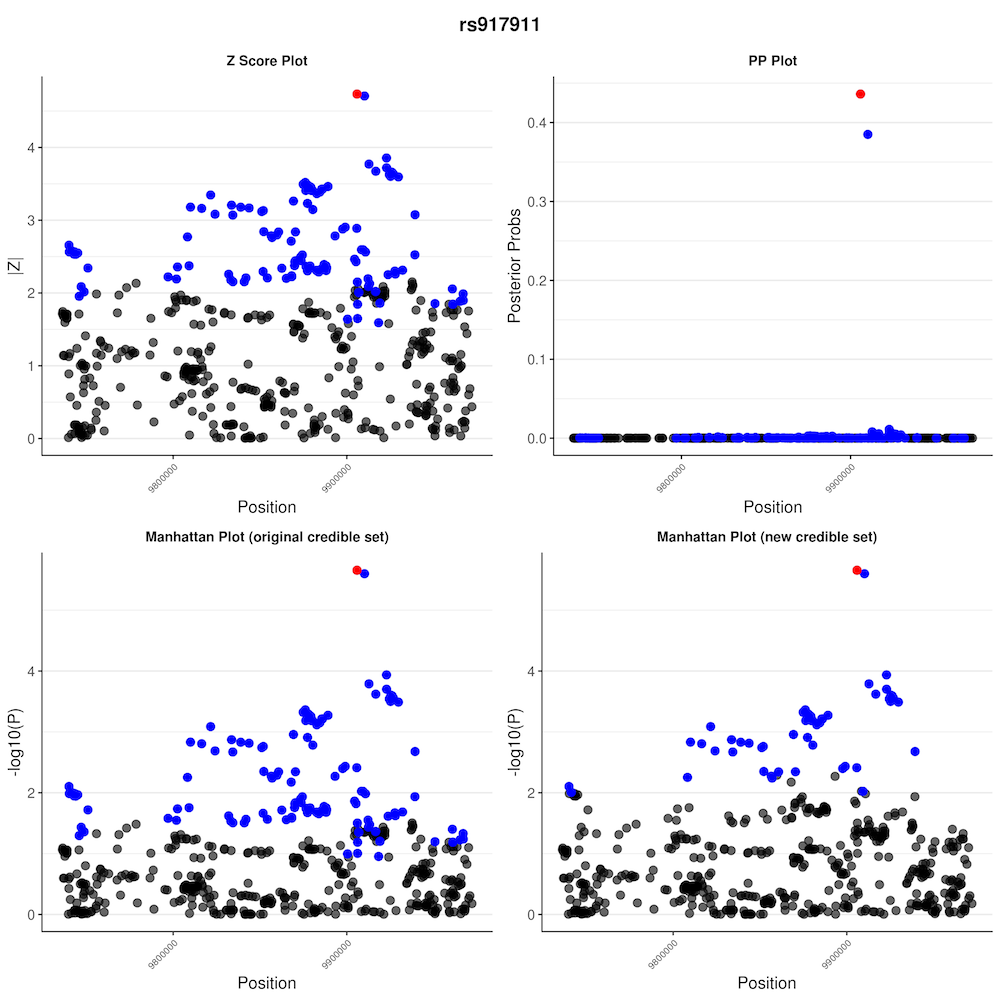

Supplement: S2 File — Zip file containing Z-score plots, PP plots and Manhattan plots for the 39 T1D association regions analysed. (ZIP) [file pcbi.1007829.s015.zip › S2_file/rs917911.png]

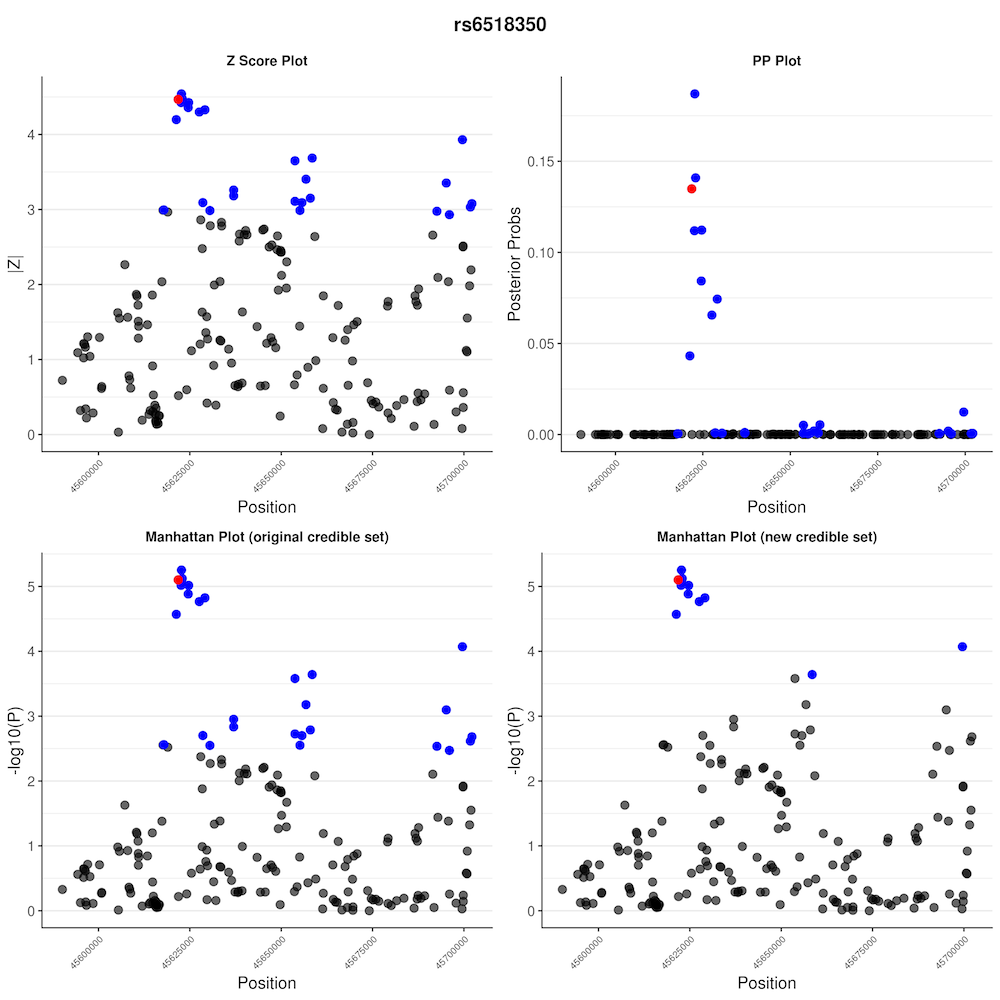

Supplement: S2 File — Zip file containing Z-score plots, PP plots and Manhattan plots for the 39 T1D association regions analysed. (ZIP) [file pcbi.1007829.s015.zip › S2_file/rs6518350.png]
