# Supplementary material for: Improving the coverage of credible sets in Bayesian genetic fine-mapping
Source: PLoS Comput Biol. 2020 Apr 13;16(4):e1007829. doi: 10.1371/journal.pcbi.1007829 (PMC7179948; doi:10.1371/journal.pcbi.1007829)
Supplement: Attachment — Submitted filename: responses.docx [file pcbi.1007829.s021.docx]

Reviewer #1: The authors argue that Bayesian credible sets of causal SNPs, computed during fine mapping of GWAS hits, tend to overestimate their coverage. Bias arises both by the fixed nature of a genetic effect at a given locus, and from selection of regions for fine mapping according to statistically significant association. An algorithmic method is proposed to estimate the magnitude of bias and adjust the coverage probability accordingly. An algorithm is presented to re-estimate credible sets with pre-specified coverage, and is shown to reduce the size of credible sets in several regions associated with type-1 diabetes. The paper raises some interesting points and should be of broad interest. It is refreshingly well written and low on typos.

Major point

1. The paper hinges on the understanding of the term “coverage”. While a frequentist understanding is alluded to in the introduction, the authors take a particular interpretation that may not be universally held. Specifically, the authors ask how often the credible set for the specific region under study covers the causal variant, given that it has been followed up. Here the fixed effects view is justifiable, and the bias of the standard calculation is shown (fig 1A right). However, given that credible sets are generated for many regions, both within and between studies (as the authors have done so themselves in the applied example), we can equally ask how often the credible set covers the causal variant, on average across all regions. This is an equally valid frequentist version of the question “what is the probability that the causal variant is contained in the credible set?” Here there is much less bias (fig 1A left). The bias by selecting significant regions is also open to interpretation. For regions not followed up, we effectively have estimated PP of zero for all SNPs and an empty credible set, which does not include the causal variant. Averaging over these regions as well as those explicitly followed up, the coverage probabilities would be unbiased. Although not a declared Bayesian, I am inclined to favour these broader understandings of coverage since the random effects scenarios are more common than the repeated sampling ones in practice. The authors are welcome to their view but should discuss this key point of interpretation in the introduction and discussion. I also suggest a change in terminology from “corrected coverage estimate” to “conditional coverage estimate” to reflect that it is only a correction under an interpretation that conditions on the specific region, phenotype and population being studied.

Indeed, the standard frequentist definition of “coverage” is the proportion of times an interval or set contains the true value **over all trials**. Our aim with Fig 1A is to show that the good coverage properties across all trials do not hold when we condition on a specific value of beta, in order to explain why such properties also do not hold when we condition on some level of significance, because this implies conditioning on beta (albeit not via any hard threshold). We think this point is important because in practice GWAS regions are only fine-mapped conditional on achieving some significance, and therefore it is the coverage within this set that we think is important. We do not believe that including all non-fine-mapped regions with a PP=0 would remove the bias, because the PP at the causal variant estimated by the fine mapping procedure, if it were applied, would not be exactly 0, and thus the effective estimate of 0 is itself biased. It seems unlikely that the two biases (across fine-mapped and non-fine-mapped regions) would exactly cancel each other out given the number of regions reaching fine-mapping significance levels varies across studies.

This distinction between coverage over all regions, and across only fine-mapped regions is important, and we now stress this distinction more clearly in the Introduction:

P2 L34-41

“The “coverage" of credible sets refers to the probability that the causal variant is contained in the credible set. Coverage is typically understood to refer to that probability taken over all possible realisations of the data. However, fine-mapping is conventionally only performed on a subset of genomic regions that contain at least one SNP with a P value of association below some threshold (for example genome-wide significance). We therefore examine *conditional coverage* here, defined as the probability that the causal variant is contained in the credible set, conditional on some aspect of the observed data, e.g. that it has been selected for fine-mapping.”

To make this absolutely clear, we use *conditional coverage* in place of *coverage* throughout, when we refer to this non-standard definition. We have also used the terminology “adjusted coverage estimate” in place of “corrected coverage estimate”. We felt that while “conditional coverage estimate” made perfect sense in the context of this discussion, it could be unclear in future, because it is not clear *what* is being conditioned on. We hope that “adjusted coverage estimate” strikes the right balance between addressing your concerns and using terminology that shows we are altering the value of something.

Minor points

2. P5 L139 “effect size” – it’s an expected Z-score that is derived, which is not the same as an effect size.

This has been corrected in the manuscript.

3. P5 L151 “Our method relies on MAF and SNP correlation data” – I don’t see how MAF information is needed to simulate correlated Z scores. It was used to simulate test data for the method, but doesn’t seem necessary for the method itself.

We did not make the connection clear. Our method calculates Bayes factors using Wakefield’s Approximate Bayes Factor (ABF) derivation. The calculation of ABFs requires effect estimates (e.g. log OR), their variances (V), and the prior variance of estimated causal effect size (W). To maximise flexibility of our method and require only P values (which we transform to Z scores), we approximate V by

$$V = 1/(2*N*MAF*(1-MAF)*s*(1-s))$$

where $N$ is the total sample size and $s$ is the proportion of cases.

If the user supplies V, then the MAFs are not required, but if only P values or Z scores are supplied (as in our simulations) then MAFs are needed to convert these to PPs.

We now explain this more clearly in the manuscript.

P12 L409-411

“If $V_{i}$ is unknown, we approximate it using

$$V_{i}= 1/(2*N*MAF_{i}*(1-MAF_{i})*s*(1-s))$$

where$N$is the total sample size,$s$ is the proportion of cases and $MAF_{i}$ is the $MAF$for SNP $i$.”

4. P7 L222 “the claimed coverage estimates are too low” – this seems to be based simply on comparing the sizes of the standard and corrected credible sets. Without knowledge of the actual causal variants, we can’t be sure that the corrected sets have the desired coverage.

Yes, this is correct. The sentence has been rephrased.

P8 L241-242

“The results match our previous findings - that the claimed coverage estimates are often lower than the adjusted coverage estimates.”

5. In fig S1 the N(0,0.2^2) actually looks like quite a poor fit to the GWAS data. Would smaller bin sizes improve the visual fit, or is this just a poor model? The choice of prior for the ABF is another factor that could bias the coverage estimate, and this ought to be discussed.

Yes, $N(0,W)$with $W=0.2^{2}$is indeed a poor fit to the GWAS data and increasing the bin size does not improve the fit (see figure below). This finding is not novel, for example Walters et al., (2019) (<https://onlinelibrary.wiley.com/doi/full/10.1002/gepi.22212>) find that a Laplace model fits GWAS top hits better than the normal distribution.

We include Fig S1 to show where our selected fixed effect size values (OR = 1.05 and 1.2) lie on the distribution. We believe discussing the poor fit of this distribution is outside the scope of this research as we are primarily interested in credible set coverage and have shown that when simulating from this distribution, the PPs are well calibrated (Fig 1A left). We update the word “modelled” in the manuscript which incorrectly suggests that our selected fixed effect size values were chosen as a fit to the retrospective GWAS data.

P3 L90

“We examined the distribution of genome-wide significant lead-SNP effect sizes from data deposited in the GWAS catalogue (Buniello, 2019) to select representative point distributions to use in our analysis (S1 Fig).”

We have amended the legend for Fig S1 to recognise the poor fit.

“S1 Fig. Distributions of absolute effect sizes. (A) Histogram of absolute effect sizes sampled from the prior, β ∼ $N(0,0.2^{2})$ (B) Histogram of absolute effect sizes of the lead-SNP in genome-wide significantly associated regions from case-control studies deposited on the GWAS catalog. Blue curve overlaid is for $N(0,0.2^{2})$distribution, black dashed line is where β = log(1.05) and red dotted line is where β = log(1.2). The x axis has been truncated to remove extreme values. The distributions are quite different, resulting from censoring of smaller $\beta$which are less likely to be associated with a genome-wide significant P value (see Fig 1).”

In relation to the choice of prior for the ABF, we observe that our findings are mostly robust to the choice of W (see figure below). Since $W=0.2^{2}$ is the typical choice in a case-control GWAS, we chose this value for the default in the software, however it is included as an optional parameter which can be easily specified by the user if they wish. The figure below has now been included as a supplementary figure (Fig S7) in the manuscript.

P5 L165

“Our findings are robust to the choice of value for$W$in the causal variant effect size prior, $N(0,W)$(Fig S7).”

Typos

6. P2 L10 “loci” -> “locus”

7. P12 L406 “was” -> “were”

8. P12 L410 “it’s” -> “its”

9. P13 L429 and L431 “these” -> “them”

All of the typos have been corrected in the manuscript.

---

Reviewer #2: This was an interesting and topical manuscript. My overall enthusiasm for the manuscript was slightly diminished by the fact it was unclear how serious a problem the issue raised (namely that of the conservative coverage of fine-mapped variants) would be in practice. In particular, Figure 1C (rightmost plot) suggested that for signals reaching the de-facto genome-wise significance threshold of around 10-8, there is in fact little discrepancy between the claimed and true coverage.

[Please note that our “correction” has now been renamed to “adjustment”].

Yes, the discrepancy is smaller, but is still biased towards conservative estimates in the $10^{-8}$to $10^{-12}$bin, with relatively large variability between estimates (compared to the results from our adjustment method) - see Fig 2A. The bias and variability increase in regions of higher LD - see Fig S2C. We also found many examples in the literature where researchers are fine-mapping in regions not quite reaching genome-wide significance (e.g. using a threshold P < 10^-6^), where there is larger discrepancy between the claimed and true coverage.

Given this fact, I am slightly unclear which analyses presented in the manuscript support the statements made in the Abstract: “We show that this is because fine-mapping data sets are not randomly selected from amongst all causal variants, but from amongst causal variants with larger effect sizes” and in the Discussion: “We show that this is because fine mapped regions are not a random sample as assumed by fine mapping methods, but the subset of regions with the most extreme P values.”? Surely Figure 1C (rightmost plot) suggests that the regions with the most extreme P values (which will likely correspond to those with the larger effect sizes) are exactly those regions that will NOT be affected by the problem?! As these regions in fact show little discrepancy between the claimed and true coverage?

This is an important and perhaps subtle point that we did not clearly communicate. We argue that causal variant effect sizes for fine mapped regions are not sampled from the prior $N(0,0.2^{2})$ (even if true causal variants were), but from a censored subset where some *a priori* defined significance threshold is reached. The effect of this is negligible for the most extreme effects, because there is very little censoring here - they are nearly always associated with strong P values. But in the “just” fine-mappable range, this effect is much more pronounced, and of course these effects are more commonly found by GWAS studies - the -log10(P) value distribution from any individual study is invariably right-skewed.

It is difficult to explain this clearly in the abstract due to space, but we have adjusted the Discussion.

P9 L284-288

“We show that this is because fine-mapped regions are not a random sample as assumed by fine mapping methods, but the subset of regions with the most extreme P values. Note that the effect of this censoring is actually most severe where P values just exceed the significance threshold, explaining why our adjustment has the most effect in the range of 10^-12^ < P < 10^-4^.”

Additional comments:

Page 2, Line 23: reference [7] – it might also be worth referencing Ayers and Cordell (2010) https://onlinelibrary.wiley.com/doi/full/10.1002/gepi.20543 here, who I think also investigated the use of penalised regression for fine mapping (prior to Valdar et al. 2012)?

Yes, apologies for missing this out. This reference has now been added.

Page 3, lines 70-81 (see also page 9 lines 293-294): this issue of simulating from the prior as opposed from a fixed beta requires more detailed discussion/explanation. By “simulating from the prior” do you mean you resample the value of beta within each simulation replicate? I cannot see this as a correct approach (though I don’t mind you presenting it, provided it is made clear that it is not correct!) Within any given data set, the effect size at any particular causal variant surely takes a specific value (that may be considered to have been drawn from some distribution before the study started). If one is trying to investigate the properties of some analysis approach under repeated sampling of the dataset, surely one should indeed leave the effect size at the variant fixed at that sampled value?

This is a bit different from the idea of simulating the effect sizes of multiple different causal variants across the genome from a distribution (a model that underpins a lot of the recent work on SNP=based heritability estimation, linear mixed models, GCTA etc.), Depending on context one might want to resample the random effects, although I think would argue that for analysis of a specific data set, the effects (one sampled) should be considered fixed. It is also a bit different from a random effects meta-analysis whereby one could consider different studies to have genuinely different effect sizes (albeit sampled from a distribution with a common mean). Anyway, I think this all needs a bit more clarification and discussion.

“Simulating from the prior” means that **in each simulation** we sample beta from $N(0,0.2^{2})$and then use this value in each simulation replicate to evaluate the empirical true coverage, not that we resample the value of beta within each simulation replicate. You are correct in that “within any given data set, the effect size at any particular causal variant surely takes a specific value (that may be considered to have been drawn from some distribution before the study started)” and this is exactly our simulation protocol.

We recognise the wording is ambiguous, so have clarified the following parts of the manuscript.

P3 L82-86

“We therefore simulated association studies that reflect the underlying Bayesian single causal variant fine-mapping model exactly, such that in each simulation, causality was randomly selected across all SNPs and the causal effect size (β = log odds ratio) was sampled from$N(0,0.2^{2})$and then fixed at its sampled value to calculate an estimate of the empirical conditional coverage.”

P10 L334-338

“In each simulation, causality was randomly allocated to one variant in the region and effect sizes were either (i) sampled from the prior ($\beta\sim N(0,0.2^{2})$) before being fixed at that sampled value to assess the empirical calibration of conditional coverage estimates, (ii) fixed at $\beta=log(1.05)$or (iii) fixed at $\beta=log(1.2)$.”

Page 6, lines 190-191: This finding (that a corrected 90% credible set could be constructed using a threshold value of 0.781) is presumably specific to this particular example? i.e. this calculation would need to be repeated for any given GWAS that one was analysing, if one wished to calculate the appropriate threshold? Could be clarified.

Yes, this result is specific to the walk-through example. The section has been updated to illustrate that this result is specific to the particular example.

P7 L207-209

“We used the root bisection method (Greene, 1992) to iteratively search for the smallest threshold value that yields a credible set with accurate coverage of the causal variant. In this example, we found that a corrected 90% credible set could be constructed using a threshold value of 0.781.”

P7 L214-217

“Simulations confirmed that the empirical coverage probabilities of adjusted credible sets created using this method are accurate, such that on average the empirical estimate of the true coverage of a corrected 90% (or 95%) credible set is indeed 90% (or 95%) (Fig S8).”

Page 7 lines 215-225: It would be informative to state what significance levels were achieved in the 40 genomic regions interrogated. Given that your approach is successful in reducing the size of the 95% and 99% credible sets, it suggests there must indeed be some degree of conservative coverage for this data set when using standard approaches. This, combined with the observation made above (that for signals reaching the de-facto genome-wise significance threshold of around 10-8, there is in fact little discrepancy between the claimed and true coverage) suggests that many of the the 40 regions must not in fact have reached genome-wide significance levels?

Supplementary Tables 1 and 3 have been updated to include a column for the P value of the lead-SNP. The P values range from ~$10^{-7}$to $10^{-100}$. Although claimed and adjusted coverage estimates are largely unbiased in very high-powered regions ($P<10^{-12}$), the variance is tighter for adjusted coverage estimates (Fig 2A versus Fig 2B).

There are 16 regions with $P<10^{-12}$ in the T1D analysis. Of these, 6 were not adjusted because the threshold value (0.95) was within the 95% confidence interval for the adjusted coverage estimates of the original 95% credible sets, or because the credible set already contained only a single variant (and the adjusted coverage estimate was $>0.95$ implying that more variants did not need to be added to the set). Of the remaining 10 regions, 4 credible sets did not change after adjustment and 6 credible sets did change after adjustment (dropping 1 variant). The figures below show (1) the distribution of lead-SNP P values for regions where the credible sets changed and where the credible sets did not change and (2) the lead-SNP P values against the number of variants removed from the set after adjustment, with a grey dashed line at $P=10^{-12}.$ Indeed, sets which changed did tend to have larger minimum P values. For the 6 regions with $P<10^{-12}$and where the credible set size changed after adjustment, we include the change in the number of variants in the credible set after adjustment and the PP of the variant that is dropped from the credible set.

Now we ask, “why do the adjusted credible sets for these high powered regions drop variants as opposed to some dropping variants and some gaining variants upon adjustment, as our simulation results would imply”?

The plot on the right shows the adjusted coverage estimates against the claimed coverage estimates for the T1D analysis with the 6 regions with $P<10^{-12}$and where the credible set size changed after adjustment shown in red. We clearly see that the “error” (difference between claimed and adjusted coverage here, as we do not know the empirical coverage) can be either positive or negative, as shown in our simulation analysis.

The error is more likely to be “negative” (claimed<adjusted) when the claimed coverage is only slightly higher than the target coverage/threshold value. We also observe that this error is more likely to be “positive” (claimed>adjusted) when the claimed coverage is much higher than the target coverage/threshold value.

This suggests that the error in claimed coverage is systematically different between sets for which claimed coverage just exceeds threshold, versus those whose claimed coverage shoots past threshold (because the last added SNP has a larger PP). Similar patterns are also visible in our simulated data:

In regions with adjusted coverage > claimed coverage, it is easy to understand how a variant can be dropped, still keeping the adjusted coverage > target coverage/threshold (here 95%).

Where adjusted coverage < claimed coverage, we agree it is less obvious to see that we could safely lose a variant, but the effect is explained by considering the results for the 3 regions where this happens are shown below:

Even though claimed coverage (column “claimed_cov”) > adjusted coverage (column “adjusted_cov”), the claimed coverage is so high that it is possible to find a smaller (by 1 SNP) set with claimed coverage closer to target.

We found it helpful to step through this analysis carefully to respond to this query, and think that it is important to summarise these observations in the manuscript.

P8 L247-261

“Generally, credible sets were more likely to be subject to change where minimum P values were larger (Fig S12A). In 16 "high-powered" regions (minimum $P<10^{-12}$), 6 were not adjusted because the threshold value (0.95) was within the 95% confidence interval for the adjusted coverage estimates of the original 95% credible sets, or because the credible set already contained only a single variant (and the adjusted coverage estimate was >0.95 implying that more variants did not need to be added to the set). Of the remaining 10 regions, 4 credible sets did not change after adjustment and 6 credible sets did change after adjustment (dropping 1 variant). We observed that this happened in two ways (Fig S12B). In 3 cases, the adjusted coverage was greater than the claimed coverage, allowing a variant to be dropped. In the other 3 cases, the claimed coverage was near 1 (above 99% for a 95% target), and so there was "space", with a tighter confidence interval about the adjusted coverage, for a lower target to be used whilst still maintaining adjusted coverage > 95%. The full results for these 6 regions (with minimum $P<10^{-12}$ and where the credible set changed after adjustment) are shown in S5 Table.”

Page 8 lines 261-270: You are correct that methods such as GUESSFM and FINEMAP and JAM produce more complicated outputs in terms of posterior probabilities for various configurations of causal variants. However I am not sure that *that much* post-processing is necessary to investigate these approaches. In particular, FINEMAP versions 1.3 and 1.4 already produces lists of credible SNPs for the best single-SNP configuration, the best two-SNP configuration, the best 3-SNP configuration etc. etc.c It would certainly be interesting to investigate whether these lists of credible SNPs are subject to the same issue that you have highlighted for single-variant fine mapping approaches.

Thank you for this comment. We have rephrased the paragraph to show that newer software versions can indeed produce credible sets for the best X-SNP configurations. We agree that investigating whether credible sets formed from alternative multi-SNP statistical fine-mapping methods (FINEMAP/JAM/SuSiE etc.) show similar coverage biases is an interesting extension, however we believe that this is outside the scope of this current manuscript, which focuses only on the results from single causal variant Bayesian fine-mapping, but we suggest that this investigation will be interesting to pursue in future.

P9 L307-312

“The output from these methods are posterior probabilities for various configurations of causal variants, and therefore the grouping of SNPs to distinct association signals must often be performed post-hoc to obtain similar inferences to that of single causal variant fine-mapping. Newer software versions (e.g. FINEMAP v1.3 and v1.4), however, are now able to produce lists of credible SNPs for the best multiple causal variant configurations. Thus, an interesting extension to this research would be to investigate whether the credible sets from multiple causal variant fine-mapping are subject to the coverage biases exhibited in single-causal variant fine-mapping.”

Page 10 line 326-327: I am not sure that I follow why defining coverage for regions containing > 2 CVs is particularly problematic? Would you not just define it as the frequency with which a credible set contained at least one of the CVs (as you did for regions with 2 CVs)? I could imagine that the more CVs there are in a region, the easier it would be for *any one* of them to “get chosen” into the credible set – I’m not sure what effect this would have on the coverage? However this issue would presumably pertain to the 2 CV situation as well?

Yes, this is an interesting point. If there are multiple causal variants in a region, multiple possible definitions of “coverage” exist. For example, “coverage” could be defined as the probability that all causal variants are captured in the set, the probability that a CV chosen at random is captured in the set or the probability that at least one of the causal variants is captured in the set. If we adopt the latter definition (as you suggest, and as we use in our 2 causal variant analysis) then we are assuming that a credible set which contains, say, all 5 of the causal variants is “just as good” as a credible set that only contains one of the 5 causal variants - which we are uneasy with. We also do not expect that claimed coverage under this definition can ever match empirical coverage, since there is always more chance to select at least CV rather than to select a specific CV. This discrepancy in interpretation would grow as the number of causal variants in the region increases, as there are now more chances to include a causal variant in the set.

Our aim of including the 2 causal variant analysis is not to show how to fine-map multiple CV regions but, given that the single causal variant method *is currently used* in such situations, to show that our method is no less appropriate than the standard method when there is more than one causal variant in a fine-mapping region. Thus, since the Bayesian single causal variant fine-mapping method explicitly hinges on the assumption that exactly one variant in the region is the causal variant, we hope that researchers are not using this fine-mapping method (and therefore our adjustment) if they believe this assumption does not hold. Nevertheless, if this single causal variant fine-mapping approach is being used in regions that violate this assumption then we show that our method performs no worse than the standard method.

The manuscript has been adjusted to demonstrate this point.

P6 L189-193

“Whilst we do not recommend using the Bayesian single causal variant fine-mapping method (and therefore our adjustment) when the single causal variant assumption is violated, we show that if this is the case then our method performs no worse than the standard method, when considering capturing at least 1 causal variant as most relevant.”

P10 L370-374

“If multiple CVs are present in a region then multiple possible definitions of conditional coverage exist, and as the number of CVs in a region increases, the number of possible definitions and the discrepancies in their interpretation increases. For this reason, we chose not to analyse conditional coverage properties in regions containing >2 CVs.”

Page 11 line 368: Rephrase “at the causal SNP’s position which takes the value $\mu$” as “at the causal SNP’s position where the value of $z_i$ takes the value $\mu$” ? (That is provided you define the $i$th element of $Z_J$ as $z_i$, which you might need to do earlier).

The notation $z_{i}$ is already used to define the i’th position of the observed Z score. Therefore, and to avoid introducing additional notation, the sentence has been changed to:

“where $Z_{J}$has length equal to the number of SNPs in the region, and all elements take the value 0 except at the position of the causal variant, which takes the value $\mu$.”
